# General population perspectives of dementia risk reduction and the implications for intervention: A systematic review and thematic synthesis of qualitative evidence

**DOI:** 10.1371/journal.pone.0257540

**Published:** 2021-09-17

**Authors:** Eleanor Curran, Terence W. H. Chong, Kali Godbee, Charles Abraham, Nicola T. Lautenschlager, Victoria J. Palmer

**Affiliations:** 1 Academic Unit for Psychiatry of Old Age, Department of Psychiatry, Melbourne Medical School, Faculty of Medicine, Dentistry and Health Sciences, University of Melbourne, Parkville, Victoria, Australia; 2 NorthWestern Mental Health, Royal Melbourne Hospital, Parkville, Victoria, Australia; 3 St Vincent’s Hospital Melbourne, Kew, Victoria, Australia; 4 Department of General Practice, Melbourne Medical School, Faculty of Medicine, Dentistry and Health Sciences, University of Melbourne, Parkville, Victoria, Australia; 5 School of Psychology, Deakin University, Geelong, Victoria, Australia; 6 The Centre for Digital Transformation of Health, Faculty of Medicine, Dentistry and Health Sciences, University of Melbourne, Parkville, Victoria, Australia; University of Edinburgh, UNITED KINGDOM

## Abstract

**Background:**

Evidence for the potential prevention of dementia through lifestyle risk factor modification is growing and has prompted examination of implementation approaches. Understanding the general population’s perspectives regarding dementia risk reduction is key to implementation. This may provide useful insights into more effective and efficient ways to help people change relevant beliefs, motivations and behaviour patterns. We conducted a systematic review and thematic synthesis of qualitative evidence to develop an integrated model of general population dementia risk reduction perspectives and the implications for intervention in research and implementation contexts.

**Methods and findings:**

We searched electronic databases, supplemented by lateral search techniques, to identify studies published since 1995 reporting qualitative dementia risk reduction perspectives of the non-expert general population who do not have dementia. Thematic synthesis, incorporating an expert panel discussion, was used to identify overarching themes and develop an integrated model to guide intervention to support individuals to adopt and maintain dementia risk reduction behaviour patterns. Quality of included studies and confidence in review findings were systematically appraised. We included 50 papers, reflecting the views of more than 4,500 individuals. Main themes were: 1) The need for effective education about a complex topic to prevent confusion and facilitate understanding and empowerment; 2) Personally relevant short- and long-term benefits of dementia risk reduction behaviour patterns can generate value and facilitate action; 3) Individuals benefit from trusted, reliable and sensitive support to convert understanding to personal commitment to relevant behaviour change; 4) Choice, control and relevant self-regulatory supports help individuals take-action and direct their own progress; 5) Collaborative and empowering social opportunities can facilitate and propagate dementia risk reduction behaviour change; 6) Individual behaviour patterns occur in social contexts that influence beliefs through heuristic processes and need to be understood. Findings indicate that, for intervention: 1) education is key, but both content and delivery need to be tailored; 2) complementary interventions to support self-regulation mechanisms and social processes will increase education effectiveness; 3) co-design principles should guide intervention design and delivery processes; 4) all interventions need to be supported by context-specific data.

**Conclusions:**

This systematic review and thematic synthesis provides a comprehensive, integrated model of the dementia risk reduction perspectives of the general population and intervention approaches to support behaviour change that can be applied in clinical trial and real-world implementation settings. Findings extend existing knowledge and may assist more effective intervention design and delivery.

## Introduction

**Fig 1:**
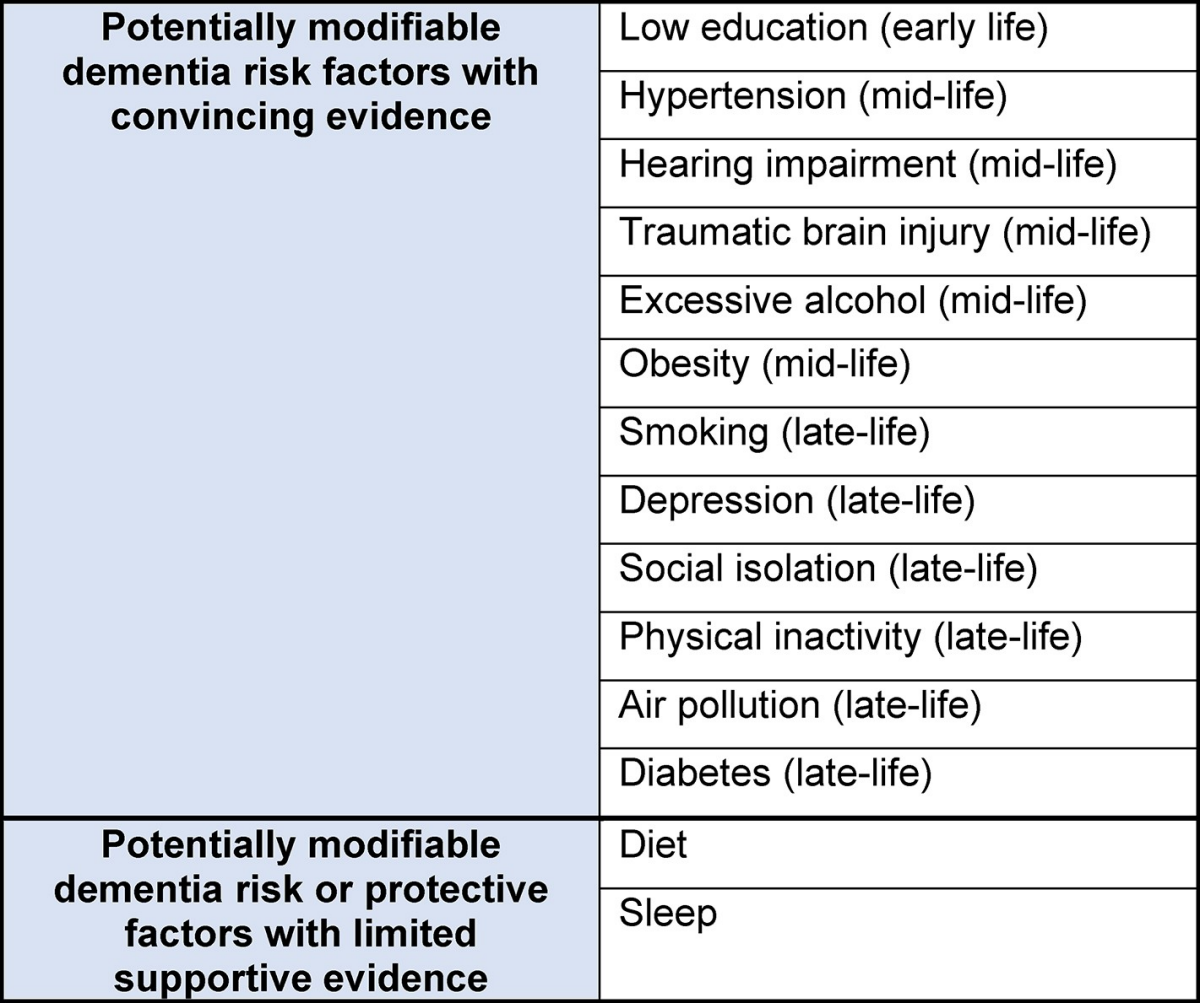
Dementia modifiable risk factors. Modifiable risk factors from major guidelines and strength of supportive evidence. Adapted from [3].

Dementia has been described as one of the defining public health challenges of the 21^st^ Century [1] and disease-modifying treatments remain elusive [2]. However, recent seminal reviews indicate that up to 40% of late-onset dementia could potentially be prevented or delayed by addressing modifiable risk factors (MRFs); including optimising vascular risk-factors and behaviour patterns such as engaging in physical activity and following a Mediterranean diet [3–6] (see Fig 1 for key dementia MRFs).

Models of dementia prevention are complex and, to date, largely based on observational studies that have not produced consistent findings (e.g. [4, 6]) and reverse causality may contribute to some findings (e.g. [7, 8]) (See Fig 1 for strength of supporting evidence for individual MRFs). In addition, how the timing of risk or protective factor exposure and interactions between different risk and protective factors (including non-modifiable risk factors such as genetic vulnerabilities) influence outcomes remains unclear. The optimal combinations, doses and durations of interventions are as yet unknown [3, 9, 10]. These complexities have likely contributed to randomised controlled trials (RCTs) finding only modest outcome benefits, at best (e.g. [11–13]). More convincing effectiveness data from RCTs is needed, however, this will be challenging to obtain, particularly because of the long time-frames between interventions and dementia outcomes. Given these complexities and the potential of dementia risk reduction, the World Health Organisation (WHO) has called for immediate implementation in clinical practice and through targeted public health programs, concurrent with further development of the evidence base [14].

Optimising adoption and maintenance of behaviour patterns that can reduce dementia risk (by reducing exposure to dementia risk factors and increasing protective factors) is key to both successful implementation and to advancing the intervention evidence base by improving adherence to interventions [15]. Many risk and protective factors for dementia are themselves patterns of behaviour (e.g. a physically inactive lifestyle). Others have strong links to behaviours, such as links between uncontrolled mid-life hypertension and behaviours such as adhering to guidelines for ‘check-ups’ of vascular risk factors and to prescribed medications. Improving both types of behaviour patterns is important and recent modelling evidence has demonstrated the potential for substantial impacts on future dementia incidence [16].

Thus, understanding how to most effectively improve relevant individual behaviour patterns is crucial for design of both future RCTs and implementation research/processes. Numerous models of health behaviours recommend targeting the mechanisms that underpin behaviour and highlight knowledge, attitudes, motivation, skills, habit regulation and other self-regulatory processes, as well as social and environmental factors that shape behaviour patterns [17–21]. Several dementia risk reduction trials (e.g. [13]) have utilised these theories, such as social cognitive theory [22], and taxonomies designed to match potential intervention components (including change techniques and intervention delivery modes) to underlying change mechanisms (e.g. [23, 24]. However, health and behavioural outcomes in dementia risk reduction have been mixed [13, 25]. Further work is needed to identify the combination of behaviour changes required, and to specify underpinning regulatory mechanisms and optimal matching change techniques to be incorporated in interventions.

Individual perceptions provide important insights into the regulatory mechanisms underpinning dementia risk reduction behaviour patterns [26]. To date, however, only evidence regarding the general public’s knowledge of the potential for dementia risk reduction and of individual MRFs has been reviewed. Limited knowledge was identified in three recent systematic reviews and one scoping study [27–30]. One review found that knowledge was improving (slightly) over time [28] and another found an association between better knowledge and access to information or educational materials regarding dementia and dementia risk factors [30]. None considered broader individual perceptions, which are important to understanding why poor knowledge persists and to optimised interventions that target both knowledge and other mechanisms [26]. Furthermore, qualitative data are important to understanding the complexity of individual perspectives, but only one of the existing reviews included synthesis of qualitative data and did not report any qualitative themes in their findings [30].

A more analytical and integrated picture of the range of individual perspectives about dementia risk reduction, the regulatory mechanisms they suggest are central to relevant behaviour patterns, and their implications for intervention could better support more effective intervention design and delivery [31]. There is a growing body of qualitative literature examining a broad range of general population perspectives, and synthesis of these data can facilitate the comprehensive and nuanced understanding required [32]. However, to our knowledge, this has not previously occurred.

In this context, we conducted this synthesis of qualitative general population perspectives of dementia risk reduction to advance conceptual understanding of the mechanisms underpinning relevant behaviour patterns and the implications for intervention, to inform more effective intervention design and delivery. The objective of our study was to systematically identify and synthesise qualitative literature on the dementia risk reduction perspectives of the general population. We aimed to develop an integrated model of key concepts for mechanisms underpinning behaviour patterns and suggested intervention approaches to support individuals to adopt and maintain dementia risk reduction behaviour patterns.

## Methods

The study protocol was pre-specified and prospectively registered with the International Prospective Register of Systematic Reviews (PROSPERO) (CRD42020165448). Reporting follows PRISMA guidance [33] and the more specific statement, Enhancing Transparency in the Reporting of Syntheses of Qualitative Research (ENTREQ) [34] (For PRISMA and ENTREQ checklists see S1 File). Our methods followed Cochrane Qualitative and Implementation Methods Group (GQIMG) guidance for conducting qualitative evidence syntheses that aim to produce clear statements of qualitative findings to inform decision making contexts such as intervention design [35].

### Search strategy and selection criteria

**Fig 2:**
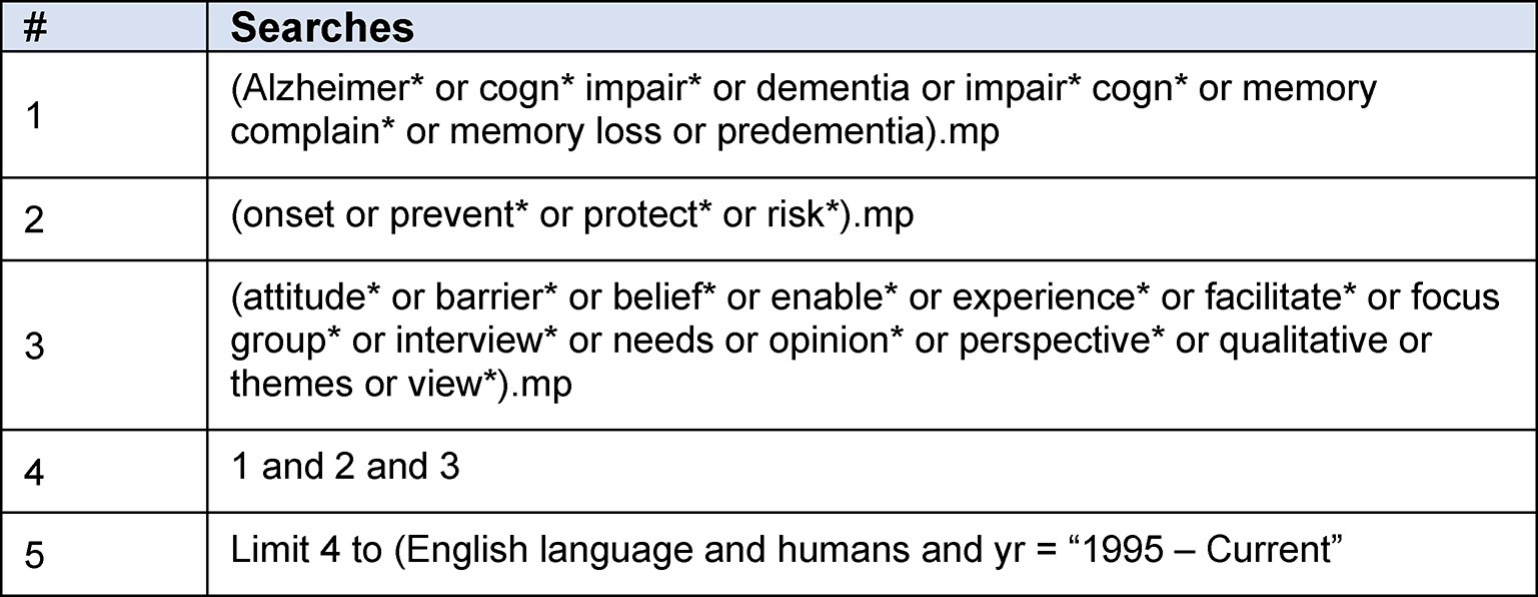
Example search strategy. Search string used for MEDLINE (Ovid).

The search strategy aimed to identify studies reporting dementia risk reduction perspectives of the general public. Searches were undertaken in December 2018 and updated in December 2019 prior to study selection. Four databases were searched (MEDLINE, PsycINFO, CINAHL and Embase) using a pre-specified strategy designed with the support of a research librarian. Searches employed terms for ‘dementia’, ‘prevention’, and ‘views’, ‘attitudes’ or ‘beliefs’ (see Fig 2 for an example search strategy and S2 File for complete search strategy). We did not specify population in the search strategy as diverse terms are used to refer to the general population and we sought to include studies with mixed populations where relevant data could be extracted. Hand searching of reference lists, citation tracking and expert consultation supplemented database searches.

Studies were included if they were primary studies reporting qualitative data regarding the dementia risk reduction perspectives of the non-expert general population without dementia. Diverse approaches to data collection and analysis have been used in this field, so any qualitative design was eligible, including written surveys and mixed-methods designs with separately reported qualitative findings, if recognised qualitative data analysis and appropriate data collection methods were used. Studies were excluded if they only reported the perspectives of health professionals or other experts, students or people living with dementia. Studies with mixed populations were only eligible if results for participants meeting this definition of the general population were reported separately. Qualitative process evaluations of intervention trials were eligible if dementia risk reduction (or prevention) was identified as an outcome of interest. Peer-reviewed articles published in English between 1995 and December 2019 were included. Conference proceedings, letters, editorials and commentaries were excluded.

Following removal of duplicates, titles and abstracts were screened by one reviewer (EC or KG) with 10% independently screened by both reviewers to confirm consistent application of inclusion criteria. Full-texts of all potentially eligible articles were independently screened by two of three reviewers (EC, KG, TC) and all discrepancies resolved by discussion and consensus.

### Study quality assessment

Included studies were independently appraised by two reviewers (EC and TC) using an adapted version of the Critical Appraisal Skills Program (CASP) checklist [36] with discussion for consensus. The CASP checklist covers 10 domains: research aims; appropriateness for qualitative methodology; study design; sampling; data collection; reflexivity; ethical considerations; analysis; credibility; and value. We adapted the tool to increase utility by combining findings across each domain to grade studies overall. We did not exclude lower quality studies but appraised their impact on our findings through sensitivity analyses and considered quality of contributing studies when interpreting synthesis findings [37].

### Data extraction and analysis

Data were extracted using a pre-prepared template. Key characteristics and contextual information for each included study and all relevant participant quotations and author interpretations were extracted from the results/findings sections of papers. Other sections of papers were checked but further data only extracted if there were new findings to ensure extracted data more accurately reflected prominence of themes. Extracted data were imported into NVivo 12 software [38] for storing and coding.

Data were analysed using thematic synthesis [39]. This approach is recommended for understanding and interpreting beliefs relevant to designing and implementing complex interventions such as dementia risk reduction [32, 37]. It allows transparent integration of ‘thin’ and ‘richer’ data into hierarchical descriptive themes and generation of interpretive analytical themes that extend beyond the primary data to address the specific review questions.

The review team comprised all co-authors. Two reviewers (EC and TC) first inductively coded 10 randomly selected studies line-by-line independently, to identify concepts relevant to understanding the general population’s views about adopting and maintaining dementia risk reduction behaviour patterns. Through discussion of identified concepts an initial coding framework with agreed meaning and terminology was created. Coding consistency was established through re-coding the same papers (Κ > 0.8). The remaining studies were divided between the two reviewers, with discussion and addition of new codes as required. Codes were grouped according to conceptual similarities and differences to identify initial descriptive themes. A narrative summary was produced by one reviewer (EC), checked against the original data and refined by the review team for a consensus summary.

Sensitivity analyses assessed the impact of low-quality studies on identified descriptive themes, and the level of confidence in each descriptive theme was assessed using the Grades of Recommendation, Assessment, Development and Evaluation–Confidence in the Evidence from Qualitative Reviews (GRADE-CERQual) tool [37]. These assessments did not alter the consensus framework but were incorporated into the synthesis findings.

Finally, analytical themes about key concepts underpinning dementia risk reduction behaviour patterns and their implications for behaviour change interventions were generated through a panel discussion including six academic and clinical experts and further discussion within the review team. Descriptive themes were grouped, further interpreted and abstracted to form a final framework that both described and extended the original data. A summary model integrated key insights from analytical themes regarding mechanisms underpinning behaviour patterns and practical intervention recommendations. This was designed to provide guidance for intervention design and delivery processes.

### Updating of searches prior to publication

Prior to publication, we updated searches (July 2021) using the same strategy and databases as in the original searches. Study selection and appraisal processes were identical, except that one reviewer completed these (EC), with checking and agreement regarding additional included studies by a second reviewer (TC). There is limited guidance regarding approaches to updating qualitative syntheses. We applied an approach of comparing new data against the original analysis and extending this, based on recommendations for updating meta-ethnography [40] and recent similar updates of qualitative syntheses for similar questions to ours [41, 42]. We chose this approach as there had only been 18 months since original searches, in which no major methodological advances had occurred; and the additional studies we identified had applied similar methods and been conducted in similar contexts to those originally included [40]. Data that corroborated or added new insights to themes were integrated with the original synthesis to refine our interpretive analysis and model. If a major new concept had emerged we would have repeated processes to consider the implications for intervention.

## Results

**Fig 3:**
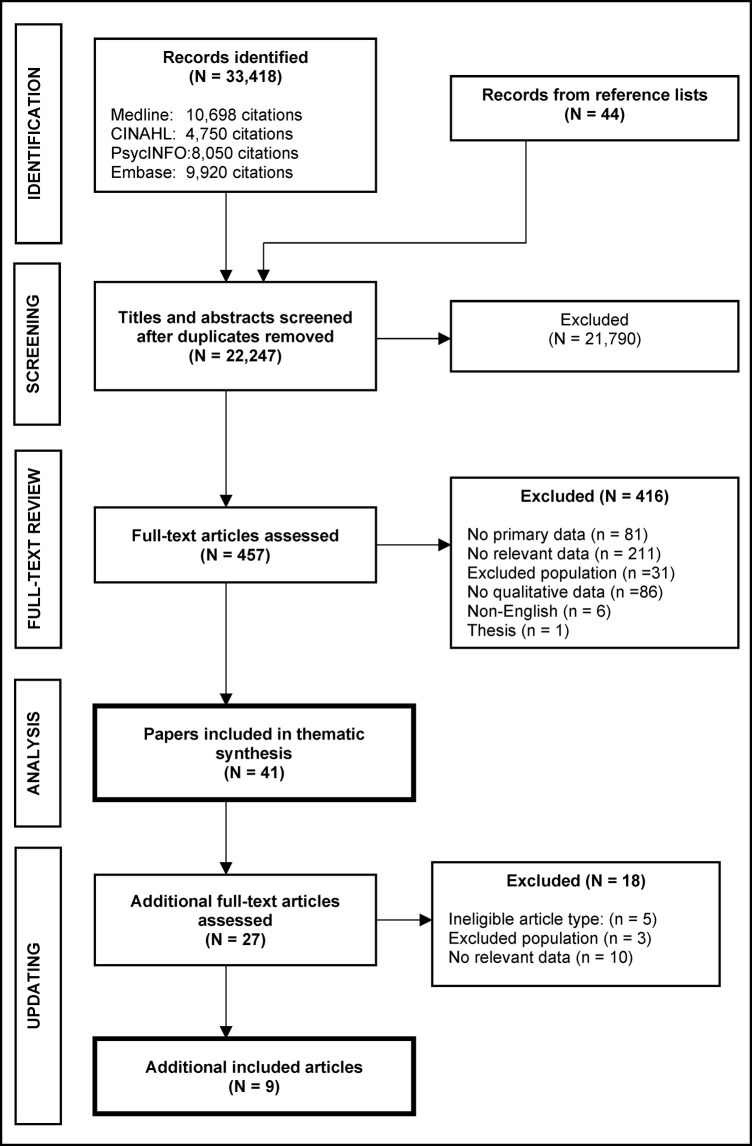
Study flow. Flow chart of study selection process.

Searches identified 22,203 individual papers from databases and a further 44 from reference lists. Following title and abstract review, 457 full-text papers were screened. Forty-one papers, describing the results of 37 individual studies, were included and synthesised [43–83] (Fig 3). From updated searches, a further 27 full-text papers were assessed and an additional nine papers met inclusion criteria [84–92] (Fig 3).

### Included studies

An overall summary of included studies from original searches is shown in Table 1 (S1 Table for detailed individual study characteristics). Combined, they reported the perspectives of approximately 3,637 individuals (one study only described an approximate number of participants) [77]. Most papers were published since 2010 and all were conducted in high income countries (HICs). While description was variable across studies, diverse populations and contexts were reflected, including a range of socioeconomic levels, rural communities and cultural groups. Study contexts included examining impacts of disclosure of dementia risk status (n = 10), evaluating dementia risk reduction interventions (n = 13), and examining views on healthy ageing (n = 16), or cognitive decline and dementia (n = 9). While not always specified, physical activity and diet behaviours were the most common behaviours of focus, but a wide range of other behaviours were also considered (for additional information see S2 File).

**Table 1:** Summary of studies included for original synthesis.

Context Information	
***Year of publication***	Range 1998–2019
	8 published prior to 2010
***Country*** *	UK *n* = 10
	US *n =* 20
	Europe (non-UK) *n =* 6
	Canada *n =* 4
	Australia *n =* 3
	Korea *n =* 1
	Japan *n =* 1
***Setting*** *	Community *n = 33*
	Specialist clinic *n = 7*
	Support group *n =* 1
	Not reported *n =* 1
***Study Design*** ^***$***^	Qualitative or qualitative descriptive *n = 17*
	Photovoice *n = 2*
	Mixed methods *n = 13*
	Ethnography *n = 3*
	Grounded theory *n = 2*
	Phenomenological *n =* 1
	Not reported *n = 3*
***Data Collection*** * ^***$***^	Individual interviews *n = 19*
	Focus groups *n = 15*
	Qualitative survey *n = 5*
	Sharing circles with photo elicitation *n = 1*
	Field notes *n = 2*
	Task groups *n = 1*
	Journal entries *n = 1*
	Ethnographic interviews *n = 2*
***Topics relevant to synthesis*** *	Views on dementia risk reduction as part of views on healthy ageing *n = 16*
	Views on dementia risk reduction as part of views on cognitive health and dementia *n = 9*
	Views on dementia risk reduction as part of views on genetic/pre-clinical testing *n = 10*
	Impact of caring for person with dementia *n = 9*
	Evaluation of dementia risk reduction intervention *n = 13*
**Participant Characteristics**	
***Age***	Range 18 –>90 y
	Majority middle-aged and older
***Included participant type with separate data reported*** *	General public or not reported *n = 24*
	Persons with SCD or MCI *n = 6*
	Carers of people with dementia or MCI *n = 13*
	Biological relatives of people with dementia *n = 6*
***Cultural identity*** ^***$***^	African American participants only *n = 2*
	Indigenous peoples participants only *n = 2*
	Asian participants only *n = 2*
	White participants only *n = 7*
	Mixed *n = 11*
	Not reported *n = 17*

Sample sizes refer to the number of studies.

*Some studies classified as more than one category.

^$^As reported by study authors.

US, United States; UK, United Kingdom; MCI, mild cognitive impairment; SCD, subjective cognitive decline.

Quality assessment using the CASP tool indicated studies were generally of moderate to high quality (Table 2, see S3 File for detailed assessment information). However, a small number of studies did not include a clear statement of study aims (n = 3), or enough information to appraise elements of study design (n = 3) or data analysis methods (n = 3). Few studies addressed data saturation issues and most did not adequately address ethical issues or reflexivity.

**Table 2:** Quality appraisal for included studies.

Critical Appraisal of Included Studies (CASP Items)											
Study	A: Validity of Study Results						B: Results			C: Local Value	Overall Rating
	Clear aims?	Qual methods suitable?	Design?	Recruitment?	Data collection?	Reflexivity?	Ethical issues?	Rigorous data analysis?	Clear findings?	Research of value?	
*Arias et al*., *2015*	Yes	Yes	Yes	Yes	Yes	No	Yes	Yes	Yes	Yes	Minor concern
	High	High	Med	High	High	Low	High	Med	Med	High	
*Bardach et al*., *2019*	Yes	Yes	Yes	Yes	Yes	No	Yes	Yes	Yes	Yes	Minor concern
	High	High	High	High	High	Low	Med	Med	High	Med	
*Coley et al*., *2019*	Yes	Yes	Yes	Yes	Yes	Yes	Yes	Yes	Yes	Yes	No concern
	High	High	High	High	High	Med	High	High	High	High	
*Corner et al*., *2004*	No	Yes	Yes	Yes	Yes	No	No	Yes	Yes	Yes	Mod concern
	Low	High	High	High	High	Low	Low	Med	Med	Med	
*Croff et al*., *2019*	Yes	Yes	Yes	Yes	Yes	No	Yes	Yes	Yes	Yes	Minor concern
	High	High	High	High	Med	Low	Med	High	Med	Med	
*Eisenhauer et al*., *2014*	Yes	Yes	Yes	Yes	Yes	Yes	Yes	Yes	Yes	Yes	No concern
	High	High	High	High	High	High	High	High	High	High	
*Etnier et al*., *2017*	Yes	Yes	Yes	Yes	Yes	Yes	Yes	Yes	Yes	Yes	Minor concern
	High	High	High	High	Med	High	Med	Med	High	Med	
*Fogarty et al*., *2014*	No	Yes	No	Yes	Yes	No	No	No	Yes	Yes	Major concern
	Low	Med	Low	Med	Med	Low	Low	Low	Med	Med	
*Friedman et al*., *2009*	Yes	Yes	Yes	Yes	Yes	No	Yes	Yes	Yes	Yes	Very minor concern
	High	High	High	High	Med	Low	Med	High	High	High	
*Friedman et al*., *2011*	Yes	Yes	Yes	Yes	Yes	No	Yes	Yes	Yes	Yes	Very minor concern
	High	High	High	High	Med	Low	Med	High	High	High	
*Grill et al*., *2018*	Yes	Yes	Yes	Yes	Yes	No	Yes	Yes	Yes	Yes	Very minor concern
	High	High	High	High	High	Low	Med	High	High	High	
*Haesner et al*., *2015*	Yes	Yes	Yes	Yes	Yes	No	Yes	Yes	Yes	Yes	Minor concern
	High	High	High	Med	High	Low	Med	Med	High	High	
*Hassan et al*., *2018*	Yes	Yes	Yes	Yes	Yes	No	Yes	Yes	Yes	Yes	Minor concern
	High	High	High	High	High	Low	High	Med	High	High	
*Hulko et al*., *2010*	Yes	Yes	Yes	Yes	Yes	Yes	Yes	Yes	Yes	Yes	Very minor concern
	High	High	High	Med	High	Med	High	High	High	High	
*Hurley et al*., *2005*	Yes	Yes	Yes	Yes	Yes	No	Yes	Yes	Yes	Yes	Very minor concern
	High	High	High	High	High	Low	High	High	High	Med	
*Joosten-Weyn Banningh et al*., *2008*	Yes	Yes	Yes	Yes	Yes	No	Yes	Yes	Yes	Yes	Very minor concern
	High	High	High	High	High	Low	Med	High	High	Med	
*Kim et al*., *2015*	Yes	Yes	Yes	Yes	Yes	Yes	Yes	Yes	Yes	Yes	Very minor concern
	High	High	High	High	High	Med	Med	High	High	High	
*Kim et al*., *2016*	Yes	Yes	Yes	Yes	Yes	Yes	Yes	Yes	Yes	Yes	No concern
	High	High	High	High	High	Med	Med	High	High	Med	
*Laditka et al*., *2011*	Yes	Yes	Yes	Yes	Yes	No	Yes	Yes	Yes	Yes	Very minor concern
	High	High	High	High	Med	Low	Med	High	High	High	
*Lawrence et al*., *2013*	Yes	Yes	Yes	Yes	Yes	No	Yes	Yes	Yes	Yes	Very minor concern
	High	High	High	High	High	Low	Med	High	High	Med	
*Ligthart et al*., *2015*	Yes	Yes	Yes	Yes	Yes	Yes	Yes	Yes	Yes	Yes	Very minor concern
	High	High	High	High	Med	Med	Med	High	High	High	
*Lock et al*., *2007*	Yes	Yes	Yes	Yes	Yes	No	No	No	No	Yes	Major concern
	Med	High	Med	Med	Med	Low	Low	Low	Low	Med	
*Marcum et al*., *2019a*	Yes	Yes	Yes	Yes	Yes	No	Yes	Yes	Yes	Yes	Mod concern
	High	High	High	Med	Med	Low	Med	High	High	Med	
*Marcum et al*., *2019b*	Yes	Yes	Yes	Yes	Yes	No	Yes	Yes	Yes	Yes	Mod concern
	High	High	High	Med	Med	Low	Med	High	High	High	
*Mattos et al*., *2019*	Yes	Yes	Yes	Yes	Yes	Yes	Yes	Yes	Yes	Yes	No concern
	High	High	High	High	High	High	Med	High	High	High	
*Milne et al*., *2018a*	Yes	Yes	Yes	Yes	Yes	Yes	Yes	Yes	Yes	Yes	Very minor concern
	High	High	High	High	Med	Med	Med	High	High	High	
*Milne et al*., *2018b*	Yes	Yes	Yes	Yes	Yes	Yes	Yes	Yes	Yes	Yes	Very minor concern
	High	High	High	High	Med	Med	Med	High	High	Med	
*Nelis et al*., *2018*	Yes	Yes	Yes	Yes	Yes	No	Yes	Yes	Yes	Yes	Minor concern
	High	High	High	High	High	Low	Med	High	High	High	
*Neville et al*., *2013*	Yes	Yes	Yes	Yes	Yes	No	Yes	Yes	Yes	Yes	Minor concern
	High	High	High	High	High	Low	Med	High	High	High	
*O’Brien et al*., *2013*	Yes	Yes	Yes	No	No	No	Yes	Yes	Yes	Yes	Major concern
	High	Med	High	Low	Low	Low	Med	Med	Med	Medium	
*Pace et al*., *2019*	Yes	Yes	Yes	Yes	Yes	No	Yes	Yes	Yes	Yes	Minor concern
	High	High	Med	Med	High	Low	Med	High	Med	Med	
*Price et al*., *2011*	Yes	Yes	Yes	Yes	Yes	No	Yes	Yes	Yes	Yes	Very minor concern
	High	High	High	High	Med	Low	Med	High	High	Med	
*Robinson et al*., *2018*	Yes	Yes	Yes	Yes	Yes	Yes	Yes	Yes	Yes	Yes	Minor concern
	High	High	High	Med	Med	Med	Med	High	High	Med	
*Thogersen-Ntoumani et al*., *2018*	Yes	Yes	Yes	Yes	Yes	Yes	Yes	Yes	Yes	Yes	No concern
	High	High	Med	High	High	Med	Med	High	High	Med	
*Traphagan*, *1998*	Yes	Yes	Yes	Yes	Yes	Yes	No	No	Yes	Yes	Major concern
	High	High	Med	Low	Med	Med	Low	Low	Med	Med	
*Walker et al*., *2014*	Yes	Yes	Yes	Yes	Yes	No	Yes	Yes	Yes	Yes	Minor concern
	High	High	High	High	Med	Low	Med	Med	High	High	
*Watson et al*., *2019*	Yes	Yes	Yes	Yes	Yes	No	Yes	Yes	Yes	Yes	No concern
	High	High	Med	High	High	Low	High	High	Med	Med	
*Wiese et al*., *2018*	Yes	Yes	Yes	Yes	Yes	Yes	Yes	Yes	Yes	Yes	Minor concern
	High	High	High	High	High	High	High	Med	Med	Med	
*Wilcox et al*., *2009*	Yes	Yes	Yes	Yes	Yes	No	Yes	Yes	Yes	Yes	Very minor concern
	High	High	High	High	Med	Low	Med	High	Med	High	
*Wu et al*., *2009*	Yes	Yes	Yes	Yes	Yes	No	Yes	Yes	Yes	Yes	Minor concern
	High	High	High	High	Med	Low	Med	Med	Med	Med	
*Zallen*, *2018*	Yes	Yes	Yes	Yes	Yes	No	Yes	Yes	Yes	Yes	Mod concern
	High	High	Med	Med	Med	Low	Med	Med	Med	High	
***Additional Studies from Updated Searches***											
*Akenine et al*., *2020*	Yes	Yes	Yes	Yes	Yes	No	Yes	Yes	Yes	Yes	No concern
	High	High	High	High	High	Low	Med	High	High	High	
*Bacsu et al*., *2020*	Yes	Yes	Yes	Yes	Yes	Yes	Yes	Yes	Yes	Yes	Very minor concern
	High	High	High	High	High	Med	Med	High	High	High	
*Bosco et al*., *2020*	Yes	Yes	Yes	Yes	Yes	No	Yes	Yes	Yes	Yes	Very minor concern
	High	High	High	High	High	Low	Med	High	High	High	
*Cooper et al*., *2021*	Yes	Yes	Yes	Yes	Yes	Yes	Yes	Yes	Yes	Yes	No concern
	High	High	High	High	High	High	High	High	High	High	
*Halloway et al*., *2020*	Yes	Yes	Yes	Yes	Yes	No	Yes	Yes	Yes	Yes	Very minor concern
	High	High	High	High	High	Low	Med	High	High	High	
*Largent et al*., *2020*	Yes	Yes	Yes	Yes	Yes	No	Yes	Yes	Yes	Yes	Very minor concern
	High	High	High	High	High	Low	Med	Med	High	High	
*McGrattan et al*., *2021*	Yes	Yes	Yes	Yes	Yes	No	Yes	Yes	Yes	Yes	Minor concern
	High	High	High	High	Med	Low	Med	Med	High	High	
*Swindells et al*., *2020*	Yes	Yes	Yes	Yes	Yes	No	Yes	Yes	Yes	Yes	Very minor concern
	High	High	High	High	High	Low	High	High	High	High	
*Wesselman et al*., *2020*	Yes	Yes	Yes	Yes	Yes	No	Yes	Yes	Yes	Yes	Minor concern
	High	High	High	High	Med	Low	Med	Med	High	High	

Yes/No refers to whether each domain was reported and high/medium (med)/low rating refers to extent to which CASP criteria for each domain were met.

Studies from updated searches contributed perspectives from a further 890 individuals (S1 Table). There were no new study contexts. However, in keeping with increased research attention to dementia risk reduction and its implementation, perspectives regarding dementia risk reduction were a more central area of focus for several additional studies [84–86, 91]. Quality of newer studies also appeared higher, although limited reflexivity and ethical consideration remained prominent (Table 2 and S3 File)

### Findings

We identified 17 descriptive themes in our original analysis, with high or moderate confidence in most (Table 3 for overview and S3 File for detailed GRADE-CERQual assessment). From these, we generated six overarching analytical themes that propose key concepts for mechanisms underpinning dementia risk reduction behaviour patterns in the general population and novel ideas for approaches to intervention. An overview of the full framework, with contributing studies and GRADE-CERQual rating for each theme is provided in Table 3 and overarching themes are summarised below. These are interpretive, both encompassing and going beyond the contributing studies (S2 File for more detail of descriptive themes). Additional studies generally corroborated or increased confidence in original findings and some provided additional insights for some themes. No additional themes or refutational concepts were identified. Contributions from additional studies are shown in Table 3 and integrated into the summary of each overarching theme, below. (S2 File for details of themes corroborated or extended by each additional study from search update and S2 Table for all contributing data from all included studies). As carers of people living with dementia, people with a family history of dementia and those living with mild cognitive impairment (MCI) or subjective cognitive decline (SCD) have very different experiences to the general population, differences in emergent themes for these groups are highlighted.

**Table 3:** Overview of thematic framework; contributing studies; confidence assessment.

Analytical Theme	Contributing Descriptive Themes	Contributing Studies in Original Analysis	CER-Qual Confidence Rating for descriptive theme*	Corroborating and Extending Studies in Search Updates
The need for effective education about a complex topic to prevent confusion and facilitate understanding and empowerment	Understanding dementia risk reduction	[43–46, 49, 51–53, 55, 57, 59–61, 64–67, 71, 73, 74, 76, 77, 80–82]	**High.** Minor concerns: methods; relevance. No or very minor concerns: coherence; adequacy	**Corroborating studies:** [84, 85, 88–92]
				**Extending studies:** [84, 91]
		**25 studies**		
	Need for information	[50–52, 55, 57, 59, 60, 62, 63, 65, 66, 68, 69, 71, 74, 75, 78, 80]	**High.** Minor concerns: methods; relevance. No or very minor concerns: coherence; adequacy.	
		**18 studies**		
	Education empowers choice and behaviour change	[43, 46, 48, 51, 52, 54–58, 60, 63, 64, 68–71, 74, 75, 78, 83]	**High.** Minor concerns: methods; relevance. No or very minor concerns: coherence; adequacy.	
		**21 studies**		
Personally relevant short- and long-term benefits of dementia risk reduction behaviour patterns can generate value and facilitate action	The value of reducing dementia risk	[45–47, 49, 50, 52, 58–60, 62–64, 67, 69, 74–80]	**High.** Minor concerns: methods; relevance. No or very minor concerns: coherence; adequacy.	**Corroborating studies:** [85–92]
				**Extending studies:** [86]
		**21 studies**		
	The plausibility and effectiveness of dementia risk reduction	[43, 45–47, 49, 52, 53, 55, 56, 59–61, 63–67, 69, 72–74, 76–82]	**High.** Minor concerns: methods; relevance. No or very minor concerns: coherence; adequacy.	
		**28 studies**		
	The value contribution of other benefits	[45, 46, 48, 50, 52, 55, 56, 63, 67, 70, 72–74, 76, 78, 79]	**Moderate.** Minor concerns: methods; relevance; coherence. No or very minor concerns: adequacy.	
		**16 studies**		
	Weighing costs of dementia risk reduction	[45, 49, 52, 60, 62, 64, 65, 70–72, 76, 78–80, 82]	**Moderate.** Moderate concerns: methods. Minor concerns: relevance. No or very minor concerns: adequacy.	
		**15 studies**		
Individuals benefit from trusted, reliable and sensitive support to convert understanding to personal commitment to relevant behaviour change.	Trusting sources	[46, 52, 55, 59–61, 63, 66, 71, 74, 75, 80, 82, 83]	**High.** Minor concerns: methods; relevance. No or very minor concerns: coherence; adequacy.	**Corroborating studies:** [84, 86, 87, 89, 91, 92]
				**Extending studies:** [84, 88]
		**14 studies**		
	Seeking certainty	[52, 60, 61, 64, 65, 74–76, 79]	**High.** Minor concerns: methods; relevance. No or very minor concerns: adequacy.	
		**11 studies**		
	Avoidance	[59, 71, 74–76, 79]	**Low.** Moderate concerns: coherence; adequacy. Minor concerns: relevance. No or very minor concerns: methods	
		**6 studies**		
Choice, control and relevant self-regulatory supports help individuals take-action and direct their own progress	The impact of intervention characteristics	[46, 48–53, 55, 56, 61, 63, 65, 66, 70–75, 78]	**Moderate.** Moderate concerns: coherence. Minor concerns: methods; adequacy; relevance.	**Corroborating studies:** [84, 85, 87, 88, 90–92]
		**19 studies**		**Extending studies:** [88]
	The importance of personal will	[45, 52, 55, 63, 64, 67, 70, 71, 78]	**Low.** Moderate concerns: coherence; adequacy. Minor concerns: relevance. No or very minor concerns: methods.	
		**9 studies**		
	Reciprocity between self-efficacy and behaviour	[46, 48, 51, 56, 67, 70–74, 78]	**Moderate.** Moderate concerns: coherence. Minor concerns: relevance; adequacy; methods.	
		**11 studies**		
Collaborative and empowering social opportunities can facilitate and propagate dementia risk reduction behaviour change	Social expectations	[46, 50–53, 56, 60, 70, 72–77]	**High.** Minor concerns: methods; relevance. No or very minor concerns: adequacy; coherence.	**Corroborating studies:** [85, 87, 91, 92]
		**14 studies**		
				**Extending studies:** [85, 87]
	Delivery of interventions	[52, 55, 63, 70–72, 75]	**Moderate.** Moderate concerns: adequacy. Minor concerns: methods; relevance. No or very minor concerns: coherence.	
		**7 studies**		
	The importance of peer support and examples	[48, 50, 52, 55, 56, 60, 74–76, 78]	**Moderate.** Moderate concerns: adequacy. Minor concerns: methods; relevance. No or very minor concerns: coherence.	
		**10 studies**		
Individual behaviour patterns occur in social contexts that influence beliefs through heuristic processes and need to be understood	Personal experience	[45–50, 53, 56, 57, 59–61, 63–65, 69, 71–74, 76–82]	**High.** Minor concerns: methods; relevance. No or very minor concerns: coherence; adequacy.	**Corroborating studies:** [84, 85, 91]
				**Extending studies:** [84]
		**27 studies**		

CERQual domain definitions: methods, methodological quality of included studies; relevance, extent to which primary studies supporting the finding are applicable to the context of the review question; coherence, extent to which the finding reflects patterns in contributing data; adequacy, richness and quantity of supporting data.

*, assessment of confidence in theme from original analysis.

#### The need for effective education about a complex topic to prevent confusion and facilitate understanding and empowerment

Forty-six studies contributed to this theme. There was broad awareness of the concept of dementia risk reduction, but the knowledge required to facilitate related behaviour change is more complex and was often lacking. Knowledge gaps and misunderstandings commonly related to the overall concept, behaviours involved, specific behavioural requirements and personal risk-status. These could compound each other and were barriers to behaviour change. Effective, relevant education was commonly sought and is clearly needed. For some, this could directly facilitate adoption of relevant behaviour patterns. It could also empower others to make an informed choice after incorporating additional personal beliefs and considerations.

Many studies highlighted lack of knowledge [52, 57, 60, 65–67, 71, 74, 81, 84–86, 88, 89, 91, 92] and some described it as “the main barrier for behavioural change” [60], including more recent studies [67, 84, 86]. Participants reported awareness of the general concept of dementia risk reduction [51, 52, 55, 59, 60, 66, 68, 71, 74, 80], but described a variety of other, more subtle knowledge gaps, including for: conceptual knowledge [51, 52, 60, 66, 71, 74, 81, 86, 88, 92]; specific theories of dementia risk reduction [45, 49, 51–53, 57, 59, 60, 64–67, 71, 74, 80, 81, 84, 85, 89, 91]; and procedural knowledge for when and how to effectively operationalise general dementia risk reduction understanding in specific behaviour patterns [50–52, 57, 59, 62, 63, 66, 68, 69, 71, 74, 78, 84, 86, 88].

Individual theories of how to reduce dementia risk were limited and largely restricted to combinations of staying cognitively active [43–45, 52, 53, 55, 57, 60, 64, 66, 67, 73, 76, 81, 82, 85, 89]; staying socially active [45, 49, 53, 59, 66, 73, 77, 85, 89]; staying physically active [43, 52, 53, 66, 67, 74, 76, 77, 80, 81, 82, 85, 89, 91]; eating well [43, 45, 49, 52, 53, 57, 67, 71, 73, 80–82, 89, 91]; or generic healthy lifestyles [43, 45, 46, 49, 52, 53, 57, 61, 64, 67, 73, 74, 80–82, 85], such as one study reporting “references to ‘keeping busy’ or ‘staying active’” [81]. In the absence of specific knowledge, some participants directly transferred understanding of behaviours benefiting other physical or mental health conditions [51, 60, 65, 71, 73, 74, 84–86, 91], or behaviours that generate feelings of “a clear mind” [45, 81]. Similarly, many participants reported knowledge about interactions between genetic and lifestyle risk but they often assumed simple genetic inheritance [59, 68, 69, 77, 80, 83, 91]. For example, one participant reported he “gave up trying to prevent it due to my family history” (despite no evidence of ‘familial’ dementia) [59].

Participant descriptions of specific behavioural requirements often suggested influence from personal or cultural identity, lifestyles and values [45, 49, 51–53, 57, 59, 60, 65–67, 71, 74, 81, 85]. Prominent examples included ascribing a protective value to “home-made and home-grown foods” [45], “exercising the spirit” [57] or “rural lifestyles” [49, 51, 52, 60, 66, 67, 71, 74, 81]. Some believed that they were already living “healthy lifestyles” that would reduce dementia risk and consequently did not need dementia risk reduction information because they “knew it already”. Such discrepancies between perceived and actual knowledge could preclude effective behaviour change [46, 71, 86].

More prominently, individuals sought education to address acknowledged knowledge gaps and generally improve understanding [43, 46, 48, 51, 52, 54–58, 60, 62, 68–71, 74, 75, 78, 83–86, 88–91]. While beliefs about whether education alone could facilitate behaviour change varied [48, 51, 52, 54–57, 60, 68, 70, 71, 74, 75, 78, 83], many participants found it empowering: it was described as a “main motivator”, “helpful” or “useful” [60] for either changing behaviour or for making more informed, autonomous choices based on personal circumstances [56, 68, 69, 71, 75, 84, 92]. For example, one participant noted “you can choose to ignore it, but you’ve been given the information and it’s your choice what you do with it, whereas at the minute it’s not there” [75]. Of note, carers of people already living with MCI or SCD were more pessimistic regarding the benefits of education [45, 55, 59, 66].

#### Personally relevant short- and long-term benefits of dementia risk reduction behaviour patterns can generate value and facilitate action

Forty-six studies contributed to this theme. Lower dementia risk was generally valued, where it was accepted it as plausible. However, the overall dementia risk reduction value proposition was strongly influenced by other perceived benefits and costs for personal priorities for quality of life in the short- and longer-term and, for some, social and community benefits. Such potentially complex evaluations were key to uptake motivation and to motivation to maintain behaviour change over time. Broader benefits were not always anticipated, at least prior to change initiation. Highlighting quality-of-life-relevant short- and long-term benefits of both dementia risk reduction change and dementia risk reduction interventions could enhance their attractiveness.

Dementia was highly feared across studies and lower dementia risk was correspondingly valued highly, particularly in carers, participants with MCI/SCD and people with a family history of dementia [44, 50, 58–60, 62, 67, 69, 74, 83]. Participant accounts attributed value to preserving “what matters in everyday life” [79], including optimising general function, quality of life, relationships, identity and independence; and minimising burdens on families and society [45, 46, 59, 67, 76, 77, 79, 84, 87]. A few participants, however, failed to value lower dementia risk [47, 63, 64, 74, 84, 89] and some studies linked this to “a reduced sense of value for older citizens in society and dulled expectations” [47, 89].

Participant valuations of lower dementia risk were often closely linked to stances regarding the plausibility and effectiveness of adopting dementia risk reduction behaviour patterns [45–47, 55, 64, 66, 69, 74, 75, 81, 84]. Beliefs that dementia risk reduction related behaviour patterns were “important” [67] were “frequent” or “striking” in some studies [45, 80], although participants commonly and mistakenly assumed dementia risk reduction would ensure ‘prevention’: “if you take care of yourself, you are going to be okay, and if you don’t, you won’t” [80]. Participants were less emphatic in valuing lower dementia risk when aware that outcomes were probability-based, such as those who “voiced concern that efforts to reduce risk might be ineffective” [61, 84]. In contrast, for participants with MCI/SCD, “any chance of improvement or delay in deterioration seemed to merit participation” [62]. Some participants did still endorse persisting myths that dementia is “normal” or “expected” [47] with ageing [47, 59–61, 66, 69, 74, 77, 80], or is inevitable in the setting of any family history [59, 64, 80, 84]. However, others described dementia prevention as plausible despite significant genetic risk [55, 59, 64, 65, 69, 78, 83, 86]. The latter beliefs could strongly facilitate action, such as one participant reporting that awareness of her significant family history of dementia meant that “what we are doing, it is as if I had already been told that I have Alzheimer’s, so I am already in the field” [69].

Many participants emphasised outcomes other than lower dementia risk in their valuation of dementia risk reduction behaviour. According to participant accounts, valued short- and long-term outcomes included: quality of life [46, 79, 86, 87], mental and physical wellbeing [45, 46, 48, 50, 56, 63, 67, 70, 72–74, 76, 85, 86, 90], enjoyment or interest [46, 48, 52, 55, 74, 78, 79, 85, 89] and opportunities for social engagement [50, 52, 56, 76, 87]. Studies highlighted these as an “important measure of effectiveness” [79] or “empowering” [70]. Some studies also noted that benefits for communities or society carried “greater motivational weight” [48, 74]. Despite their perceived value, broader short-term benefits were sometimes not-anticipated [48, 56], contributing to recommendations that education should not “focus specifically on dementia” [75].

Finally, some participants weighed up important costs to their short- and long-term priorities against perceived benefits of dementia risk reduction behaviour patterns for overall attitudes and intentions, regardless of beliefs regarding effectiveness [45, 49, 52, 60, 62, 64, 66, 70–72, 76, 78–80, 82]. Consistent with an overall quality of life value-proposition, costs to competing family and occupational roles, including creating burdens for family [45, 49, 52, 60, 64, 70–72, 84, 87], loss of pleasure [60, 80, 82], medication side-effects [62, 66, 76, 78, 79] and costs to other medical condition management [86] were all potential barriers to behaviour change, as seen in one participant’s concern that she had “too much in my life to devote to it that much” [70].

#### Individuals benefit from trusted, reliable and sensitive support to convert understanding to personal commitment to relevant behaviour patterns

Twenty-six studies contributed to this theme. Perceiving dementia risk reduction as personally relevant and legitimate was emphasised as important for converting general beliefs into specific behavioural intentions. Some evident barriers to this are inherent to dementia risk reduction, such as gaps in supporting evidence and the association with a feared and stigmatised condition. However, enablers for individuals to navigate these could include: using known and trusted formal and informal sources to deliver information; ensuring that information includes cautious, accurate, positive messaging; and complementing information with support for individuals to interpret it and accept the anxiety/stigma of identifying as ‘at-risk’. These factors should be incorporated across intervention types.

Some participants described gaps between dementia risk reduction knowledge and either their intended behaviours or their beliefs that it applied them as individuals [60, 61, 64, 65, 68, 69, 75, 76, 82]. Their accounts linked gaps to scepticism regarding advice [60, 65, 68, 69, 82, 84] and reluctance to identify as ‘in need’ of dementia risk reduction [64, 74–76, 79, 86].

Participants tended to be less sceptical about advice or interventions that they considered legitimate, based on how “trustworthy” [55] and “reliable” [46] they considered its source [46, 55, 63, 71, 74]. Social networks (especially friends/family with lived experience) [52, 63, 66, 74, 82] or experts with ‘trusted’ reputations were preferred, and distrust of experts was rare [60, 84]. This was illustrated by the comment: “when the wise people say ‘it’s better for you’, well, they know better than me” [63]. Healthcare professionals [46, 52, 55, 61, 63, 66, 71, 74, 75, 84, 86], churches [55], universities [46, 74], government bodies [46, 74] and dementia-related non-governmental organisations (NGOs) [74, 82] were all cited as trusted experts that could promote buy-in [71, 84], by providing a “confidence building measure” [55]. Some studies and participants also highlighted a complementary role for “peer education … within social networks” to broadly disseminate a trustworthy message [52, 66, 74, 82, 84, 87].

In contrast, shifting advice [52, 75, 81], “unclear” or inherently uncertain outcomes [60, 61, 64, 68, 74, 75, 82] and gaps and inconsistencies in evidence [60, 61, 66, 68, 74, 75, 81, 82, 84, 86, 89, 91, 92] generated scepticism for many participants. While some reports suggested trusted sources, particularly personally known health professionals, could mitigate scepticism [63, 66, 71, 84], unclear or inconsistent advice could also diminish trust in expert sources [60, 75, 81, 82] and, thereby, any sense of urgency regarding behaviour change [60, 64, 65, 68, 75, 82]. This was exemplified by one participant’s assertion that “you need to prove to us that something works first” [82]. Some studies linked scepticism to limited knowledge [59, 62, 74, 75, 79]. Other participants felt messaging that appeared overstated could exacerbate scepticism and recommended that education use cautious terms such as “may” [75], combined with support to interpret information [63, 65, 66, 71, 75, 84].

Some participant accounts linked reluctance to acknowledge the personal relevance of dementia risk reduction to fear of dementia [71, 75, 79, 84, 89], and stigma regarding ageing [75, 76]. Descriptions indicated both could generate “considerable anxiety” [71, 75, 84] and some participants preferred to “carry on in blissful ignorance” [79] or to avoid “thinking about” anything associated with dementia in their own lives [74, 76, 79, 84, 89]. Some reports indicated that avoidance could be magnified by knowledge gaps, endorsing myths that dementia is ‘normal’, and scepticism regarding advice or evidence [84, 89]. Others highlighted that anxiety and avoidance could be triggered by fear messages or interventions focusing on ‘risk’, rather than ‘risk reduction’ [74, 75, 79, 87], such as one participant questioning: “why scare them? You’re trying to get them to move” [74, 75].

#### Choice, control and access to relevant self-regulatory supports help individuals take-action and direct their own progress

Twenty-eight studies contributed to this theme. Choice and feeling autonomous or in control of decisions was important throughout individual dementia risk reduction journeys. Similarly, tailoring interventions to individual preferences and circumstances across factors such as content, delivery, timing and sequence could help individuals to enact intended behaviour change. These highlighted the importance of collaboration and user-participation throughout intervention design and delivery processes. Individual accounts also highlighted diverse deficiencies in self-regulation capacities and strong benefit from interventions supporting and developing these. Ensuring individuals can actively participate in designing interventions that include personally relevant supports for self-regulation could further enhance motivation and behaviour change through individuals directing their own dementia risk reduction journey to best meet their needs.

Participants emphasised diverse intervention preferences and the importance of choices, including for when and how information is delivered, intervention platform, appearance or “aesthetics”, content, timing, sequence and diverse other characteristics [46, 48–53, 55, 56, 63, 65, 66, 70–75, 78]. In some accounts, behaviour change was strongly enabled by participants being able to choose intervention components to accommodate personal circumstances, including physical capacity, skills and values [46, 48–50, 52, 55, 56, 63, 65, 70–72, 78, 85, 88, 90–92]. As commented by one participant: “everyone has his own unique way … activities should differ from person to person” [52].

A preference for choice complemented participant preferences to feel involved in designing their own journey and to have their “autonomy respected” [45, 63] in all decisions about if and how to act [46, 48, 50–53, 55, 56, 63, 67, 70–72, 74, 75, 78, 84, 87, 88]. Participant reports highlighted that overly prescriptive interventions were likely to be ineffective and studies also noted that these could cause “resistance” [52, 55, 63]. This was demonstrated by one woman’s reasoning for dropping out of an intervention trial: “‘Always ‘you have to.’ I detest it. They don’t ask you what you want to do about it yourself” [63]. Preferences for ensuring participants “felt heard and respected” [63] held even when there was evidence of “negative attitudes and stubbornness as barriers” [52].

Concurrently, deficient self-regulation skills (or low confidence in these skills) were prominent in several studies. Participants described these skills (or deficiencies), such as “self-initiative” [67], as influencing whether they acted on behavioural intentions [45, 48, 52, 55, 56, 63, 64, 67, 70–72, 74, 75, 84, 90]. They were often aware of deficiencies and sought access to relevant supports, such as one participant who felt access to feedback was important “to see where I stand, what I am able to do, what is required from me” [55]. Help to anticipate and monitor the target behaviours and their benefits [52, 55, 56, 71, 72, 75], or to effectively plan activities and navigate barriers [48, 52, 53, 55, 56, 63, 70–75], were commonly described as useful. However, many individuals also disliked self-regulatory supports when they didn’t feel they were needed [56, 63, 70]. Again, this highlighted the need for individuals to direct the supports they use.

Self-regulation support could be particularly important for individuals with low self-efficacy. Participant accounts emphasised that existing cognitive problems, physical comorbidities or disabilities, and deficient technical skills (particularly for technology-based interventions) all diminished self-efficacy. This often specifically included low confidence in self-regulatory capacities [46, 48, 55, 56, 67, 70–73, 90]. However, some participants reported that access to supports for regulatory skills without feeling pressured into something they felt they were not capable of improved “confidence” and helped them feel they were “taking charge” [48, 51, 56, 70, 72, 78].

#### Collaborative and empowering social opportunities can facilitate and propagate dementia risk reduction behaviour change

Twenty-three studies contributed to this theme. Social factors were key drivers of dementia risk reduction behaviour patterns. Opportunities for social interactions with both peers and health professionals that are collaborative and supportive and that engender a sense of social obligation could directly facilitate dementia risk reduction behaviour change. These influences could bypass knowledge gaps or pessimism regarding effectiveness. Through peer-support, particularly encouragement and setting positive examples, individuals themselves could then facilitate further change in their social networks. Interventions that generate and leverage supportive social processes could optimise dementia risk reduction intervention effectiveness and reach.

Studies highlighted that anticipating opportunities for enjoyed and valued social interaction [46, 50, 51, 53, 56, 72–74, 76, 87, 90, 92], and a sense of social obligation [50, 60, 70, 74, 75, 77] could directly facilitate both adoption and maintenance of relevant behaviour patterns, regardless of knowledge or attitudes and, for some, self-regulation deficiencies [45, 48, 52, 70, 76]. For example, one study noted that “the majority of participants explained that a dance class could provide an important opportunity for social interaction and that this would be an integral facilitator as well as a valued benefit” [76]. Participant accounts also emphasised that opportunities for enjoyable social interaction and feeling accountable to friends, formal intervention ‘buddies’ or healthcare professionals influenced behaviour [46, 50, 60, 70, 74, 75, 77]. Some participants clarified the latter to relate it to either an internal wish to be “dependable” or to external measures such as regular check-ins with professionals [45, 46, 48, 50, 52, 76].

Participants across diverse studies also sought similar general characteristics in social interactions. They tended to evaluate interactions with peers [48, 50, 52, 74, 76, 78, 87], or with healthcare professionals and other research or public health professionals [52, 53, 55, 63, 70, 71, 75, 88] as helpful when they were positive and collaborative, respectful, considerate of individual circumstances and reciprocal. For example, one participant described appreciating behaviour change sessions during an intervention trial because they were “not advice but more like a conversation” [63]. In contrast, “directive and moral” or “patronising” [63] interactions were seen as unhelpful and could contribute to intervention drop-out [55, 63, 72].

Direct peer support, where social interactions involved participants both receiving and providing helpful peer comparisons and “encouragement” [50] were also seen as empowering and change promoting [48, 50, 52, 55, 74, 76, 78], although some cautioned that support should not impede independence [87]. Some participants identified with taking responsibility to provide a good example and support for their peers, such as beliefs that “we can do it, and they can follow” [52, 87]. Some also clarified that this sense of responsibility helped them to feel reassured about their current function and more optimistic about their futures, helping them to maintain health-promoting behaviours [52, 56, 76, 87]. Others suggested more formal support services could then focus on individuals who do not have access for peer- or family-support [60, 75].

#### Individual behaviour patterns occur in social contexts that influence beliefs through heuristic processes and need to be understood

Twenty-nine studies contributed to this theme. Individuals developed heuristic knowledge and beliefs based on their personal contexts, particularly experiences observing family members living with dementia, inherited wisdom from families or communities and culturally-endorsed understandings. These were valued and could act as influential enablers or barriers for behaviour change, including through generating cognitive bias. Understanding how such heuristic knowledge shapes beliefs and motivation is important during intervention design and delivery to ensure that this knowledge is acknowledged and addressed or accommodated to improve intervention effectiveness.

Studies described personal experiences and influences from social or cultural contexts strongly influencing existing theories of dementia risk reduction [45, 49, 53, 57, 59, 71, 73, 77, 80, 81, 84, 85, 91]. Participant descriptions of individual theories often referenced activities they had observed in cognitively healthy older people [45, 53, 57, 59, 71, 77, 80], or associated with historical periods in which they believed dementia was less common [49, 73, 80, 81]. For example, one study described participants holding “shared beliefs about home-made and home-grown food … in prior generations within their families” [45].

Several studies and participant accounts highlighted similar heuristics and cognitive bias influencing dementia risk reduction valuations. Some participants valued dementia risk reduction highly because they had observed people living with dementia [46, 49, 50, 52, 55, 59, 69, 78, 79], such as one participant’s acknowledgement that “I see my completely dependent mother and I am projecting myself” [46]. Others generalised dementia outcomes for people whom they believed led healthy lifestyles [47, 60, 61, 64, 66, 82]. For example, scepticism could be linked to knowing “too many people that did literally everything ‘right’ and still ended up with dementia” [66]. A few participant reports also included ageist assumptions about appropriate intervention types (e.g., considering technology unsuitable for older people) [48, 67, 72, 73], or reflected stigma regarding physical health problems or disability, such as beliefs that minor health problems prevented any physical activity [56, 70, 71].

Some participants referenced these heuristics and experiences more than expert advice [49, 59, 60, 66, 69, 77, 79, 80, 82, 83]. In other accounts, they were integrated with expert advice [64, 73, 80, 81], such as one participant combining knowledge from expert education and from Chinese proverbs to create a personal approach in which she “always emphasises balance … balance” [81]. These processes could leave individuals vulnerable to ineffective behaviour change. Importantly, some studies reported that bias could be amenable to correction through education, and some participants also described this occurring through participating in activities, such as one trial participant noting that his “attitude has changed towards my health problems [following participation in a dementia risk reduction clinical trial]” [56, 64, 70]. However, some studies also noted that individuals with a family history of dementia appeared less likely to correct bias through education [56, 64, 70].

#### Age, gender and country influences

Limited reporting meant detailed analysis of age and gender influences on findings was not possible. Most studies examined the perspectives of middle-aged and older individuals. However, one study including younger participants, reported that this group focused more on smoking cessation and not drinking alcohol in theories of dementia risk reduction [60]. In another study, older participants placed greater emphasis on usability, including easy-to-follow recommendations and easy-to-use intervention tools [48]. Some older participants also suggested that younger people are more likely to de-value later life [75]. Two studies specifically compared the perspectives of men and women. One reported that women perceived dementia as more severe and dementia risk reduction as more important and effective [74]. Both described disseminating information within the family as a key role only for women [74, 82].

We did not identify obvious differences in findings between countries, although this was likely in part because the majority of studies were conducted in a small number of English-speaking HICs. One series of papers from the USA did compare groups with different cultural identities [52, 53, 61, 74, 81]. Two studies specifically examining perspectives of Indigenous peoples in Canada both noted greater emphasis on social and environmental factors as protective [57, 73].

### Summary model of key concepts and intervention recommendations

**Fig 4:**
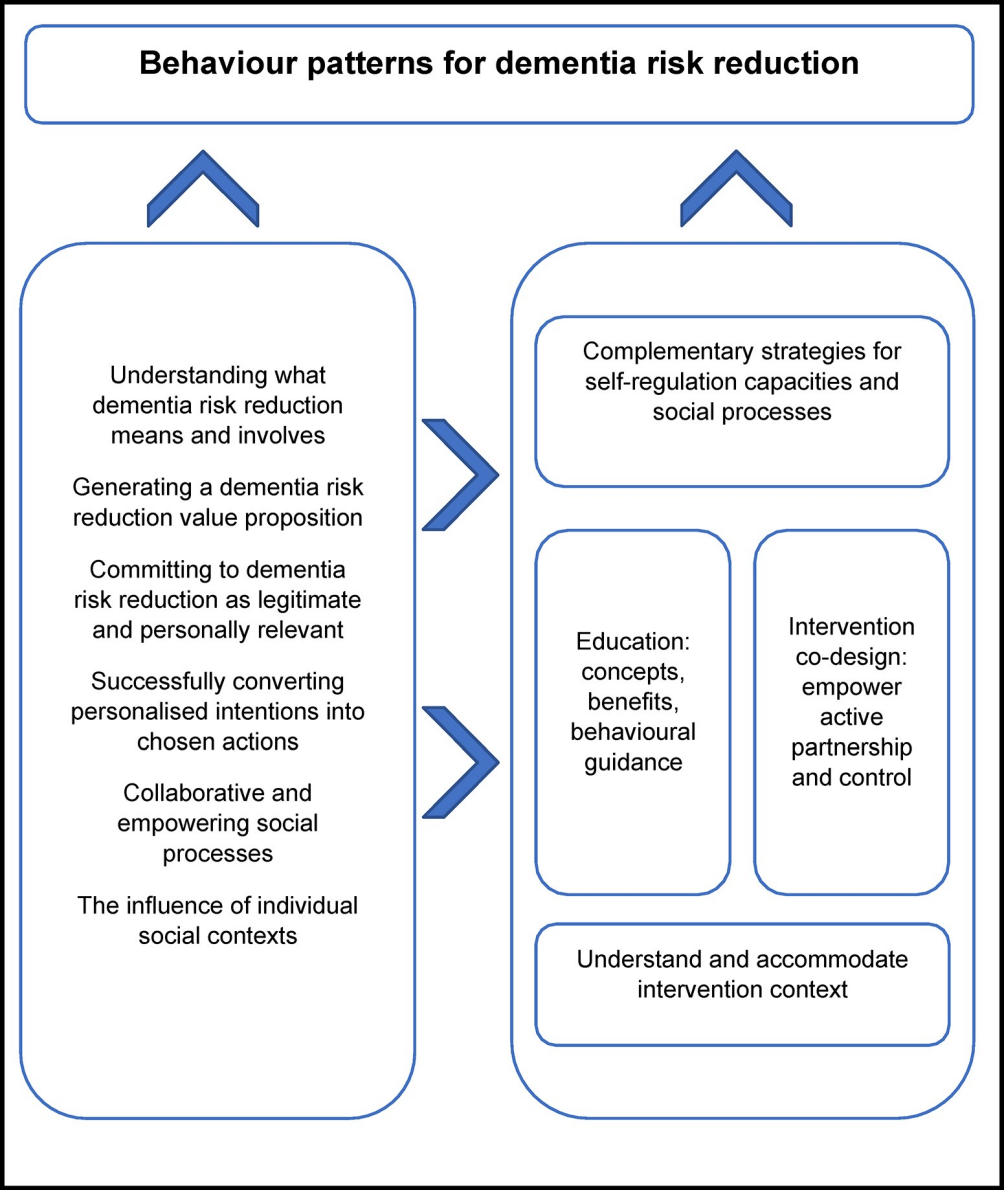
An integrated model of general population dementia risk reduction perspectives and approaches to intervention. The model suggests key considerations for approaching interventions to support individuals to adopt and maintain dementia risk reduction behaviour patterns. Detailed understanding of intervention context should be used to co-design tailor and detail intervention approaches based on this model. Education is key and should be complemented by tailored strategies to: support self-regulation; and develop and leverage supportive social environments, skills and resources. Themes, adapted to context, should inform the specific strategies used and their content.

The expert panel discussion endorsed the synthesis findings as consistent with their experience and reached consensus on how these interpretive themes and ideas could be combined in a practical, integrated summary model that can guide intervention design and delivery processes. The integrated model is shown in Fig 4 and briefly described below. The model incorporates key concepts from the analytical themes, outlining change mechanisms likely underpinning dementia risk reduction behaviour patterns, and how interventions can address these to optimise behaviour change promotion.

The integrated model illustrates that, first, education should be a key component of intervention. Education should be delivered respectfully and include expert and peer involvement or endorsement. It should address the meaning of dementia risk reduction, including complexities such as timing and cumulative exposure, the behavioural patterns involved, and specific behavioural guidance that is made relevant to target population contexts. Education should also employ careful but positive messages about reducing dementia risk and emphasise the other diverse short- and long-term benefits that relevant behaviour patterns offer, including enjoyment and maintaining function and quality of life. This may offset concerns regarding uncertain outcomes and being ‘at risk’.

Second, education should be complemented by intervention components that support effective self-regulation (e.g. anticipating and recognising immediate benefits, planning) and generate supportive social processes (e.g. accessible social support; ‘positive’ social norms). These intervention components can help individuals to act on intentions and sustain behaviours change. They may also optimise intervention outcomes by facilitating spread of both knowledge and behaviour change beyond direct recipients of the intervention.

Third, all aspects of intervention design and delivery planning should be a collaboration with target populations. This can facilitate the understanding of context, sense of choice, autonomy and intervention tailoring that can improve outcomes. These collaborative design processes mirror the principles of intervention co-design: they emphasise a shared purpose and understanding, active partnerships and equal voices for professionals and consumers to facilitate better outcomes. Hence, design and delivery processes should apply these principles. Professionals need to understand, respect and value target populations and influences from their existing contexts. Individuals should direct tailoring processes to suit their preferences and contexts. Resource and other pragmatic limits to consumer choice and control should be acknowledged early, allowing individuals to make informed decisions. To enable genuine collaboration, interventions should include specific efforts to empower target populations and understand their contexts. Finally, this understanding of intervention context should prompt adaptation of this model itself to optimise utility.

## Discussion

### Summary of findings

This systematic review and qualitative synthesis identified a substantial literature examining the perspectives of the general population regarding dementia risk reduction. From 50 papers that included the views of over 4,500 individuals, we identified generally consistent themes across studies and over time. Detailed assessment of contributing studies indicated that we should have moderate to high confidence in the findings. We generated novel insights into how and why individuals adopt and maintain dementia risk reduction behaviour patterns and a practical summary model of key concepts from themes to guide intervention design and delivery.

We highlighted that complex belief systems contribute to adopting and maintaining behaviour patterns that may reduce dementia risk, and that using complementary intervention approaches could facilitate more effective promotion of these behaviour patterns. Comprehensive education should be a key component of intervention. This should generate broad perceived value, use trusted sources and be delivered carefully and respectfully. Education should be complemented by access to tailored supports for self-regulation and positive social processes. Applying co-design principles throughout intervention design and delivery processes should help individuals feel both supported and autonomous, which may better enable changes. Importantly, individual social and historical contexts can strongly influence beliefs and need to be understood and addressed or accommodated to optimise intervention design.

We also identified preliminary findings that carers, people with a family history of dementia, and people already living with SCD or MCI may hold some different perspectives, influenced by their experiences. These findings, as well as the influences of age, gender and cultural identity, should be explored further.

Our findings are consistent with earlier reviews identifying limited knowledge and understanding of dementia risk reduction and strengthen calls for education to address knowledge gaps [27–30]. Findings are also consistent with theoretical models of behaviour change mechanisms and intervention construction as most of these highlight key roles for improving knowledge, motivation, self-regulation capacities, skills, habits and social processes in order to change a range of health behaviours [17, 18, 24, 26]. Our model is also consistent with models developed and employed effectively in other complex health promotion contexts [26]. For example, theory, existing evidence, primary qualitative data and participatory design processes were combined to develop a comprehensive model highlighting complex knowledge, self-regulation capacities, social support, affect and identity as underpinning self-management in type two diabetes [93]. This model has supported effective intervention in subsequent trials [94]. Further, intervention meta-analyses have produced models for complex behaviour change in cardiac rehabilitation [95] and obesity contexts [96]. These also emphasise interventions for self-regulation, social support and education using credible sources (cardiac rehabilitation), and self-regulation and communication styles that generate autonomy (obesity).

To date, however, a model of behaviour change intervention construction with specificity for dementia risk reduction contexts has not been developed. Intervention components in past dementia risk reduction trials have, instead, been based on common sense, generic theory, models from other health contexts or limited empirical data on usability [11–13, 97]. Our findings now provide a more comprehensive model and framework to scaffold development of more tailored and targeted dementia risk reduction interventions [23, 24].

### Strengths and limitations

Our systematic and comprehensive searching identified a relatively large body of evidence that reflected a broad range of contexts. The emergence of consistent themes across diverse contexts and over time adds weight to the review findings. Our methodology, including GRADE-CERQual confidence assessment for each review finding, increases usability for other researchers and public health professionals [32, 35].

Limited representation of people from low- and middle-income countries (LMICs), and of younger people are significant gaps in this literature (including in updated searches). This reduces confidence in the generalisability of some review findings. Excluding studies not published in English may have exacerbated this gap. This is unfortunate as dementia risk reduction may offer greatest public health benefit in LMICs [98], which will bear greater future dementia burden [99] and likely have less access to advances in disease modifying treatments [100, 101]. Similarly, beliefs in younger years can strongly influence behaviour patterns in the critical middle-age period [3, 4, 102]. Further primary research addressing these gaps will support stronger future reviews and model development. These limitations of the current literature notwithstanding, the model developed here shows important correspondences to other intervention-design frameworks [26], bolstering confidence in its applicability.

The types of dementia risk reduction interventions examined varied across included studies. While drawing together a larger number of studies allowed a more integrated model, perspectives may differ for different types of interventions. Similarly, limited sample descriptions in included studies restricted analysis of sub-group perspectives. While some qualitative approaches do not emphasise participant descriptions, more precise description of participants and context in this literature would enable greater insight into differences that warrant tailoring of intervention design and delivery to specific recipients.

Finally, our decision to primarily extract data from only the results/findings sections of included papers may have missed some additional themes. However, this design trade-off helped us to more accurately identify the most prominent and influential underpinning mechanisms for dementia risk reduction behaviour patterns. By checking other paper sections for clearly different findings or additional interpretations, we reduced the likelihood of key data being missed.

### Implications for research and practice

This model has important immediate practical utility in both research and dementia risk reduction implementation activities. As efficacy trial outcomes rely on effective behaviour change, the model can be used to guide intervention design and support improved outcomes. In implementation contexts, clinicians and program designers can use the model to ensure that they understand and are attuned to key areas and complexities when promoting behaviours that contribute to individuals lowering their risk of dementia.

To accelerate progress in dementia risk reduction research, intervention trials should also be explicit about the content of interventions, including strategies used to support adoption and adherence and the rationales underpinning them [103]. The effect of interventions on postulated mechanisms of change and behavioural outcomes should then be investigated in process evaluations and emphasised in outcome reporting [26]. This will help elucidate how and why interventions are or are not effective for health outcomes, facilitate iterative development of models such as the one reported here and prevent costly use of interventions offering little additional value.

Finally, this review has focused on individual level dementia risk reduction implementation. This should be complemented by coordinated multi-level actions, including with clinicians, organisations and governments [101, 104]. The model should be coupled with clear implementation strategies directed at clinicians, and population health interventions. To date, there has been limited examination of multi-level dementia risk reduction implementation needs, but a recent review of primary care practitioners (PCPs) identified important barriers to incorporating dementia risk reduction into practice, such as prioritising more urgent patient needs in the context of time and other resource limitations [105]. Understanding further implementation barriers across socio-ecological levels will be key to ensuring individuals can access and benefit from effective interventions.

## Conclusion

This review provides the first comprehensive synthesis and integrated model of general population perspectives regarding dementia risk reduction that influence adoption and maintenance of relevant behaviour patterns, and a proposal for a tailored intervention approach incorporating co-design principles, education and complementary behaviour change strategies to address specific underpinning mechanisms. Systematically building upon this work by designing and testing specific dementia risk reduction behaviour change interventions in target populations to iteratively develop the model, and by applying a coordinated approach to implementation across socio-ecological levels, will help to answer the WHO’s call for immediate, effective dementia risk reduction implementation.

## Supporting information

S1 File Adapted PRISMA checklist. (DOC)

S2 File Additional methods and findings. (DOCX)

S3 File Detailed quality appraisal and GRADE-CERQual assessment data. (XLSX)

S1 Table Individual study characteristics. (DOCX)

S2 Table All coded quotations and author interpretations. (DOCX)
