# Supplementary material for: General population perspectives of dementia risk reduction and the implications for intervention: A systematic review and thematic synthesis of qualitative evidence
Source: PLoS One. 2021 Sep 17;16(9):e0257540. doi: 10.1371/journal.pone.0257540 (PMC8448319; doi:10.1371/journal.pone.0257540)
Supplement: S2 File — (DOCX) [file pone.0257540.s002.docx]

# **S2 File: Additional Methods and Findings**

Contents

[Additional Methods and Findings 1](#_Toc78403314)

[Aim 1](#_Toc78403315)

[Methods 1](#_Toc78403316)

[Complete search strategy for each database 1](#_Toc78403317)

[Reflexivity 2](#_Toc78403318)

[Results 2](#_Toc78403319)

[Participant characteristics 2](#_Toc78403320)

[Findings 3](#_Toc78403321)

## Aim

A secondary aim of this study was to compare the dementia risk reduction perspectives of people living with depression to those of the general population. However, no extant data for people living with depression was identified and this objective was not subsequently pursued.

## Methods

### Complete search strategy for each database

All electronic database searches were performed on December 18, 2018 and updated on December 18, 2019.

Medline (Ovid):

1. (Alzheimer* or Cogn* decline or Cogn* impair* or dementia or Impair* cogn* or Memory complain* or Memory loss or predementia).mp.

2. (onset or prevent* or protect* or risk*).mp.

3. (attitude* or barrier* or belief* or enable* or experience* or facilitat* or focus group* or interview* or needs or opinion* or perspective* or qualitative or themes or view*).mp.

4. 1 and 2 and 3

5. limit 4 to (English language and humans and yr="1995 -Current")

[mp=title, abstract, original title, name of substance word, subject heading word, keyword heading word, protocol supplementary concept word, rare disease supplementary concept word, unique identifier, synonyms]

PsycINFO (Ovid):

1. (Alzheimer* or Cogn* decline or Cogn* impair* or dementia or Impair* cogn* or Memory complain* or Memory loss or predementia).mp.

2. (onset or prevent* or protect* or risk*).mp.

3. (attitude* or barrier* or belief* or enable* or experience* or facilitat* or focus group* or interview* or needs or opinion* or perspective* or qualitative or themes or view*).mp.

4. 1 and 2 and 3

5. limit 4 to (human and English language and yr="1995 -Current")

[mp=title, abstract, heading word, table of contents, key concepts, original title, tests & measures]

Embase (Ovid):

1. (Alzheimer* or Cogn* decline or Cogn* impair* or dementia or Impair* cogn* or Memory complain* or Memory loss or predementia).ti.

2. (onset or prevent* or protect* or risk*).mp.

3. (attitude* or barrier* or belief* or enable* or experience* or facilitat* or focus group* or interview* or needs or opinion* or perspective* or qualitative or themes or view*).mp.

4. 1 and 2 and 3

5. limit 4 to (human and English language and yr="1995 -Current")

[ti=title]

[mp=title, abstract, heading word, drug trade name, original title, device manufacturer, drug manufacturer, device trade name, keyword, floating subheading word]

CINAHL: 4,223

1. Alzheimer* or "Cogn* decline" or "Cogn* impair*" or dementia or "Impair* cogn*" or "Memory complain*" or "Memory loss" or predementia

2. onset or prevent* or protect* or risk*

3. attitude* or barrier* or belief* or enable* or experience* or facilitat* or “focus group*” or interview* or needs or opinion* or perspective* or qualitative or themes or view*

4. 1 and 2 and 3

5. limit 4 to (human and English Language and published date “19950101-20181231”)

### Reflexivity

We considered how the views, opinions and professional backgrounds of authors may have influenced study design and conduct. EC, TC and NL work in clinical roles as old age psychiatrists in Australia. They all address the consequences of dementia and the lack of treatment options in clinical work. VP All authors have been involved in dementia risk reduction or public health research and believe that the topic is an important public health issue that has not been adequately addressed to date in Australia. We actively sought disconfirming data for these beliefs, and carefully considered supporting data where themes consistent with these beliefs emerged.

## Results

### Participant characteristics

Participant descriptions and discussion of the role of participant characteristics in emergent themes was variable.

39 (95%) studies included some information about sample age and the majority included only middle-aged or older participants. Two studies compared perspectives between age-groups [1, 2].

36 studies (88%) described the gender mix of samples and two studies specifically compared themes between men and women [3, 4].

Some studies reported including specific population sub-groups of interest, particularly carers, people with a family history of dementia and people with subjective cognitive decline (SCD) or mild cognitive impairment (MCI). 17 (41%) studies described including participants who were living with subjective cognitive decline (SCD) or mild cognitive impairment (MCI) or who were carers or biological relatives of people living with dementia. Of these, 7 provided valuable comparisons of themes between two sub-groups or between sub-groups and the general population [5-11].

Diverse study designs were applied, with focus group discussions (FGDs) or individual interviews the most common approaches for data collection. Studies also reflected diverse contexts. 10 studies examined dementia risk reduction perspectives in the context of disclosure of dementia risk status (including pre-clinical biomarker or genetic test results); 13 studies evaluated different types dementia risk reduction interventions; 16 studies focused on views regarding healthy ageing, including aspects of healthy cognitive ageing; and, nine studies focused on views regarding cognitive decline and dementia, including aspects of dementia risk reduction.

17 (41%) eligible studies did not report the cultural or ethnic identity of participants. However, nine studies either focused on the views of Indigenous or ethnic minority populations, or compared views between different cultural groups [2-4, 12-17].

Author orientation and study context influenced methodology, the aspects of the topic covered and author interpretations. Some studies were oriented to examining conceptual beliefs while others focused on engaging in individual dementia risk reduction activities. Some papers linked perspectives more specifically to behavior change for dementia risk reduction. For example, one Australian study specifically examined beliefs comprising enablers or barriers to lifestyle behavior change [1]. Coverage of emergent themes varied between studies and none encompassed the full picture generated by the thematic synthesis. Our model proposes that these perspectives are connected and together comprise a pathway informing decisions about taking up dementia risk reduction behaviors.

### Findings

A detailed summary of descriptive themes is below. The contribution of each additional papers from search updates, including corroborated themes and additional insights is subsequently detailed in Table 4.

***Knowledge and understanding***

The review of literature found that many people were broadly aware of the concept of DRR and understood it to involve lifestyle-related behaviours; particularly physical activity, cognitive activity, social activity and/or diet. Life circumstances and knowledge gaps did, however, lead to diverse interpretations and applications of these behaviours. Attitudes to information and education to address knowledge gaps were largely positive and were generally underpinned by beliefs that education and knowledge can empower individuals to make more informed decisions about DRR behaviours or directly enable change*.*

*Understanding dementia risk reduction*

Four main existing theories of how to reduce dementia risk were evident: staying cognitively active [1, 4, 6, 11-15, 17-23], staying socially active [12, 15, 21, 22, 24-26], staying physically active [3, 4, 6, 12, 14, 15, 17, 18, 23, 27] and eating the right things [4, 6, 12, 14, 15, 17, 18, 20-22, 25-27]. Less commonly, theories of dementia risk reduction also encompassed medications, supplements and management of medical conditions [1, 14, 17-19, 21, 26-28], maintaining mental health and wellbeing [12, 15, 17, 21, 22], and avoiding smoking, drugs and alcohol [1, 6]. Where considered, some but not all individuals believed multiple activities could contribute [1, 3, 12, 13, 16-18, 20-22, 26, 27]. Individuals operationalised broad theories into specific activities. These frequently referenced interests or life circumstances [4, 6, 12, 13, 15, 17, 29], including culture [4, 14, 15, 17, 22, 24], community type (rural or urban) [24, 25, 27] and socioeconomic circumstances [7, 12, 14, 15].

*Need for information*

Gaps in knowledge and understanding were prominent [1, 3, 6, 7, 14, 17, 21, 30], with little change evident over time, (e.g., [6]). Some were subjectively acknowledged [3, 7, 14, 17, 21, 28], but authors also described a range of unacknowledged misunderstandings about dementia [1], the concept of dementia risk reduction [30] [1, 7, 21] and specific recommendations [3, 17]. Commonly, individuals believed activities promoted general health but lacked knowledge of specific cognitive benefits [3, 7, 28, 30]. Knowledge gaps were commonly identified as significant barriers to behavior change [1, 7]. Positive attitudes to addressing knowledge gaps predominated, particularly where gaps were acknowledged [1, 3, 5, 7, 10, 11, 13, 14, 21, 26-28, 30-35]. Both general dementia risk reduction information [7, 11, 13, 14, 21, 26, 31, 34] and specific behavioral guidance [3, 5, 7, 10, 13, 14, 21, 26, 30-33, 35] were sought. Attitudes to individualised dementia risk reduction information were more cautious overall, linked to the implication of receiving personalised risk status information [5, 10, 33, 34]. A few individuals related this to fear of developing dementia and a tendency to deny being at risk [8, 34].

*Education empowers choice and behaviour change*

Respondents in most studies, including intervention evaluations and observational studies, perceived information or education to be helpful for changing dementia risk reduction related behaviors [1, 3, 7, 10, 11, 13, 14, 18, 29, 30, 33-40]. Some believed education alone could directly prompt behavior change, often through increasing specific understanding of recommended behavior [1, 10, 18, 29, 30, 36-38, 40]. Others saw education as necessary but insufficient alone for dementia risk reduction behavior change and emphasised the need for complementary interventions or supports [1-3, 7, 10, 11, 13, 14, 33-35, 37, 39]. These individuals often believed education supported better informed individual decisions. The perceived benefits of education appeared linked to increasing knowledge as some individuals considered it unhelpful where they believed they had not learned anything new [32]. The effectiveness of education for prompting DRR behaviour change was notably questioned by carers [7, 22, 26, 27].

***The dementia risk reduction value proposition***

Individuals ascribed an overall value to DRR in reviewed studies. They focussed on the perceived value of lower dementia risk as an outcome, the perceived plausibility of achieving this outcome through DRR related behaviour patterns, and other perceived benefits from activities associated with DRR. Many weighed up anticipated costs of adopting DRR behaviour patterns against identified benefits. These influenced attitudes to DRR education and beliefs about the impact of education on behaviour. As with education, valuing DRR highly was sufficient for some individuals to change behaviour, but the intentions of others were also influenced by additional beliefs and opinions about individual activities (e.g., preferences, enablers and barriers).

*The value of reducing dementia risk*

Where individuals believed that reducing dementia risk is plausible, a large majority perceived this to be an outcome of substantial value [1, 3, 5, 6, 8, 12, 24, 26, 27, 29, 31, 34, 38]. Some particularly feared dementia [1, 26, 31]. Others emphasised anticipated functional and quality of life (QOL) consequences of maintaining cognitive health in old age [6, 8, 12, 24, 26, 29]. A smaller number of individuals perceived reduced burden for families or society as the main source of value [8, 23, 26]. A few individuals ascribed little value to a lower risk of dementia, usually related to a broader devaluation of later life [9, 32, 34], but, for a small number, linked to a lack of concern about dementia and its consequences [3, 32]. Most equated lower dementia risk with definitive dementia prevention and appraised that outcome, and there was more variability in the perceived value of dementia being merely less likely or delayed [5, 6, 8, 12, 24, 26, 27, 34, 38].

*The plausibility and effectiveness of dementia risk reduction*

Four stances on dementia risk reduction plausibility and effectiveness predominated. Most commonly, individuals believed that dementia is preventable and appraised dementia risk reduction interventions, particularly the lifestyle behaviors highlighted above, as important or effective for this outcome [3, 4, 6, 9, 12, 16, 18, 22-24, 27, 29, 35, 38, 41]. A significant number, however, subscribed to myths that dementia is a normal part of ageing or believed interventions would not have any effect on dementia risk [1, 3, 9, 10, 16, 21, 24, 26, 27]. Myths regarding dementia were more pervasive in older papers but were still evident in more recent studies. Some people perceived dementia risk reduction as useful in general but futile in the face of genetic risk factors or family history [20, 26, 27]. Others, however, believed dementia risk reduction may be of some benefit despite genetic vulnerability [10, 11, 20, 26, 28, 35, 40].

*The value contribution of other benefits*

Perceived or anticipated benefits other than reducing dementia risk substantially contributed to the overall valued ascribed to dementia risk reduction for many people [2, 3, 6, 8, 11, 12, 14, 22, 23, 29, 31, 32, 35, 37, 39, 41]. People also valued QOL, mental and physical wellbeing [3, 6, 12, 14, 22, 23, 29, 31, 32, 37, 39], enjoyment or interest [2, 3, 8, 11, 29, 31, 35, 37, 41] and increased sociability [14, 23, 31, 37] as outcomes of dementia risk reduction interventions. These were sometimes considered more important than reducing dementia risk. Some individuals also valued broader meaning and benefits for the community (not limited to perceived benefits of lower dementia burden) [2, 3].

*Weighing costs of dementia risk reduction*

Some individuals weighed up anticipated negative outcomes against perceived benefits [1, 4, 5, 7, 8, 12, 14, 20, 23, 25, 27, 28, 35, 39, 41], particularly where they anticipated these were likely. This was most evident in individual value propositions for diet or medication interventions. Negative outcomes of importance tended to relate to broad categories of losing pleasure or enjoyment (particularly for diet) [1, 4, 27]; experiencing unpleasant effects of side-effects (particularly from medication) [5, 8, 23, 28, 35]; and having less time or, attention or other resources to allocate to for competing demands or roles (e.g., caring or occupational roles) demands [1, 7, 12, 14, 20, 25, 39, 41]. Resourcing more basic activities to support immediate survival were important competing demands weighed up by some people, and some authors associated this with individuals experiencing socioeconomic disadvantage [12]. Finally, when discussed, neglecting caring or work responsibilities were usually considered an unacceptable cost of any dementia risk reduction activity [1, 20, 25, 39].

***Buying-in to dementia risk reduction***

Buy-in depended on perceiving advice as trustworthy and reliable; perceiving certainty in outcomes; and, identifying with the perceived target populations of advice and interventions. People were less persuaded by advice from unreliable sources, or that was inconsistent or indefinite. Some people also avoided information that caused uncomfortable emotions, particularly fear.

*Trusting sources*

Individuals tended to judge the trustworthiness and reliability of dementia risk reduction information and advice according to its source, and some individuals directly linked the source of information to forming intentions to act on it [7, 14, 16, 32]. Both professional expertise and lived experience contributed to perceived reliability. Healthcare professionals, churches, universities, government bodies and dementia-related non-governmental organisations (NGOs) were all highlighted as trusted expert sources [3, 4, 7, 11, 14, 16, 21, 29, 32, 34]. Some individuals asserted that trusting these individuals still required a longitudinal or personal relationship [11, 32, 34], but only one study reported general distrust of experts [1]. Individuals in four studies perceived peers and family networks as having lived experience that made them trusted sources of information, which a few explicitly linked to greater buy-in and taking action [3, 4, 14, 21].

Mass media (television, radio, newspapers) were generally seen as unreliable sources of information [7, 14, 21], but were considered useful for increasing awareness [1, 3, 14, 21, 27]. Internet sources were only considered by a few recent studies and perspectives regarding the reliability of internet sourced information were mixed [7, 40]. A few individuals advocated developing awareness through mass media, and personalised information through healthcare professionals [14, 21].

*Seeking certainty*

A desire for definitive research findings and for a guaranteed outcome from dementia risk reduction interventions was common and appeared vital to buying-in to advice for some [1, 3-5, 10, 14, 16, 17, 20, 28, 33, 34]. Individuals sought certainty at multiple levels, including guaranteed prevention [1, 3, 4, 10, 16, 17, 28, 33, 34] and immediate benefits [1, 5, 20]. Gaps in evidence regarding dementia aetiology, the probabilistic nature of dementia risk reduction, conflicting dementia risk reduction research results and changing advice, and the significant time lag between dementia risk reduction behaviors and outcomes generated skepticism regarding the legitimacy of advice for some. For others, uncertainty did not impact overall perceptions of dementia risk reduction as a concept, but diminished perceptions of immediate personal relevance and, consequently, diminished intentions to change behavior [3, 4, 10, 20, 28, 34].

*Avoidance*

Some individuals found it challenging to acknowledge being at risk of a condition they feared [3, 7, 8, 34], and ageing and mortality being commonly associated with risk of dementia [23, 34]. This was linked to a reluctance to consider dementia risk reduction personally relevant and avoiding information about dementia risk reduction [3, 8, 23, 34]. This response was more evident where individuals subscribed to myths regarding dementia and ageing [8, 34]. Avoiding existentially confronting associations also manifest in some individuals as strong preferences for positive messaging in dementia risk reduction interventions, including preferring dementia risk reduction information that highlights protective measures without incorporating any information about risk status [3, 34]. Only a few individuals believed that being confronted with feared associations of dementia was motivating for dementia risk reduction buy-in and advocated using fear messaging [3].

***Turning beliefs into action***

Regardless of beliefs regarding DRR in general, beliefs and opinions about individual activities influenced decisions to adopt and maintain them. These included intervention preferences, individual will and beliefs about individual capacity to adopt and maintain specific new behaviour patterns. These usually influenced DRR behaviour after the concept was already understood, valued, and believed to be legitimate and personally applicable. In turn, feedback from intervention experience moderated many of these beliefs, both amplifying and diminishing their initial impact.

*The impact of intervention characteristics*

Preferred intervention characteristics were described by individuals in 19 studies [2, 3, 7, 11, 14, 15, 21, 22, 25, 28-32, 34, 35, 37, 39, 41]. Participants often described the concordance between an intervention and their preferences as directly influencing decisions about taking up or sustaining behaviors, particularly those related to lifestyle [2, 7, 11, 14, 25, 28, 29, 31, 32, 35, 37, 39, 41]. Individual preferences were diverse but most related to perceived intervention accessibility and convenience, usability and adaptability [2, 3, 7, 11, 14, 15, 29-32, 34, 35, 37, 39, 41]. The latter were particularly sought to accommodate different skill levels or common physiological changes of ageing. Aesthetic appeal was important to some [11, 35, 41]. Reflecting the diverse preferences seen, several individuals emphasised an overarching need for choice in intervention characteristics [3, 7, 11, 14, 34]. Concordance with broader interests and values was highlighted by a small number of individuals across several studies [11, 21, 22, 25, 28, 29]: upholding autonomy, avoiding medications, and maintaining culturally based engagement with nature were perceived as very important. For some, intervention preferences crystalised and exerted greater influence on behavior following initial experience of an activity, while others moderated or changed their views with time [11, 32, 35, 37, 41].

*The importance of personal will*

While only considered in a smaller number of studies, some individuals emphasised a key role of personal will in decisions to take-up and maintain dementia risk reduction behaviors, after establishing conducive conceptual beliefs [6, 7, 12, 14, 20, 32, 35, 39]. Individuals described making an active choice to change behavior and the strength of that desire as important to enacting intentions. They believed this helped them to take-up and sustain dementia risk reduction behaviors where they already endorsed activities as valuable and personally relevant [6, 12, 20, 32, 39]. Similarly, individuals in nine studies perceived reluctance to take-up behavior as related to the absence of personal will [6, 7, 14, 35]. They were pessimistic about the likely impact of interventions to influence behavior where there was low will to change. Further, some contended that directive advice or coercive measures could be counterproductive and escalate resistance in reluctant individuals, and advocated respecting individual choices [7, 11, 14, 32, 35].

*Reciprocity between self-efficacy and behavior*

Low confidence in skills or personal capacity to successfully undertake behavior and influence cognitive outcomes was raised as a barrier to engaging in dementia risk reduction by a small number of individuals [2, 6, 7, 22, 29, 39, 41]. This acted despite an overall endorsement of dementia risk reduction and specific activities as valuable. Physical health comorbidities and disabilities were the most commonly cited reasons for these beliefs. Other individuals described low self-efficacy increasing the perceived difficulty of engaging in behavior without acting as an absolute barrier [35, 39, 41]. Experience, particularly where successful, had a notable positive impact on self-efficacy for future take-up and for sustaining existing dementia risk reduction behaviors [30, 35, 37, 39, 41]. Success was usually defined as being able to engage with activities. Some also attributed increased self-efficacy to unexpected benefits from participation, particularly social interaction [37].

***The impact of social factors***

Individuals described social factors as strongly impacting intentions and the behaviors taken-up and sustained. Beliefs about social factors also tended to build on base conceptual beliefs but they were less reliant on concordance with these. Individuals described an influence for social factors via three main pathways: social expectations and obligations enabling dementia risk reduction engagement; social interactions with clinicians or others delivering interventions influencing intentions for individual activities; and peer support and peer examples as helping enactment of intentions to take-up dementia risk reduction behaviors.

*Social expectations*

Social expectations and obligations directly facilitated engagement with dementia risk reduction for some people. Anticipated or perceived opportunities for social interaction strongly drove reported intentions to take-up interventions for some people [3, 15, 22, 23, 29-31, 37, 41]. This related to broadly valuing social interaction as a benefit of interventions. Expecting increased socialisation often motivated people to change behaviour despite seemingly limited understanding of dementia risk reduction or without considering the personal value of lower dementia risk. For others, a sense of social obligation related to accountability driven by laws/regulations, social norms or a reality-based sense of being accountable to others, such as ‘buddies’ [1, 3, 24, 31, 34, 39]. Finally, a few described an internalised or internally generated sense of accountability. This included perceived obligations to physicians or family and a general sense of dependability [29, 31, 39].

*Delivery of interventions*

Social aspects of the interaction between healthcare workers or other professionals involved in dementia risk reduction intervention delivery were highlighted as either enabling or inhibiting dementia risk reduction take-up, particularly in intervention evaluations [7, 11, 14, 32, 34, 39, 41]. Communication style, continuity and reciprocity in sharing personal information were all specifically referenced. There was an almost universal preference for interactions that were positive and collaborative, with individual circumstances and choices respected and accommodated [7, 11, 32, 34, 39, 41]. Interactions perceived to be generic, patronising or directive were viewed negatively, and some individuals attributed drop-out from intervention studies to this interaction style [14, 32, 41].

*The importance of peer support and examples*

Perspectives regarding social supports tended to focus on peers. Individuals highlighted the benefits of informal peer support and of positive peer examples. Peers were commonly seen as a powerful source of encouragement and as helpful for overcoming other barriers, again, generally in the context of already valuing an activity [2, 3, 14, 23, 31, 35]. Some people also reflected that observing and comparing oneself to peers with similar functional challenges or with significant disabilities was a powerful motivator [11, 14]. Further, some individuals described a sense of responsibility to themselves set a positive example and provide peer support. They were confident that this would effectively facilitate dementia risk reduction take-up in others [14, 23]. Workplaces, family and more formal support services were also cited as helpful sources of support. A few individuals perceived their main role to be where informal or peer supports were not available [1, 3, 34].

***Personal experience***

Personal experience, observing others (particularly family members living with dementia), inherited wisdom from family or community, and culturally bound beliefs all substantially influenced each theme. These influences contributed to the wide spectrum of final individual belief systems seen. Personal experience was commonly emphasised over expert advice in forming individual perspectives, but there was some suggestion that experience-based attribution biases were amenable to correction through education.

The strongest impact of personal experience was evident for individual understanding of dementia risk reduction and individual value propositions. People often understood dementia risk reduction to involve activities they had observed in cognitively healthy older people [7, 12, 13, 15, 24, 26, 27], or that they associated with historical periods in which they believed dementia was less common [22, 25]. Others held theories of dementia risk reduction that were based on inherited wisdom, cultural beliefs [17, 27] or activities they had previously experienced as providing immediate cognitive benefits [17].

Wishing to avoid the experience of people they had observed living with dementia was a common reason for highly valuing reducing dementia risk [8, 10, 25, 26, 29, 31, 35]. The perceived plausibility of dementia risk reduction and the perceived effectiveness of specific dementia risk reduction activities were also frequently linked to personal associations with individuals who did or did not develop dementia and scepticism often emerged where personal experience conflicted with expert information or recommendations [1, 4, 9, 15, 16, 20, 21, 26].

Finally, personal experience, observing others or received wisdom about what one is capable of (e.g., at a given chronological age or despite a disability) influenced intentions and perceptions about take-up of specific behaviors through anticipated benefits [31, 32, 37, 41], self-efficacy [35, 39, 41], and preferences for intervention characteristics [2, 29, 35, 39, 41].

**Table 4. Outline of corroborated themes and additional insights from articles added through search updates**

| **Paper** | **Existing themes corroborated by paper** | **Additional insights** |
| --- | --- | --- |
| Akenine et al., 2020 [42] | Corroborated the need for effective education, including: existing theories of dementia; the need for information to address diverse knowledge gaps (procedural knowledge); and empowerment through education. Corroborated need for information to be reliable and sources trusted ( existing healthcare professionals) to empower action; the importance of understanding personal circumstance, adapting advice, and maintaining autonomy; and dementia fear and stigma as barriers to accessing information and behaviour change, particularly where knowledge limited. Corroborated interaction between themes as influencing engagement in dementia risk reduction behaviour and influence of personal context. | Extended knowledge gaps to include when in lifespan to act. Extended emphasis on trusted sources to specify preference for known healthcare professionals to optimise trust and collaboration. Extended social context influences to include healthcare system contextual factors. |
| Bacsu et al., 2020 [43] | Corroborated the need for effective education, particularly existing theories of dementia risk reduction and gaps in MRF knowledge. Corroborated the importance of perceiving short-term value in activities (particularly anticipating enjoyment). Corroborated the benefits of access to supports and resources for self-regulation and social opportunities that are relevant to personal circumstances. Corroborated the influence on beliefs of heuristic knowledge based on personal experiences. | Extended views regarding social opportunities to include empowerment through provision of easily accessible physical spaces for social opportunities. |
| Bosco et al., 2020 [44] | Corroborated need for effective education, particularly that barriers to behaviour change include misunderstandings about adequacy of current behaviour for dementia risk reduction. Corroborated that perceived value of activities for lowering dementia risk and perceived personal relevance contribute to behaviour change intentions after weighing against perceived costs to other roles and priorities, particularly caring responsibilities. Corroborated need for reliable information from trusted sources (health professionals), and scepticism of inconclusive evidence. | Extended perceived costs of dementia risk reduction weighed up by some to include costs to activities for other health priorities |
| Cooper et al., 2021 [45] | Corroborated need for sensitive supports that are positive and do not prompt anxiety. Corroborated that social opportunities are important and can facilitate behaviour change, particularly through contributing to the perceived value of interventions, and that these should be empowering, support rather than impede autonomy, provide positive peer examples and facilitate peer-support. | Extended benefits of empowering social opportunities to include that peer comparisons are reassuring; and that technology can facilitate compensatory social contact in the Covid-19 pandemic context |
| Halloway et al., 2020 [46] | Focused specifically on physical activity, but corroborated several general themes. Corroborated that physical health problems can impair self-efficacy but that relevant self-regulatory supports and even small behaviour changes can increase self-efficacy and diminish physical health as a barrier. Corroborated choice for supports as key to improving effectiveness. Corroborated the need for effective education for conceptual and procedural knowledge gaps. Corroborated importance of function and quality of life benefits for perceived value. | Extended theme that trusted expert sources of advice help individuals to buy-in to changing behaviour to specify one mechanism as being through increasing self-efficacy despite medical conditions. |
| Largent et al., 2020 [47] | Corroborated need for education to address misunderstandings in theories of dementia prevention. Corroborated that perceived value includes perceived dementia risk reduction effectiveness and other short- and long-term benefits (enjoyment). Corroborated that buy-in to behaviour change influenced by beliefs about personal relevance of dementia risk reduction, with anxiety about possible future dementia as a barrier. |  |
| McGrattan et al., 2021  [48] | Corroborated empowerment through comprehensive education. Corroborated that perceived value includes perceived effectiveness for dementia prevention and other short- and long-term benefits (general health and wellbeing). Corroborated the importance of self-regulation supports to enact intentions, particularly supports for memory, attention and decision-making for people with MCI, and diverse preferences overall. |  |
| Swindells et al., 2020 [49] | Corroborated need for effective education, particularly for knowledge gaps in existing theories of dementia risk reduction (interactions between modifiable- and non-modifiable risk factors and procedural knowledge); and that education can empower individuals. Corroborated that perceived plausibility in the setting of genetic risk influences perceived value of activities. Corroborated the need for trusted sources and strong supporting evidence on individual buy-in to advice. Corroborated the importance of respectful, personalised and collaborative intervention planning to facilitate intentions and action. Corroborated personal experiences strongly influencing beliefs and behaviour. | Extended theme of the need for effective education by highlighting key knowledge gap regarding when in the lifespan to change behaviour for dementia prevention. |
| Wesselman et al., 2020 [50] | Corroborated the benefits of self-regulatory supports, choice and control for optimally tailored interventions that are more likely to be actioned. Corroborated the need for effective education, including existing knowledge gaps (conceptual knowledge) and, empowerment through education. Corroborated the importance of a trustworthy source for information. Corroborated that perceived value influenced by perceived short-term benefits (health and quality of life, opportunities for social interaction and enjoyment). |  |

**References**

1. Kim S, Sargent-Cox KA, Anstey KJ. A qualitative study of older and middle-aged adults' perception and attitudes towards dementia and dementia risk reduction. J Adv Nurs. 2015;71(7):1694-703. doi: 10.1111/jan.12641. PubMed PMID: WOS:000356624700021.

2. Croff RL, Witter P, Walker ML, Francois E, Quinn C, Riley TC, et al. Things Are Changing so Fast: Integrative Technology for Preserving Cognitive Health and Community History. Gerontologist. 2019;59(1):147-57. doi: 10.1093/geront/gny069. PubMed PMID: WOS:000462568400019.

3. Price AE, Corwin SJ, Friedman DB, Laditka SB, Colabianchi N, Montgomery KM. Older adults' perceptions of physical activity and cognitive health: implications for health communication. Health Educ Behav. 2011;38(1):15-24. Epub 2010/12/31. doi: 10.1177/1090198110369764. PubMed PMID: 21191085.

4. Wu B, Goins RT, Laditka JN, Ignatenko V, Goedereis E. Gender differences in views about cognitive health and healthy lifestyle behaviors among rural older adults. Gerontologist. 2009;49 Suppl 1:S72-8. Epub 2009/07/07. doi: 10.1093/geront/gnp077. PubMed PMID: 19525219.

5. Lawrence V, Pickett J, Ballard C, Murray J. Patient and carer views on participating in clinical trials for prodromal Alzheimer's disease and mild cognitive impairment. Int J Geriatr Psych. 2014;29(1):22-31. doi: 10.1002/gps.3958. PubMed PMID: WOS:000327823400002.

6. Mattos MK, Burke LE, Baernholdt M, Hu L, Nilsen ML, Lingler JH. Perceived Social Determinants of Health Among Older, Rural-Dwelling Adults with Early-Stage Cognitive Impairment. Dementia (London). 2019;18(3):920-35. Epub 2017/04/01. doi: 10.1177/1471301217694250. PubMed PMID: 28361578; PubMed Central PMCID: PMCPMC5519446.

7. Neville CE, McCourt HJ, McKinley MC, Lowis C, Barrett SL, McGuinness B, et al. Encouraging lifestyle behaviour change in mild cognitive impairment patients: development of appropriate educational material. Aging Ment Health. 2013;17(3):276-86. Epub 2013/02/14. doi: 10.1080/13607863.2013.768210. PubMed PMID: 23402379.

8. Watson J, Saunders S, Muniz Terrera G, Ritchie C, Evans A, Luz S, et al. What matters to people with memory problems, healthy volunteers and health and social care professionals in the context of developing treatment to prevent Alzheimer's dementia? A qualitative study. Health Expect. 2019;22(3):504-17. Epub 2019/02/28. doi: 10.1111/hex.12876. PubMed PMID: 30809895; PubMed Central PMCID: PMCPMC6543163.

9. Corner L, Bond J. Being at risk of dementia: Fears and anxieties of older adults. J Aging Stud. 2004;18(2):143-55. doi: 10.1016/j.jaging.2004.01.007. PubMed PMID: WOS:000221321500002.

10. Milne R, Diaz A, Badger S, Bunnik E, Fauria K, Wells K. At, with and beyond risk: expectations of living with the possibility of future dementia. Sociol Health Ill. 2018;40(6):969-87. doi: 10.1111/1467-9566.12731. PubMed PMID: WOS:000438368900003.

11. Haesner M, O'Sullivan JL, Govercin M, Steinhagen-Thiessen E. Requirements of older adults for a daily use of an internet-based cognitive training platform. Inform Health Soc Care. 2015;40(2):139-53. Epub 2014/04/15. doi: 10.3109/17538157.2013.879149. PubMed PMID: 24725153.

12. Bardach SH, Benton B, Walker C, Alfred DL, Ighodaro E, Caban-Holt A, et al. Perspectives of African American Older Adults on Brain Health: "Brains Get Tired Too". Alzheimer Dis Assoc Disord. 2019;33(4):354-8. Epub 2019/07/25. doi: 10.1097/WAD.0000000000000335. PubMed PMID: 31335456; PubMed Central PMCID: PMCPMC7181952.

13. Hulko W, Camille E, Antifeau E, Arnouse M, Bachynski N, Taylor D. Views of First Nation elders on memory loss and memory care in later life. J Cross Cult Gerontol. 2010;25(4):317-42. Epub 2010/07/02. doi: 10.1007/s10823-010-9123-9. PubMed PMID: 20593232.

14. Friedman DB, Laditka JN, Hunter R, Ivey SL, Wu B, Laditka SB, et al. Getting the Message Out About Cognitive Health: A Cross-Cultural Comparison of Older Adults' Media Awareness and Communication Needs on How to Maintain a Healthy Brain. Gerontologist. 2009;49. doi: 10.1093/geront/gnp080. PubMed PMID: WOS:000267940200008.

15. Friedman DB, Laditka SB, Laditka JN, Wu B, Liu R, Price AE, et al. Ethnically Diverse Older Adults' Beliefs About Staying Mentally Sharp. Int J Aging Hum Dev. 2011;73(1):27-52. doi: 10.2190/AG.73.1.b. PubMed PMID: WOS:000294649300002.

16. Laditka JN, Laditka SB, Liu R, Price AE, Wu B, Friedman DB, et al. Older adults' concerns about cognitive health: commonalities and differences among six United States ethnic groups. Ageing Soc. 2011;31:1202-28. doi: 10.1017/S0144686x10001273. PubMed PMID: WOS:000295598300008.

17. Wilcox S, Sharkey JR, Mathews AE, Laditka JN, Laditka SB, Logsdon RG, et al. Perceptions and beliefs about the role of physical activity and nutrition on brain health in older adults. Gerontologist. 2009;49 Suppl 1:S61-71. Epub 2009/07/07. doi: 10.1093/geront/gnp078. PubMed PMID: 19525218.

18. Arias JJ, Cummings J, Grant AR, Ford PJ. Stakeholders' Perspectives on Preclinical Testing for Alzheimer's Disease. J Clin Ethics. 2015;26(4):297-305. Epub 2016/01/12. PubMed PMID: 26752383.

19. Banningh LJW, Vernooij-Dassen M, Rikkert MO, Teunisse JP. Mild cognitive impairment: coping with an uncertain label. Int J Geriatr Psych. 2008;23(2):148-54. doi: 10.1002/gps.1855. PubMed PMID: WOS:000253472100005.

20. Lock M, Freeman J, Chilibeck G, Beveridge B, Padolsky M. Susceptibility genes and the question of embodied identity. Med Anthropol Q. 2007;21(3):256-76. doi: 10.1525/Maq.2007.21.3.256. PubMed PMID: WOS:000249411600003.

21. Marcum ZA, Hohl SD, Gray SL, Barthold D, Crane PK, Larson EB. Brain Health and Dementia Prevention: A Mixed-method Analysis. Am J Health Behav. 2019;43(2):300-10. doi: 10.5993/Ajhb.43.2.7. PubMed PMID: WOS:000459782200007.

22. Pace J. "Place-ing" Dementia Prevention and Care in NunatuKavut, Labrador. Can J Aging. 2020;39(2):247-62. Epub 2019/11/02. doi: 10.1017/S0714980819000576. PubMed PMID: 31666149.

23. Thogersen-Ntoumani C, Papathomas A, Foster J, Quested E, Ntoumanis N. "Shall We Dance?" Older Adults' Perspectives on the Feasibility of a Dance Intervention for Cognitive Function. J Aging Phys Act. 2018;26(4):553-60. Epub 2017/12/29. doi: 10.1123/japa.2017-0203. PubMed PMID: 29283746.

24. Traphagan J. Localizing senility: illness and agency among older Japanese. Journal of Cross-Cultural Gerontology. 1998;13:81-98.

25. Eisenhauer CM, Pullen CH, Hunter JL, Nelson T. The influence of cognitive decline on rural identity: perspectives of older women. J Holist Nurs. 2015;33(2):134-45. Epub 2014/08/08. doi: 10.1177/0898010114544218. PubMed PMID: 25098734.

26. Kim JS, Kim EH, An M. Experience of Dementia-related Anxiety in Middle-aged Female Caregivers for Family Members with Dementia: A Phenomenological Study. Asian Nurs Res. 2016;10(2):128-35. doi: 10.1016/j.anr.2016.02.001. PubMed PMID: WOS:000378541400007.

27. Wiese LK. An Appalachian Perspective of Alzheimer’s disease: A Rural Health Nurse Opportunity. Online Journal of Rural Nursing and Health Care. 2018;18(1):180-208. doi: 10.14574/ojrnhc.v17i1.469.

28. Marcum ZA, Hohl SD, Gray SL, Barthold D, Crane PK, Larson EB. Patient Perceptions of Antihypertensive Use as a Dementia Prevention Strategy: A Mixed-Method Analysis of a Web-Based Survey. Journal of Alzheimers Disease. 2019;68(2):523-9. doi: 10.3233/Jad-181080. PubMed PMID: WOS:000462875000010.

29. Coley N, Rosenberg A, van Middelaar T, Soulier A, Barbera M, Guillemont J, et al. Older Adults' Reasons for Participating in an eHealth Prevention Trial: A Cross-Country, Mixed-Methods Comparison. J Am Med Dir Assoc. 2019;20(7):843-9 e5. Epub 2018/12/14. doi: 10.1016/j.jamda.2018.10.019. PubMed PMID: 30541689.

30. Fogarty J, Farrell B, Gutmanis I. Promoting healthy living for seniors: evaluation of a community-based program. Therapeutic Recreation Journal. 2014;48(3):262-74.

31. Etnier JL, Karper WB, Park SY, Shih CH, Piepmeier AT, Wideman L. Motivating Mature Adults to be Physically Active. J Aging Phys Act. 2017;25(2):325-31. Epub 2016/09/14. doi: 10.1123/japa.2015-0294. PubMed PMID: 27620855; PubMed Central PMCID: PMCPMC5368032.

32. Ligthart SA, van den Eerenbeemt KDM, Pols J, van Bussel EF, Richard E, van Charante EPM. Perspectives of older people engaging in nurse-led cardiovascular prevention programmes: a qualitative study in primary care in the Netherlands. Brit J Gen Pract. 2015;65(630):E41-E8. doi: 10.3399/bjgp15X683149. PubMed PMID: WOS:000348520400006.

33. Milne R, Bunnik E, Diaz A, Richard E, Badger S, Gove D, et al. Perspectives on Communicating Biomarker-Based Assessments of Alzheimer's Disease to Cognitively Healthy Individuals. Journal of Alzheimers Disease. 2018;62(2):487-98. doi: 10.3233/Jad-170813. PubMed PMID: WOS:000426042100001.

34. Robinson L, Dickinson C, Magklara E, Newton L, Prato L, Bamford C. Proactive approaches to identifying dementia and dementia risk; a qualitative study of public attitudes and preferences. BMJ Open. 2018;8(2):e018677. Epub 2018/02/13. doi: 10.1136/bmjopen-2017-018677. PubMed PMID: 29431130; PubMed Central PMCID: PMCPMC5829774.

35. Walker JE, Thompson KE, Oliver AI. Maintaining Cognitive Health in Older Adults: Australians’ Experience of Targeted Computer-Based Training, using the Brain Fitness Program. Physical & Occupational Therapy In Geriatrics. 2014;32(4):397-413. doi: 10.3109/02703181.2014.965377.

36. Grill JD, Cox CG, Harkins K, Karlawish J. Reactions to learning a "not elevated" amyloid PET result in a preclinical Alzheimer's disease trial. Alzheimers Res Ther. 2018;10(1):125. Epub 2018/12/24. doi: 10.1186/s13195-018-0452-1. PubMed PMID: 30579361; PubMed Central PMCID: PMCPMC6303934.

37. Hassan S, Aguirre E, Betz A, Robertson S, Sankhla D, Cooper C. Evaluating the effect of Brainfood groups for people with mild cognitive impairment and mild dementia: preliminary mixed-methodology study. BJPsych Open. 2018;4(4):208-14. Epub 2018/07/11. doi: 10.1192/bjo.2018.29. PubMed PMID: 29988927; PubMed Central PMCID: PMCPMC6034463.

38. Hurley AC, Harvey FR, Roberts JS, Wilson-Chase C, Lloyd S, Prest J, et al. Genetic susceptibility for Alzheimer's disease: why did adult offspring seek testing? Am J Alzheimers Dis Other Demen. 2005;20(6):374-81. Epub 2006/01/07. doi: 10.1177/153331750502000608. PubMed PMID: 16396443.

39. Nelis SM, Thom JM, Jones IR, Hindle JV, Clare L. Goal-setting to Promote a Healthier Lifestyle in Later Life: Qualitative Evaluation of the AgeWell Trial. Clin Gerontologist. 2018;41(4):335-45. doi: 10.1080/07317115.2017.1416509. PubMed PMID: WOS:000432151100008.

40. Zallen DT. "Well, good luck with that": reactions to learning of increased genetic risk for Alzheimer disease. Genet Med. 2018;20(11):1462-7. Epub 2018/03/09. doi: 10.1038/gim.2018.13. PubMed PMID: 29517767.

41. O’Brien D, Knapp RB, Thompson O, Craig D, Barrett S. An exploration of seniors’ motivation to use mobile brain-exercise software. Gerontechnology. 2013;11(3). doi: 10.4017/gt.2013.11.3.002.00.

42. Akenine U, Barbera M, Beishuizen CR, Fallah Pour M, Guillemont J, Rosenberg A, et al. Attitudes of at-risk older adults about prevention of cardiovascular disease and dementia using eHealth: a qualitative study in a European context. BMJ Open. 2020;10(8):e037050. Epub 2020/08/09. doi: 10.1136/bmjopen-2020-037050. PubMed PMID: 32764085; PubMed Central PMCID: PMCPMC7412614.

43. Bacsu J-D, Viger M, Johnson S, Novik N, Jeffery B. Rural Older Adults’ Perspectives of Activities to Support Cognitive Health. Activities, Adaptation & Aging. 2019;44(3):177-91. doi: 10.1080/01924788.2019.1626203.

44. Bosco A, Jones KA, Di Lorito C, Stephan BCM, Orrell M, Oliveira D. Changing lifestyle for dementia risk reduction: Inductive content analysis of a national UK survey. PLoS One. 2020;15(5):e0233039. Epub 2020/05/16. doi: 10.1371/journal.pone.0233039. PubMed PMID: 32413085; PubMed Central PMCID: PMCPMC7228104.

45. Cooper C, Mansour H, Carter C, Rapaport P, Morgan-Trimmer S, Marchant NL, et al. Social connectedness and dementia prevention: Pilot of the APPLE-Tree video-call intervention during the Covid-19 pandemic. Dementia (London). 2021:14713012211014382. Epub 2021/04/30. doi: 10.1177/14713012211014382. PubMed PMID: 33913362.

46. Halloway S, Wilbur J, Schoeny ME, Braun LT, Aggarwal NT, Miller AM, et al. Feasibility of a Lifestyle Physical Activity Intervention to Prevent Memory Loss in Older Women With Cardiovascular Disease: A Mixed-Methods Approach. Can J Nurs Res. 2020;52(4):278-89. Epub 2019/07/02. doi: 10.1177/0844562119856233. PubMed PMID: 31256633.

47. Largent EA, Harkins K, van Dyck CH, Hachey S, Sankar P, Karlawish J. Cognitively unimpaired adults' reactions to disclosure of amyloid PET scan results. PLoS One. 2020;15(2):e0229137. Epub 2020/02/14. doi: 10.1371/journal.pone.0229137. PubMed PMID: 32053667; PubMed Central PMCID: PMCPMC7018056.

48. McGrattan AM, McEvoy CT, Vijayakumar A, Moore SE, Neville CE, McGuinness B, et al. A mixed methods pilot randomised controlled trial to develop and evaluate the feasibility of a Mediterranean diet and lifestyle education intervention 'THINK-MED' among people with cognitive impairment. Pilot Feasibility Stud. 2021;7(1):3. Epub 2021/01/05. doi: 10.1186/s40814-020-00738-3. PubMed PMID: 33390187; PubMed Central PMCID: PMCPMC7780397.

49. Swindells J, Gomersall T. Public perception of dementia risk in the UK: a mental models approach. Journal of Risk Research. 2019;23(4):461-74. doi: 10.1080/13669877.2019.1591486.

50. Wesselman LMP, Schild AK, Hooghiemstra AM, Meiberth D, Drijver AJ, Leeuwenstijn-Koopman MV, et al. Targeting Lifestyle Behavior to Improve Brain Health: User-Experiences of an Online Program for Individuals with Subjective Cognitive Decline. J Prev Alzheimers Dis. 2020;7(3):184-94. Epub 2020/05/29. doi: 10.14283/jpad.2020.9. PubMed PMID: 32463072.
