# Supplementary material for: General population perspectives of dementia risk reduction and the implications for intervention: A systematic review and thematic synthesis of qualitative evidence
Source: PLoS One. 2021 Sep 17;16(9):e0257540. doi: 10.1371/journal.pone.0257540 (PMC8448319; doi:10.1371/journal.pone.0257540)
Supplement: S1 Table — (DOCX) [file pone.0257540.s004.docx]

**S1 Table. Characteristics of included studies**

| **Study** | **Cou-ntry** | **Relevant Participant type; N;**  **Age Range; Mean Age (SD)** | **Setting** | **Relevant Aims** | **Study Design (as reported by authors)** | **Theoretical Framework (as reported by authors)** | **Data Collection (as reported by authors)** | **Data Analysis (as reported by authors)** | **Relevant Topic(s)** |
| --- | --- | --- | --- | --- | --- | --- | --- | --- | --- |
| Arias et al., 2015 | US | Family caregivers of people with MCI or AD 14 Age NR | Specialist dementia clinics | To evaluate family members' perspectives on pre-clinical AD biomarker testing | Qualitative descriptive | NR | ISSIs | Adjusted approach to GT | Views on AD pre-clinical testing for DRR |
| Bardach et al., 2019 | US | African American older adults and carers of older adults 21 35-86 63.7 (NR) | Community and chronic pain support group | To understand how African Americans conceptualise brain health and their ability to influence healthy brain aging | Photovoice | NR | FGDs | TA | Views on healthy brain aging |
| Coley et al., 2019 | FI, FR, NL | Older adults participating in an eHealth prevention trial 341 66.5-74.9 Median 68.7 (NR) | Community | To explore older adults' reasons for participating in a European multinational eHealth prevention trial | Mixed-methods program evaluation | NR | ISSIs | Structured CA | Reasons for participating in lifestyle- based e-health DRR trial |
| Corner et al., 2004 | UK | Older adults 15 62-93 Median 82 (NS) | Community | To examine the views and perceptions of older people regarding the causes and consequences of cognitive impairment and dementia | Qualitative | NR | IDIs | Framework | Views and attitudes on causes and consequences of dementia |
| Croff et al., 2019 | US | Middle-aged and older African American adults participating in a health and community promotion intervention pilot 19 59-80 69.21 (5.06) | Community | To share participant perceptions of neighborhood walking and reminiscence app for cognition usefulness, usability and cultural relevancy | Mixed methods program evaluation | Technology Acceptance Model | Open-form survey and FGDs | TA, drawing from constant comparison and GT | Evaluation of combined neighborhood walking and prompted reminiscence app for DRR technology |
| Eisenhauer et al., 2015 | US | Rural older women 4 74-89 84 (NR) | Community | To examine the perspectives of community-dwelling rural, older women concerning the meaning of cognitive decline and to ascertain how cognitive decline affects their lives and the lives of those around them | Ethnography | Agrarian social ideology & Culture emergent theory (Bonder et al., 2002). | Life history IDIs | Case focused analysis | Views on meaning, causes and impacts of cognitive decline |
| Etnier et al., 2017 | US | Middle-aged and older sedentary adults with a family history of AD, participating in a trial of physical activity for cognitive health 35 49-65 NR | Community | To describe factors associated with adherence to an 8-month exercise for cognitive health program | Mixed methods program evaluation | Motivational Theory (Frederick and Ryan, 1993; Hsieh and Shannon, 2005) | Open-form computer-based survey | Qualitative CA | Evaluation of reasons for adhering to exercise sessions for DRR in participants who completed program |
| Fogarty et al., 2014 | CA | Middle-aged and older adults participating in a community healthy ageing program 48 55 - >75 NR. | Community | To evaluate a community-based program for older adults with memory concerns who are interested in learning more about what they can do to promote brain health. | Mixed methods program evaluation | Leisure ability model (Stumbo and Peterson, 2009) | SSGDs IDIs | Frequency of recorded responses to interview questions | Evaluation of community healthy brain ageing program delivery, content, and impact |
| Friedman et al., 2009 | US | Older adults identifying as African American, Chinese American, Vietnamese American, Non-Hispanic White and American Indian  177 NR. 69.8 (9.7) | Community | To describe older adults' awareness of cognitive health, and ideas about how to inform and motivate others to engage in activities that may maintain brain health | Qualitative | Diffusion of innovations model (Rogers, 1995) Elaboration likelihood model (Petty and Cacioppo, 1986) | FGDs | Qualitative TA with axial coding | Brain health information heard/seen in the mass media and recommendations for brain health promotion messages and communication strategies |
| Friedman et al., 2011 | US | Older adults identifying as African American, Chinese American, Vietnamese American, Hispanic, Non-Hispanic White and American Indian  396 NR. 71.0 (9.3) | Community | To describe perceptions about the roles of mental exercise, social activity and positive attitude in maintaining cognitive health among a diverse group of older adults living in the community; and, to examine ways that perceptions about these roles vary by ethnicity. | Qualitative | NR. | FGDs | TA using open coding;  axial coding; and, constant comparison | The relative DRR importance attributed to social involvement, mental exercise, positive attitudes, physical activity and a healthy diet |
| Grill et al., 2018 | US | Older adults who screen failed an anti-amyloid treatment in asymptomatic AD trial 33 65-83 73.6 (5.3) | Community | To explore the implications of communicating negative biomarker results to cognitively normal individuals. | Qualitative component of mixed methods design | NR. | SSIs (telephone) | TA; frequency counts of themes | Understanding of and reactions to amyloid status disclosure |
| Haesner et al., 2015 | DE | Older adults with or without MCI 12 NR NR | Geriatric hospital | To assess specific preferences and potential barriers of older adults regarding a web-based platform for cognitive training | Mixed-methods program evaluation | NR. | SSIs | Systematic CA | Preliminary evaluation of online platform for cognitive training |
| Hassan et al., 2018 | UK | Adults with MCI, participating in dietary cognitive health promotion pilot trial 13 NR. NR. | Specialist memory clinics | To evaluate a dietary cognitive health promotion intervention for acceptability and feasibility | Mixed methods program evaluation | Pender's theory of health promotion (Pender et al., 1992) | Open-form survey | TA | Process evaluation of dietary cognitive health promotion intervention |
| Hulko et al., 2010 | CA | Elders and family or community members from three First Nation communities 21 57-88 69.7 (NR.) | Community | To explore the perspectives of First Nation communities on dementia, focusing on the meanings ascribed to cognitive impairment in later life and desired relationships between the community and the health care system, from the perspectives of First Nation peoples | Constructivist GT | Anti-oppression and Indigenous perspectives on research (Brown and Strega, 2005; Kirby et al., 2006; Tuhiwai Smith, 1999; Wilson, 2008) | Sharing circles SIs with photo-elicitation | Constant comparative process | Views on cognitive health and decline |
| Hurley et al., 2005 | US | Adults with parents with AD, participating in genetic susceptibility testing trial 60 37-76 54.2 (10.2) | Community | To explore perspectives, beliefs, and motivating factors for seeking genetic susceptibility testing for AD | Glaserian GT | NR. | SSIs | Basic CA | Views on AD genetic testing impact on DRR beliefs |
| Joosten-Weyn Banningh et al., 2008 | NL | Adults with MCI 8 58-83 74.8 (8.1) | Specialist memory clinics | To investigate how patients fulfilling MCI criteria experience and cope with their cognitive decline | Qualitative | NR | SSIs | GT | Views on MCI and preferences for support-group program |
| Kim et al., 2015 | AU | Middle-aged and older adults  34 52-90 67.03 (8.85) | Community | To investigate perceptions of dementia and DRR held by people without dementia | Qualitative descriptive | Behavior change theories (collated) | FGDs | Long-table approach | Views, motivational factors and barriers re: DRR |
| Kim et al., 2016 | KR | Middle-aged females, family caregivers of persons with dementia 12 40-59 51.9 (NR) | Specialist dementia clinics | To describe the lived experience of dementia-related anxiety in middle-aged female caregivers for family members with dementia | Descriptive phenomenology | NR | SSIs | Giorgi's phenomen-ological method | Views re DRR and impact of caring for FMWD with dementia |
| Laditka et al., 2011 | US | Older adults identifying as African American, Chinese American, Vietnamese American, Hispanic, Non-Hispanic White and American Indian  396 50-90 71 (9.3) | Community | To describe concerns about cognitive function and memory among a diverse group of older adults in the USA, and to examine commonalities and differences by ethnicity | Qualitative | Theories of health behavior Theory of illness representation | FGDs | Axial coding; constant comparison | Views re: cognitive decline and DRR |
| Lawrence et al., 2014 | UK | Adults with MCI and close relatives and former carers of people with Alzheimer's disease 28 NR NR | Community | To explore how people with mild memory complaints feel about learning their biomarker status and participating in an dementia prevention medication trial | Qualitative | NR | FGDs | GT approach | Views re: learning dementia risk status for DRR and decision-making process for joining trial of imagined drug treatment for DRR |
| Ligthart et al., 2016 | NL | Older adults, participating in DRR intervention trial 15 76-82 78.5 (2.03) | Community | To explore what motivates older people to participate in long-term, nurse -led dementia prevention interventions | Qualitative | NR | SSIs | TA | Process evaluation of dementia prevention trial |
| Lock et al., 2007 | CA, US | First-degree relatives of people with AD 119 28-75 47.5 | Specialist memory clinics and community | NR | Ethnography | NR | SSIs | NR | Views re: impact of genetic testing on identity and lifestyle and beliefs regarding DRR |
| Marcum et al., 2019a | US | Adults, members of managed care consortium 1661 NR NR | Community | To assess preferences towards antihypertensive use for dementia prevention | Qualitative component of mixed-methods design | NR | Web-based open form survey | Thematic CA | Views and attitudes re: taking antihypertensive medication for dementia prevention |
| Marcum et al., 2019b | US | Adults, members of managed care consortium 1661 NR NR | Community | To assess patient knowledge, beliefs and attitudes about brain health and strategies for AD and related dementias prevention | Qualitative component of mixed-methods design | NR | Web-based open form survey | Thematic CA | Views re: how to reduce dementia risk |
| Mattos et al., 2019 | US | Older adults with MCI and care partners, participants in AD research centre cohort study 19 NR 72.3 (NR) | AD research centre | To explore perceived social determinants of health among older, rural-dwelling adults with early stage cognitive impairment | Qualitative descriptive | Social Determinants of Health and Environmental Health Promotion Model (Schulz and Northridge, 2004) | SSIs | Thematic CA | Views re: activities involved with maintaining brain health and engaging in DRR |
| Milne et al., 2018a | UK, ES, BD | Older adults, first degree relatives of persons with AD and caregivers of people with dementia, participating in dementia related cohort studies 48 (without dementia) 43-76 Median Age (UK) 55 Median Age (Spain) 61 | Community | To examine the perspectives and preferences of individuals with no symptoms of dementia, people with dementia, and caregivers on the communication of Alzheimer's dementia risk | Qualitative | NR | FGDs | NR | Views and expectations re learning risk status for DRR |
| Milne et al., 2018b | UK, ES | Adults participating in dementia related cohort studies 48 NR Median Age (UK) 55 Median Age (Spain) 61 | Community | To examine expectations of the main potential implications of learning genetic risk test results | Qualitative | NR | FGDs | NR | Views re: expectations for DRR of learning risk status |
| Nelis et al., 2018 | UK | Adults, participants in pragmatic goal setting for healthy ageing trial 75 51-84 68.2 (7.9) | Community | To evaluate the feasibility and implementation of a goal-setting intervention to promote healthy cognitive aging in later life | Mixed methods program evaluation | NR | SSIs, field notes | CA | Process evaluation re: DRR trial |
| Neville et al., 2013 | UK | Adults with MCI and their caregivers, participating in DRR intervention pilot 63 (MCI and caregivers)  NR NR | Specialist memory clinic | To explore attitudes to diet and physical activity in people with MCI and gain an understanding of why people with MCI may have difficulty making lifestyle changes for cognition. To explore the format, acceptability and potential usefulness of piloted educational material in helping to promote behavior change in this group | Qualitative component of mixed methods design | NR | FGDs, SIs (telephone) and field notes | TA | Views re: DRR and evaluation of DRR educational material |
| O'Brien et al., 2013 | US, UK | Older adults participating in preliminary phases of trial of online brain-exercise for prevention of dementia or cognitive decline 34 60-64 (Median age range) | NR, research trial | To understand underlying motivational and de-motivational factors influencing seniors' engagement with mobile brain exercise software. | Qualitative | NR | FGDs | CA | Views on motivational and de-motivational factors of brain exercises and evaluation views of a brain exercise software |
| Pace et al., 2019 | CA | Middle-aged and older adults and family caregivers in Inuit community 14 NR NR | Community | To consider the role of culture and the natural environment in promoting behaviors that may contribute to healthy aging and be protective against cognitive decline | Participatory research, photovoice approach | A critical interpretive theoretical framework (Scheper-Hughes, 1990) | IDIs | Phenomenological TA | Views on the role of culture and natural environment in DRR |
| Price et al., 2011 | US | Older adults identifying as African American or White 65 NR 70.1 (3.1) | Community | To examine older adults' perceptions of physical activity and cognitive health | Qualitative | Communication theories: elaboration likelihood model (Petty and Cacioppo, 1986); extended parallel processing model (Witte and Allen, 2000) | FGDs | Open coding process; Axial coding process; Constant comparison | Views on relationship between physical activity and cognitive health and preferences for messaging |
| Robinson et al., 2018 | UK | Middle-aged and older adults 31 NR NR | Community | To critically explore the views of the public about the acceptability and feasibility of proactive approaches to identification of people at high risk of dementia | Qualitative | NR | Task groups | TA | Views on disclosure of risk status for DRR |
| Thorgersen-Ntoumani et al., 2018 | AU | Older adults with subjective memory complaints and spouses of people with SCD 30 60-93 72.6 (8.2) | Community | To explore how older adults with subjective memory complaints view social dance as a potential intervention to improve cognitive functioning | NR | NR | SSIs | TA | Views re: perceived barriers, benefits and facilitators to a proposed program of social dance to improve cognitive function |
| Traphagan et al., 1998 | JP | Older adults, living in rural hamlet Approximately 50 NR NR | Community | To examine the meaning and experience of senility (boke) in Japan | Ethnography | NR | Ethnogra-phic interviews | NR | Views: re preventing senility |
| Walker et al., 2014 | AU | Older adults, carers of people with dementia, participating in computer-based brain training trial 10 65-81 NR | Community | To explore the experience of older Australians completing a computer-based brain training program | Mixed methods program evaluation | NR | SSIs, weekly journal | Phenomenological approach | Process evaluation re: DRR intervention |
| Watson et al., 2019 | UK | Middle-aged and older adults 10 53-75 63.7 (NR) | Community | To explore what matters when developing new treatments to prevent AD and how trade-offs are made between risks and benefits | NR | NR | FGDs | NR | Views re: outcomes that matter, risk disclosure, preferences for DRR |
| Wiese et al., 2018 | US | Older adults, living in rural community, caregivers of people with AD 20 NR 76.86 (7.89) | Community | To gain greater understanding of rural residents' perspectives about AD | Qualitative component of mixed methods | Nursing as caring (Boykin and Schoenhofer, 2001) | SSIs | Open coding | Views re: DRR |
| Wilcox et al., 2009 | US | Older adults identifying as African American, Chinese American, Vietnamese American, Hispanic, Non-Hispanic White and American Indian  396 NR 71.0 (9.3) | Community | To examine older adults' perceptions of the link between physical activity and nutrition to the maintenance of cognitive health | Qualitative | NR | FGDs | Axial coding; constant comparison | Views: re: physical activity and diet for DRR |
| Wu et al., 2009 | US | Older adults 67 NR NR | Community | To examine gender differences in views about cognitive health and behaviors that have been associated with its maintenance, focusing on older adults living in rural areas | Qualitative | NR | FGDs | NR | Views re: cognitive health and health behaviors that may promote it, and gender related differences. |
| Zallen, 2018 | US | Adults, aware of genetic status as heterozygote or homozygote for APOE e4 26 31-78 52 (NR) | Community | To explore reactions of individuals who have undergone APOE testing and discovered that they are at higher risk for AD | NR | NR | SSIs | Qualitative-description approach | Views re: impact of knowing genetic risk status on DRR |
| **Additional Studies from Updated Searches** | | | | | | | | | |
| Akenine et al., 2020 | FI, FR, NL | Older adults participating in an ehealth intervention trial  44  NR  71.7 (approx.) | Community | To explore the attitudes of older adults at increased risk of CVD and dementia regarding engagement in eHealth self-management prevention programmes, and to describe the facilitators and barriers | Qualitative, based on grounded theory | NR | FGDs | GT principles | Views re: DRR and |
| Bacsu et al., 2020 | CA | Older adults in rural community  42  60-87  NR | Community | To explore activities that older adults identify as being important to supporting cognitive health | Ethnography | NR | Observation  SSIs | TA | Views re: activities contributing to DRR |
| Bosco et al., 2020 | UK | Older adults participating in online survey of views  653  NR  64.1 (8.3) | Community | To explore willingness to change lifestyle and behavioural factors to reduce future risk of dementia in people aged 50 or older | Qualitative component of mixed methods design | NR | Web-based open form survey | CA | Views and experiences re: changing behaviour for DRR |
| Cooper et al., 2021 | UK | Older adults with SCD or MCI, participating in technology-based multi-domain intervention  10  NR  74.3 (8.6) | Community | To explore how older people with memory concerns experienced and used a video-call, group-based cognitive well-being intervention | Qualitative component of mixed methods design | Behaviour Change Wheel | SSIs and FGDs | CA and TA | Evaluation of dementia risk reduction intervention |
| Halloway et al., 2020 | US | Older women with CVD risk factors for dementia, participating in physical activity intervention.  8  NR  NR | Community | To test the feasibility of an evidence-based lifestyle physical activity intervention for use with older community women at risk for memory loss due to CVD | Qualitative component of mixed methods design | SCT | FGDs | Thematic CA | Evaluation of dementia risk reduction intervention |
| Largent et al., 2020 | US | Older adults participating in a longitudinal study of the consequences of amyloid PET scan results  80  65-85  NR | Community | To understand the effects of biomarker disclosure on AD risk-reduction behaviours | Qualitative | NR | SSIs | TA | Views on dementia risk reduction behaviour change intentions and actions following disclosure of risk status. |
| McGrattan et al., 2021 | UK | Older adults with MCI, participating in a lifestyle education intervention to prevent dementia  8  77.0 (NR) | Community | To evaluate the feasibility of a Mediterranean diet and lifestyle education intervention | Qualitative component of mixed methods study | TDF | SIs | FA | Evaluation of materials for diet and lifestyle education intervention for dementia risk reduction. |
| Swindells et al., 2020 | UK | Adults without any professional experience or expertise in dementia  15  25-74  NR | Community | To examine the mental models on dementia risk held by laypeople of all age groups to identify gaps and misconceptions that may be targeted in future public health communication. | Mental models approach | Mental models (Morgan et al., 2002) | SSIs | TA | Views re: dementia risk reduction |
| Wesselman et al., 2020 | NL, DE | Adults with SCD, participating in pilot trial of online DRR intervention  30  NR  NR | Community | To gain insight into user experiences of online program for brain health | Qualitative | NR | SSIs | NR | Evaluation of DRR intervention |

*NR, not reported; US, United States; FI, Finland; FR, France; NL, The Netherlands; UK, United Kingdom; CA, Canada; DE, Germany; AU, Australia; KR, Korea; ES, Spain; BD, Belgium; JP, Japan; MCI, Mild Cognitive Impairment; SCD, Subjective Cognitive Decline; AD Alzheimer’s disease; ISSI, individual semi-structured interview; FGD, focus group discussion; SSGDs, semi-structured group discussion; SI, structured (individual) interviews; IDI, individual (in-depth) interview; GT, grounded theory; TA, thematic analysis; CA, content analysis; FA, framework analysis; APOE, Apolipoprotein E gene.*
