# Supplementary material for: General population perspectives of dementia risk reduction and the implications for intervention: A systematic review and thematic synthesis of qualitative evidence
Source: PLoS One. 2021 Sep 17;16(9):e0257540. doi: 10.1371/journal.pone.0257540 (PMC8448319; doi:10.1371/journal.pone.0257540)
Supplement: S2 Table — (DOCX) [file pone.0257540.s005.docx]

| **Theme** | **Contributing Quotes and Author Interpretations** |
| --- | --- |
| ***Knowledge and Understanding*** |  |
| Understanding dementia risk reduction | *'Because you could probably do something with nutrition, you could probably do something with medications, you could probably do something with your physical self, be it exercise, crossword puzzles, whatever you need to do.’*  *’I go three nights a week to a class and last night we learned a 64-step glider. So, you're not only exercising your body, you're exercising your mind because you have to remember.’*  *‘You know, you have to eat the right kind of food, and be exercising, you know, working, read a lot and, you know, just - but I think eating play a lot in that, what you eat.’*  *‘Mentally active. This includes playing cards, memorizing things, being exposed to new things. A little bit of everything.’*  ‘Men reported engaging in activities such as working cleaning and hunting for physical exercise.’  ‘Very common were more generic references to 'keeping active', 'staying busy' and 'exercising'.  *‘It’s always learning.’*  *‘We are like walking computers. If we don’t put information into our brain, it becomes void and useless to us and others.’*  *‘Brain is only healthy by the information it’s given.’*  *‘You are continuing learning, open-minded and you observe things, the past and the present and what the future might be.’*  *‘Even playing poker every day is good for keeping a healthy mind.’*  *‘Play Mah Jong to keep your brain working.’*  *‘Stay active, do puzzles, do things that make the brain work’.*  *‘Especially mental [activities] and so, like, crossword puzzles, require you, learning something new.’*  *‘I just hear everybody saying, you know, they're healthy to do - puzzles.’*  *‘To keep our brain healthy, that means you need to remember where you pick up and put down things. Everything we do, we must pay attention to our memory. You must remember things, don't just leave things around and walk away. After a while, you won't be able to remember it. That is, if you don't try to memorize things, it does not keep the brain healthy. If I put something down, I must remember where I put it. I must remember this. This is how I train my brain to be healthy.’*  *‘Minds always going.’*  *‘I started doing crosswords and playing memory games.’*  *‘Keeping your brain active.’*  *‘Doing complex mental activities.’*  *‘Challenge your brain with something new and learn and develop new skills.’*  *‘I read a lot in my book group ... I do keep my brain, you know, as active as I can’*  ‘*I do word puzzles.’*  *‘There is this other thing about keeping your mental activity up as you get older, you know, stimulate the brain; do crossword puzzles, learn new things and keep your brain working. ‘*  *‘It means you’re gonna have to work hard to keep from getting like that and read and do things to keep your mind busy.’*  *‘[Reading is] exercise.’*  *‘[Reading is] training.’*  *‘[Reading is] activating your brain.’*  *‘Keep on working. Thinking longer just keeps your brain sharp.’*  *‘If one does not use his brain ... does not read every day, the brain will slowly deteriorate.’*  *‘He said reading the Bible has been very important to him in the last 23 years. He feels that reading the Bible has helped to cultivate his memory.’*  *‘Two novels a day, I just read, read, read, and my kids say 'how do you do it?' and I say it takes me away.’*  *‘Well, basically exercise … making sure that your arteries are clear of plaque.’*  *‘You must try to walk faster at times, so that you breathe faster, and take in more oxygen. Then you have a clear mind. If you have more oxygen, you are bound to have a clearer mind.’*  *‘I stay active, I exercise 3,4, maybe sometimes more times a week.’*  *‘[Exercise] helps get the blood flowing to the brain.’*  *‘Just recently, I read something about conductors. They live longer because of the exercise that they do … You ought to see me going around the house now. Every time I hear music, I'm conducting.’*  *‘Oh yeah, I know about that. Keep your body moving.’*  *‘He's very - he's very physical. He's always doing something. He's not the one to sit around and not do anything … anything he can go to when he's home, he does.’*  *‘I think nutrition has a lot to do with - brain has to have the right nutrients to be able to support you.’*  *‘Having healthy organic food, taking dementia–related medications, or considering changes of the surrounding environment.’*  *‘In my understanding diet has a place.’*  *‘Well, I just think if you feed your brain with the food it improves your mind and your ability.’*  *‘There are some foods that you eat for your brain.’*  *‘Quit using aluminum pots and pans, avoid aluminum in skin care and deodorant products.’*  *‘Eliminate canned foods due to link now realised between canned foods and AD.’*  *‘I was reading about AD and it said that sometimes its the preservatives in your meat, and I thought well we're going to raise beef ... that was just something that was very important to me. That way we know what's going into them and that they aren't being filled with preservatives and all the crap that they put in the meat.’*  *‘We have our own garden and eat stuff that way so I can't say that they're necessarily be chemicals ... it's more natural or whatever, our own stuff.’*  *‘You have to think about the way we fix our foods.’*  *‘Fruit represents health to me. So that's why I took that [photograph]... It leads to not only brain health but every other kind of health, I suppose.’*  *‘I don't eat a lot of salt.’*  *‘They [the media] say Japanese people live longer because they eat a lot of fish.’*  *‘[The media] always talks about eating fish.’*  *‘We attempted to change things like ... having healthy organic foods.’*  *‘There's always the diet thing and I do somewhat watch that because I'm certainly aware of the diet connections. There's a lot more that people don't understand about cholesterol as well.’*  *‘Specifically, an anti-inflammatory diet, such as the Mediterranean style of eating.’*  *‘Intermittent fasting.’*  *‘Eating leafy greens every day.’*  *‘I think it's all that crap we are eatin' these days; all the junk; that is what is doin' us in. If we stopped and went back to farmin', that would decrease your chances.’*  *‘Put the proper foods in your body and the right amount and if you're a little bit overweight you might pay close attention to that too.’*  *‘They got a lot of fats in your vegetables and stuff like that; you've got to stay away from it.’*  *‘We have a seafood market not too far from us and we order and they deliver it. We order at least three times a week, we'll get that fish.’*  *‘Blueberries are good for the brain.’*  *‘Pineapple is good for memory.’*  *‘Well I just think if you feed your brain with the food it improves your mind and your ability.’*  *‘And I truly believe that eating lots of fruits and vegetables helps.’*  *‘We also take vitamins and supplements.’*  *‘Use CAM: take vitamins, supplements, herbs, tinctures.’*  *‘Wildroot was supposed to be a cure for everything and it's common here.’*  *‘I drink it [wildroot] in tea, it doesn't taste too good. But it's like my daily medicine; I'm too old to go out and get it, but maybe it helped me; I'm still goin' strong- HA!’*  *‘We attempted to change things like ... having healthy organic foods, taking dementia-related medications, or considering changes of the surrounding environment.’*  *‘You have to think about the way we fix our foods.’*  *‘I think when people are working, they have a healthy brain.’*  *‘[Work] provides stimulus for my brain and added physical therapy for my body.’*  *‘You don't literally have to be working, but as long as you're moving, doing something. Because I don't work anymore, but I'm still active and you are too.’*  *‘I think you have to keep busy – busier. I see when I go to the hospital, you know, people are sitting there and doing nothing.’*  *‘I never worry about yesterday if I can help it, however it does creep in as you get older because your friends die and go away and if you haven't taken the time to make new friends you are left like an island so that's why you have to keep moving, keep getting involved.’*  *‘Play with the grandchildren, or you know, communicate with the younger generation.’*  *‘Being a part of social groups and interacting with others around you [is important] ... interacting with anyone willing to listen to you in a friendly way.’*  *‘Being involved with children helps you too, keeps you on your toes.’*  *‘[Children] are like sponges. They can catch on to something quick, so if you interact with them and ask them questions - you'll be surprised what you learn.’*  *‘I think it's like, don't be left alone, like, have family around, and be able to talk all the time.’*  *"We're talking about old people. We're talking about memory and, and, to tie it in with the social aspect of this and how important it is [for memory]. We affect each other, I mean, whether we want to or not. We, you know, whether we agree with somebody or disagree with somebody, we're going to affect each other".*  *‘If you stay indoors and don’t get out, then your mind will slow down. When you have time, even if you just chat with your friends, you will be more open, and able to keep your mind sharp. Your mind will be more sensitive to things.’*  *‘At home you're just crocheting by yourself, but when you're in a group crocheting, you're talking, you know, your brain is more active.’*  *‘I think the family is what matters, if they're happy with one another, the children are successful or obeying us, then, that will make our brains sharp.’*  *‘But, it's the social, you know, you got to have interaction with people. And if you don't you just sit in limbo all the time.’*  *‘Develop healthy interpersonal skills.’*  *‘Have stable, loving relationships with family and friends.’*  *‘[In order to avoid boke], living alone or as an elderly couple is not so good, it's best to live with family.’*  *‘Music, either music or tape with reading and writing and playing cards, and you know, just-just busy. What's up with things on video and watch and learn and just, I think you have to keep busy - busier. I know our elders are busy, but to be busy on puzzles or just to keep going.’*  *‘Like any other kind of health, good and poor, brains get tired too, need time to rest your brain, need to figure out how to cut it off from things that deplete energy of your brain.’*  *‘As long as you are satisfied with life, you can have brain health.’*  *‘Everyday humor and warmth [are important].’*  *‘I have some things that I do that's personal and one of them is meditate and I use this as my meditation and it's good for you, any of you that want to. Let this mind be in me that was also in Christ Jesus. He never lost his mind and I will not lose mine. I promise I will not lose my mind because that's what I quote every morning.’ ‘Anything bothersome can be put aside; happy and smiling means living longer, healthy, a lively brain.’*  *‘So don't carry burdens on your mind. Keep healthy life habits. Let go of mental burdens. Thus, one can live a healthy life and keep a sharp mind.’*  *‘When you are happy, your brain will be good.’*  *‘Reducing stress and being part of something bigger than yourself, through things like having a pet, believing in a higher cause, and volunteering.’*  *‘Get your hearing checked and wear a hearing aid if necessary. Loss of hearing can cause portions of your brain to lose function.’*  *‘I went to the drugstore to ask whether they had something to improve my memory.’*  *‘In addition, we went to a community health centre to take some blood tests.’*  *‘There is a good medication for brain circulation, which is a cerebrovascular improver.’*  *‘Most Chinese think it's important to keep a healthy mind, so they would do their best to keep themselves healthy. They do it through all aspects including diet, sleep and exercise.’*  *‘First in staying sharp is exercising. The second is diet. And the third is being active in society. Go often, participate often then naturally your spirit will rise and reduce all of the other [negatives].’*  *‘Brain health is such a wide spectrum, not just A, B, C, D.’*  *‘Keep your mind busy and your body, mind and soul busy and no-no chemicals, you know, eat well, sleep- sleep good, to bed early, no gambling.’*  *‘Reducing screen time and spending time outside and having sex frequently and running steep hills.’*  *‘You gotta exercise, not smoke, eat right - you know, avoid the sugar, all that stuff.’*  *‘Gaining brain health starts in the mother's womb. In order for it to develop we must be introduced to books and reading at an early age and this must remain constant throughout our lives.’*  *‘[Health for the older citizen] should be from the cradle to the grave.’*  *‘I do a lot of things.’*  *‘I think less strain, better food and more recreation keep you from losing your mind [cultural definition of cognitive decline] and not having a lot of stress. We would go to a polka fest for a weekend deal. They were relaxing. You could forget about your problems when you were gone, even though they were still there when you got back.’*  *‘Just promote ... healthy lifestyles, healthy eating, you know, especially in church here.’*  *‘For me, participation in the hamlet ground golf team, reading out loud for at least 30 minutes a day and working in my garden and rice fields are ways I am trying to avoid becoming boke.’*  *‘This is an issue of all aspects ... diet, exercise. In China, we have a proverb called ‘Three Self's’ – ‘Self-content, self-belief, and self-enjoyment.’… Then there is also the 'Three active’:- ‘Mentally active’ - This includes playing cards, memorizing things, being exposed to new things ... a little bit of everything. Physically active - just like this Madame, who often does Tai Chi and sword exercise, and dancing... and socially active - one must socialise. However, a Chinese proverb says too much of anything is not good. Exercise is good, but over-exercising is not good either. So I always emphasise balance ... balance.’*  *‘I'm in pretty good shape because I'm a volunteer fire fighter.’*  *‘Three selves - self-content, self-belief, and self-enjoyment. One must be content in all aspects - family, life and one's own age.’*  *‘I dunno, exercise might help, and if you call workin’ in the coal mines all your life exercise, I did.’*  *‘With myself, I see that in order to keep it sharp, we have to work where we have to think. For example, we read a book, we read a story. When we read it like that, it's like we're training our memory so that we can stay sharp.’*  *‘You should read a book, then close it. Then, you think about how many characters there were. See if you can recall all of the characters. That is one way to train the memory, that is to say a healthier and sharper mind.’*  *‘We go to the bank. We deposit money. We withdraw money. We get to know the teller and that environment. We won't be stupefied. But if we deposit straight into the bank and withdraw at the ATM without interacting with the teller or the animated environment at the bank then we become stupefied, like a lost person in the middle of town.’*  *‘I do a lot of reading at home and I have my favourite games on the computer that I play when I can't sleep, things like that challenge me. I think that's what's hopefully going to see me through.’*  *‘You pay attention that you do enough physical exercise. I know all the risk factors. I don't have high blood pressure or those kind of things. But I don't eat a lot of salt. So I incorporate it in my life.’*  *‘My husband ... was a man who loved math. He made math out of everything. He figured out how many seconds he had to live if he lived so long, how many seconds. On the 4th May was his birthday. He was 89. We had a party for him and he died on the 20th. But that mathematical brain - he never, he didn't show any sign of forgetting.’*  *‘Make an effort. Find newspapers. Read books, Interact socially. That's why, in all of my experiences, I've seen active saintly and temple people. All of them are socially active and are all 70 to 80 years old. Why? They have a sense of faith and they're active in the community.’*  *‘Well, I read that Ronald Regan had it, didn’t he, and Iris Murdoch – very well educated people – and I think you find that. Fred Fletcher from the Rotary Club, now he had it and he was a very bright chap.’*  *‘And feed your brain. I think you know, I've discovered that I don't have enough fish oil in my life or haven't been, so I started taking my fish oil again. And instead of taking what they prescribe, I find myself taking [more]. I need lots of fish and the more fish I have the better I feel and, the brighter my head is. So, and when I don't take it and there are times when I'll not take it for a week, I'm kind of, like a clock, you know, all of sudden it wounds down, so I get back on my fish oil and okay.’*  *‘Three meals a day and plenty of fruit and vegetables, he would be a veg man, lots of salads; it's definitely helped him out.’*  *‘1,000mg Vitamin E, I take one of them every day and my mind is developed considerably since I started taking them.’*  *‘My mother in law took the ginsa thing. She got it anyhow. But lots of people roun’ here took yellowroot growin up ... It's supposed to be good for a lot of stuff.’*  *‘My daddy lived to be 93 and it was because of his good work ethic. When he retired until he had his stroke he would plant a garden, slaughter hogs, cure country hams, make sausages, can. He would make us pick green beans, tomatoes and stuff. I'm convinced that's why he lived.’*  *‘We’ve used spring water all these years we've been here and I'm just fine. When you moved around years ago, you got [water from] different well from different places but the town use chlorine in the water. I'm sure there's something in the food too.’*  *‘You have to concentrate on it [games]. My brother-in-law's mother was in a rest home. She was like 96. Every day they went up and played dominos with her. I mean, she would beat the socks off of them - up until they knew when she was getting ready to die, because her mind started going. And it wasn't within two weeks, she was gone. So, you know, if you just keep active and make yourself concentrate on things. I think that's it.’*  *‘I'm sure there are things that we can do to improve our health as we grow older, you know, but I think for the previous generation, the generation before us, I think they did what they could to remain healthy, they worked hard, they were caring people and they worked harder physically than our generation did, you know, and today's generation as well, I mean, they were slaves, really. It was a hard life, you know, but they accepted it and I think it contributed to their well-being.’*  *‘Using one's hands is the key to avoiding becoming boke. Because I have always used my hands because of my work as a tailor, I have avoided becoming boke.’*  *‘As long as you exercise, you’ll be ok. My father doesn’t exercise. Now, he can’t even recognise his son.’*  *‘And, I just watch my mum here and I know she takes a lot of over-the-counter vitamins and things that helps her and I tell you she, she's sharp. I have to say she's sharp.’*  *‘I find that if I have a piece of work to do and I'm getting too bogged down in it, if I do something that's puzzle-based then I can come back and my mind is a bit fresher.*  ‘Creativity was viewed as a form of mental stimulation and a way of promoting and reflecting brain health, including music, poetry, drawing and painting.’  ‘Regardless of their perception of the utility of appropriate medication, participants indicated that seeking regular healthcare … as well as following physicians’ orders could facilitate DRR.’  ‘Social engagement and connections were viewed as important aspects of healthy brain aging.’  ‘In free-text responses, participants described the value of … *‘having regular check-ups with your doctor.’’*  ‘Others focused on volunteering as an opportunity for both social engagement and a way to feel productive, both of which were viewed as good for brain health.’  ‘Others focused on the importance of continuing to find ways to contribute and continuing to value older adults and their contributions.’  ‘Several participants discussed continued work engagement in regards to staying active and promoting brain health.’  ‘Community and civic engagement was suggested by all groups.’  ‘A White participant said connecting with younger relatives and telling them stories about one’s own childhood could help keep the mind sharp.’  ‘For some participants, exercise, specifically dance, represented an opportunity to prevent cognitive decline and, more specifically, to protect against dementia.’  ‘A wide range of specific physical activities and structured exercises were described, with walking, dancing and lifestyle activities most common.’  ‘Participants advocated for moderate use of and abstention from alcohol, widely recommended avoiding recreational drugs.’  ‘Taking dementia-related medications.’  ‘Younger participants suggested giving up smoking and drinking.’  ‘A wide range of specific physical activities and structured exercises were described, with walking, dancing and lifestyle activities most commonly.’  ‘Subtle differences were noted in the types of physical activities mentioned by racial/ethnic groups. African American (AA), white and Chinese participants mentioned the greatest number of PAs that promote brain health. Walking was mentioned less often by American Indians (AI) than other groups. AI did not consistently cite a preferred PA. In addition to listing many of the same activities as other ethnic groups, Chinese participants frequently discussed the importance of traditional Chinese exercises including Tai Chi, Qigong and Yoga.’  ‘The 'Labrador life' is perceived to provide the necessary supports for physical and mental health by providing opportunities for physical exertion and engagement (e.g., berry picking, shoveling snow, cutting firewood), nutrient-dense wild foods that contribute to a healthy diet (e.g., fish, berries, wild game) and engagement with the natural world, which is understood to support mental wellness.’  ‘Continued engagement with the land and a subsistence lifestyle remains a cornerstone of understandings of healthy aging and dementia prevention.’  ‘Less worry preserves cognitive health was mentioned by all groups.’  ‘The benefits of humor or optimism were mentioned in all groups. Related, laughter was mentioned repeatedly and encouraged by participants in many of the groups.  ‘Chinese participants mentioned dancing as a form of PA that could slow the development of Alzheimer’s disease.’  ‘Older participants suggested regular medical check-ups.’  ‘African Americans and American Indians did not mention hearing about the relationship between PA and brain health, some participants recalled media messages about brain exercises or puzzles, such as Scrabble or Sudoku.’  ‘Reading was the most frequently recommended mental exercise (all ethnic groups). Reading was also considered helpful for maintaining or improving memory. Participants suggested a variety of reading materials. Only American Indians mentioned reading on a computer.’  ‘Most groups described a variety of activities promoting other types of mental stimulation, including: using computers and learning a new language, solving arithmetic problems.’  ‘Most groups mentioned spending time with family and friends.’  ‘Intergenerational exchanges mentioned by most groups. Included the usefulness of passing on oral history or telling stories about one's own childhood to the younger generation.’  ‘Most groups (except Latino) talked about continuing to work as a way to maintain cognitive health.’  ‘Aging in place, with access to well-established social networks and familiar places, is also understood to have protective benefits related to cognition.’  ‘Seeing other people regularly was described as good for the brain and for mental wellness. Visiting and social gatherings were perceived to spur laughter, storytelling, and reminiscence, which were described as supportive of memory as well as the reinforcement of collective identity and shared experiences.’  ‘Less commonly mentioned examples of mental stimulation were continuing education, keeping up with current events, learning telephone numbers and reading brochures and the dictionary.’  ‘The possible association between happiness and promoting cognitive health was mentioned by all groups (especially Chinese and Vietnamese and rarely African Americans). Activities providing happiness included: singing and writing and reading, spending time with family, laughing, being spiritual and believing in God, and doing puzzles and games.’  ‘Puzzles and games were mentioned by all ethnic groups for promoting concentration and memory. Examples: Crossword puzzles, word searches, jumbles, jigsaw puzzles, scrabble, sudoku, bridge, dominos, mahjong, monopoly, jeopardy, family feud, bingo, chess, solitaire, and computer games.’  ‘11 out of 12 emphasised the importance of home-based cognitive training to maintain or improve level of cognitive functioning.’  ‘Increasing mental stimulation most commonly identified way to reduce one's risk of developing dementia (crosswords, sudoku, learning something new).’  ‘There was mention of staying mentally active to maintain brain and general health by six of the nine dyads. Activities that members of the dyads presented to help promote mental activity included involvement in weekly church activities, puzzles and playing cards. In three cases where care partners spoke about lack of their loved one's or their own mental activity, there was also the direct and indirect acknowledgement that promotion of brain or mental activity is good for one's health.’  ‘Activities such as reading, journaling and doing crossword and jigsaw puzzles were described as advantageous.’  ‘All participants endorsed a variety of cognitive activities, including reading, completing crossword and other puzzles and keeping busy, as strategies to maintain cognitive health.’  ‘Belief that the practice of learning and remembering dance sequences could help ‘ward off’ Alzheimer’s disease was shared by other participants.’  ‘Participants discussed brain health being reflected by and promoted by continued activity outside of the work setting.’  ‘Eating well included making healthy food selections and avoiding chemicals and preservatives. Seven of the nine dyads spoke about making or trying to make healthy food choices.’  ‘Others maintained that avoiding aluminum is essential to dementia prevention.’  ‘In addition to healthy food choices, the avoidance of chemicals and preservatives in food emerged.’  ‘Diet and nutrition were mentioned less frequently than physical activity. Two subthemes: the perceived role of a healthy diet on brain health and the characteristics of a diet that promote brain health.’  ‘Diet also helps keep the brain healthy was mentioned several times in each of the focus groups. Participants recognised that diet was one of several behaviour that influenced brain health. There were participants who were not convinced that diet could make a difference in brain health. Participants made more comments re: characteristics of diet that promoted brain health theme than any other. Limiting portion sizes; preparing foods in healthier ways; eating more fish, fruits, vegetables, low-fat foods, and chicken; and eating less red meat and chicken with the skin were associated with brain health.’  ‘The rural lifestyle protected health, and cultural health practices were based on readily accessible, natural resources that were trusted and believed to prevent disease and injury and cognitive decline by providing regular social visits with neighbours; a sense of life purpose from working on the farm; natural foods and water derived from the farm; and, stress management through dancing and playing cards.’  ‘Both men and women agreed that a healthy diet was important for aging well.’  ‘Participants also recalled media messages about protective effects of medication, vitamins and supplements.’  ‘Some also perceived value in alternative medicines to prevent dementia, such as taking herbal or vitamin supplements.’  ‘Multiple dietary supplements were also discussed.’  ‘In almost every FG, discussions of food types that promoted brain health included frequent mentions of dietary supplements and vitamins, such as multiple vitamins; vitamins C, B and D; calcium; omega 3; garlic; ginger root; fish oil tablets; ginkgo biloba; evening primrose oil; and garlic tablets.’  ‘Participants shared their perspective that brain health is diverse.’  ‘Participants view [brain health and its influences] as highly intertwined. The factors that promoted brain health also frequently reflected brain health.’  ‘Prevention strategies: included talking, eating healthy, getting sleep, being chemical-free, avoiding gambling, exercising the mind, body and spirit.’  ‘Other suggestions included increasing or keeping physically active, socialising and maintaining a good diet.’  ‘Most people hold at least two or three theories of causation at the same time.’  ‘Strategies related to dementia prevention are closely related to practices that are perceived to be supportive of good health over the life course.’  ‘Cognitive health is understood to be directly related to a combination of factors including engagement, activity, good health, a positive attitude and social connectedness, which includes challenging one's brain.’  ‘A prominent theme across groups was the idea that although PA is important for brain health, it is just one component of brain health and a generally healthy lifestyle. Other important components cited by participants included good nutrition, social engagement, psychological stability, remaining busy and active, ensuring adequate rest and performing mentally challenging activities.’  ‘The participants have actively tried to practice a variety of healthier lifestyle behaviours considered useful for dementia prevention. These include: diet, dietary supplements, physical activity, leisure activities, good sleep, vaccinations, regular health checkups, medication adherence.’  ‘Participants expressed a preference for exhausting means other than medications for dementia prevention.’  ‘Another care partner spoke to the maintenance of the family garden. The dyad's choice to raise their own livestock or maintain a garden demonstrates the efforts undertaken to promote, what they consider to be, an important feature of healthy eating.’  ‘Others discussed the need to take care of the brain much like you would a plant, providing nutrients and sunlight. Nourishing the brain involved findings ways to relax, recharge and focus on oneself.’ |
| Need for information | *‘I knew it was good for you, but I didn’t connect it to memory.’*  *‘I think it is helpful for old people to learn health tips on how aging people can stay healthy. It’s important to learn what we’ve talked about just now. It’s terrible if people don’t want to learn. If they don’t learn, they’ll be ignorant.’*  *‘Needs to be more specific, for example, what is a small portion of egg.’*  *‘I don't want to know that I might be going to get it. I don't mind being told that 'if you do this, this and this you're less likely to have it' just as with heart disease or anything like that.’*  ‘The main barrier for behavioural and lifestyle change was the participants’ lack of knowledge.*’*  *‘Due to my poor health and my lack of medical knowledge, I’m relying on doctors, of course.’*  *‘Can I ask you a silly question to start with? What is dementia?’*  *‘I’m not sure about evidence or lack of it, this is the first I’ve heard of this potential preventive therapy.’*  *‘I've never heard of brain food that supposed to work.’*  *‘I don’t know that it’s widely publicized.’*  *‘Well, I’ve heard that fish is good for you … but only in the context that it’s good for you … I would take them [supplements] … more so for like joint and things.’*  *‘I realised that leisure isn't just for fun, that there are benefits; it keeps everything balanced. Leisure activities for brain health ... was such a surprise; something I hadn't considered but of course it all makes sense.’*  *‘I eat healthily already … need some new info, everyone knows what to do to be healthy.’*  *‘Outline exactly what [people with MCI] need to do.’*  *‘Explain to them, for example, eating fish is better than eating meat. Vegetables are better than meat.’*  *‘If you told me that I was at a high risk, I would certainly want to have, um, advice. How do I go on from here, what can I do to prevent it from getting any worse, or developing into a full-scale thing?’*  *‘It [information] was too general.’*  *‘More tips on how to keep the brain active.’*  *‘Advice on physical activity was not specific enough for older adults.’*  *‘You tell a person something, you left half of it out and don’t tell them all of it, then there ain’t really nothing there.’*  *‘We learned a lot about memory strategies. It was very helpful; like a librarian, we should always look for new information.’*  *‘Giving them all [elders] education, giving them what helps to keep memory alive.’*  *‘It is a thing that can happen to any of us so I’m paying a lot of attention to dementia and gathering information about it.’*  *‘Educate oneself-become familiar with research.’*  *‘I think in order to keep one healthy and sharp, it's important to have a type of meeting like this or get everybody to discuss health. Invite doctors, medical specialists to give speech(es) so we can broaden our knowledge.’*  *‘We expected that we would find out how we were doing, intellectually [as well as physically].* *I was rather interested in that. I wanted to know how I was doing.’*  *‘I was interested in the study and the information it may provide me concerning my health.’*  *‘I would want to know [risk status] and then if I could do anything myself to improve my memory, that would help.’*  *‘It’s a very specific platform. I wouldn’t expect information about numerous things but maybe some basic health information.’*  *‘Additional content is always good. I often use links.’*  *‘Too much focus on diet and not enough on other things related to memory.’*  *‘There’s a general advice there that’s not specific to dementia … there’s a generic thing going on about ‘well, if you want to keep your heart going, you need to give up smoking’. It’s the same things. So, maybe we need to actually pull dementia or the risks of dementia into that general health and well-being better than perhaps we’re currently doing’.*  *‘Explain to them, encourage people 50 years old and up to exercise, avoid certain foods, and take vitamins.’*  *‘[Including information from studies to show] there's some medical backing.’*  *‘I wouldn’t want to be bombarded with information all the time about it.’*  *‘I think I'd rather not know. If I had something that was fixable, I'd want to know and get it fixed, but this is your unknown.’*  ‘Perceived susceptibility. Not everyone with a rational response to perceived susceptibility calculated their chance of developing dementia correctly because they had incorrect information’.  ‘Most participants across groups were hesitant to share an exact amount [of physical activity] needed, noting the amount ‘depends on the individual’.’  ‘Several indicated that they were unaware that evidence regarding the role of antihypertensive medication and dementia prevention existed, or that they had not heard the specifics of the evidence.’  ‘[in an intervention] should include information on other possible factors associated with brain health, such as dietary supplement use and brain training.’  ‘High level of satisfaction with program. Information provided by expert therapists across different health and lifestyle domains of value.’  ‘Participants provided specific examples of important diet and lifestyle information that should be explained to others regarding brain health*.* Tailoring educational information was important to Chinese participants.’  ‘No one remembered receiving any information on diet and lifestyle in relation to their memory problems.’  ‘Only a few people had ever heard of the Mediterranean diet and no one had associated it with memory.’  ‘Caregivers suggested EM could be improved by more specific details such as lists of PA suitable for the less physically able.’  ‘In free-text responses, participants described the value of … *‘learn[ing] about advances in biomedical research.’’*  ‘MCI patients lacked awareness of the lifestyle cognition link.’  ‘Several indicated that they were unaware that evidence regarding the role of antihypertensive medication and dementia prevention existed, or that they had not heard the specifics of the evidence.’  ‘The main barrier for behavioural and lifestyle change was the participants’ lack of knowledge.’  ‘A wide range of physical activity frequency and duration recommendations were provided. The duration most often given by participants was 30 minutes. The frequency varied mostly between 3 and 7 days per week.’  ‘Walking was most frequently cited as a recommended PA, but participants did not know the recommended frequency, duration and intensity.’  ‘Views about what was an adequate or recommended amount of PA varied widely across participants.’  ‘Some participants valued general information on risk reduction but not detailed information about their personal risk of developing dementia. In light of varied individual preferences one participant suggested that individuals should be provided with a range of options.’  ‘Some participants expressed lack of knowledge about the strength of the evidence for specific prevention approaches.’  ‘[They had] never thought about it [physical activity] like that. [They thought about] heart benefit or health maintenance.’  ‘Participants from each race or gender group said they had not heard of the association [between physical activity and cognitive health] and that there is more information about other diseases such as high blood pressure, cholesterol and heart health.’  ‘Main reactions to being told 'scientific research suggests being physically active may help people keep their brains healthy and prevent or delay AD and dementia': … more information is needed.’  ‘Some participants suggested the platform could serve as a jumping off point for gathering information on brain training, cognitive health and similar issues. Also, links to related sites were regarded as very useful.’  ‘There is a need to educate First Nation Elders about 'what helps to keep memory alive.'  ‘Participants were sensitive to dementia-related information and actively collected the necessary information about dementia prevention.’  ‘The importance of help with interpreting information about risk and how to act on it were stressed.’  ‘Participants responded favourably to the positive and useful information about dementia reported across various media.’  ‘Most participants interested or willing to learn genetic or biomarker results.’  ‘Many were keen to learn more about the link between diet and dementia.’  ‘Throughout most of the interviews, there was a … willingness to be informed.’ |
| Education empowers choice and behaviour change. | *‘I have changed completely [in response to learning risk status], it has been very positive. I am healthier … I have sorted out my diet … and I’m much sharper than I was.’*  *‘If Elders have this knowledge, they will be better prepared to maintain their brain health.’*  *‘If they tell you, if you want to know, they have to put the cards on the table and say ‘you are in this situation, but have this, this and this you can do’.’*  ‘The main motivators for changing health behaviour and lifestyle for DRR were education.’  ‘Participants indicated that they had made positive changes in health behaviours as a result of heightened sensitivity to brain health.’  *‘[Following education I have] more awareness of food intake.’*  *‘And if there was something that they came up with that said, well, now, you know if you do this, maybe there would be a chance that you could reduce your possibility of having Alzheimer’s disease. By all means, you’d try it.’*  *‘[in response to knowing genetic risk] You would behave differently … with food, drink or habits, to walk more, to do more sport.’*  *‘Now more mindful of need to take care of self.’*  *‘In the end I’m glad I did it [learned genetic risk status] … And, yes, I’m glad I know because I think I am doing things that I might not do.’*  *‘So for me it [learning biomarker status] reinforced my belief that whatever it is that I'm continuing to do in my life, or whatever I'm doing in terms of trying to take care of my brain, I should continue to do it as much as possible.’*  *‘[After education] I use 75% of the food recommended in the five sessions.’*  *‘[After education I am] Using more oil, will work on using more grains, pulses, legumes and I will keep my 5 booklets and refer to them to enable me to correct my diet.’*  *‘[After education I] increased oil intake slightly. Stopping margarine, starting olive oil, increasing intake of spinach, salad and nuts.’*  *‘[After education] I cook with olive oil more, less sugar’*  *‘I clicked on it and I was APOE e4/e4. And … I kind of expected [it] to some degree. It still kind of sucked, but I took it a little more in stride, as in, ‘okay, what can I do?’ … You know I was almost enthusiastic … I think in a way it was almost like, ‘I can use this as a tool to redefine myself’.’*  *‘I think if it is clearly defined which activities contribute towards preventing it, I would have no trouble at all giving up the ones we are at and adopting the one we ought.’*  *‘I know what a healthy diet is. Not that I always do what's best for my health, but I do know what it is … There was no advice at all that was useful to me. They were all things that I already knew.’*  *‘[The influence of the nurse] was only indirect, because I knew it already. No, the conversations were not the main reason. I know we talked about it, and I took note of it, but I knew it already.’*  *‘It made me aware of physical and mental issues and gave me a chance to rectify them in a different way to one I would have chosen.’*  *‘[Mentioning Alzheimer’s disease] would grab their attention.’*  *‘Education is the only way … Increase understanding, not just blasting people with this is what you should do.’*  *‘A wake-up call.’*  *‘Information is good, it puts the problem on the table, if you do not have more risk, great, and if you do then you can remedy it.’*  *‘I probably will still never buy [a tablet]. But at least, I’d like to know how these things work.’*  *‘I think information on diet and exercise, it would be good for them to encourage them.’*  *‘It has made me think about what I eat. I will try and take on board the information.’*  *‘You can choose to ignore it, but you’ve been given the information and it’s your choice what you do with it.’*  *‘Education and having more information available that increases people's awareness of and understanding of dementia, rather than simply telling people what they have to do, would motivate people to change.’*  *‘I don’t think we necessarily have to enforce people to make, to go a certain route, but to have the information that you’ve got a predisposition towards something, well OK, then I may choose – or I may not – but I may choose to change my diet, to take more exercise, things that will actually potentially help the situation, may not, but that’s my choice.’*  ‘Perceived barriers: Most commonly perceived barrier to behavioural and lifestyle changes was the lack of knowledge about dementia.’  ‘Facilitators of change appeared to be an increasing awareness of how the targeted lifestyle changes might improve their well-being, … and awareness of diet being an  active choice.’  ‘Genetic testing prompted few changes in lifestyle.’  ‘The central reason that respondents were interested in knowing their AD risk status [was] that it might enable them to act to reduce this risk.’  ‘Hoping to prevent AD part of central construct identified of planning for the future as reason for learning about genetic susceptibility.’  ‘Participants’ expectations that risk information should be accompanied by information about risk reduction reflected the central reason that respondents were interested in knowing their AD risk status, that it might enable them to act to reduce this risk.’  ‘Several participants reported groups increased awareness of about heathy diet and impact on memory.’  ‘They stressed that future communication should be sure to say PA 'may' help prevent or delay cognitive decline.’  ‘Most said that recommending a certain amount of PA was inappropriate as everyone is at different levels of ability; instead, they suggested messages recommend people do as much as they can.’  ‘Participants were interested in learning their risk of developing AD primarily because of the perceived personal utility of the information.’  ‘Participants acknowledged that a significant limitation of proactive approaches was the fact that information did not automatically result in behaviour change.’  ‘Regardless of whether or not individuals chose to act on risk information, some participants felt that providing such information increased individual choice.’  ‘Most participants thought the educational material would be very helpful or helpful in changing their diet.’  ‘5 patients reported pre-clinical testing offers a benefit of prompting lifestyle change to improve.’  ‘Participants had positive attitudes about learning the application to increase walking and conversational reminiscence, but this did not necessarily influence everyone's intent to use the technology.’  ‘5 participants indicated that they had made positive changes in health behaviours as a result of heightened sensitivity to brain health.’  ‘50% indicated that they were more involved with leisure activities and had used information from sessions.’  ‘The only personal experience that was identified as influencing response to the groups was existing knowledge.’  ‘Two participants indicated that the provision of strong supporting evidence related to the efficacy of computer-based cognitive training would be an effective motivator to complete the training.’  ‘A participant with MCI reported increased awareness of the importance of variation in diet.’  ‘Participants demonstrated increased knowledge of proactive lifestyle factors that they could use to reduce the risk of future memory loss.’  ‘Motivating other older adults through education and increased knowledge was suggested only by Chinese and Vietnamese participants.’  ‘Reinforced participant choices related to lifestyle.’  ‘Nearly all participants indicated that they would have made a lifestyle change had their scan results been elevated, including changing diet (n = 25); exercise (n = 24).’  ‘Participants emphasised the likelihood that the identification of elevated risk would encourage and enable those at risk to take action to reduce it.’  ‘Some participants were more skeptical of risk reduction. They emphasised that much information is already available about healthy lifestyle, and although many people do make lifestyle changes, it is not clear that such changes would be more likely to take place because of an increased risk of AD.’  ‘Participation in the trial also raised awareness of age related issues and the need to make lifestyle changes.’  ‘Nearly all said they have benefited [from learning risk status] in the long-term from lifestyle changes, often learned from online sources, that they subsequently made. In the months or years following testing, nearly all (23/26) came to the conclusion that they had benefited in the long-term. This includes the individual experiencing adverse reactions.’ |
| ***The DRR Value Proposition*** |  |
| The value of reducing dementia risk | *‘I am afraid of getting it ... one of my greatest fears is that I will get dementia. I think the fear of getting dementia is great for me. I am really afraid of not being able to look after myself.’*  *‘They [college students] think it’s never going to happen to me and I’m going to live forever and who cares what happens to me when I get to 65.’*  *‘Just functioning with ADLs, do you know? If you've no recollection that you've not washed yourself, you're likely to go out and have issues, do you know. So if you can remember what you have to do, if you can remember how to drive a car, then you can take your dog down for a nice walk on the beach.’*  *‘*Fear of developing dementia was the main motivator to lifestyle and behavioural changes.’  ‘Although most participants expressed concern about developing dementia, a few, primarily White men, said they were not concerned about dementia. These participants said they would rather deal with cognitive decline if and when it happens rather than worry about whether or not they will get it.’    *‘Whatever it is I can do, then I would be like, yes, just give me the treatment.’*  *‘Being boke is something that I think about and am trying hard to prevent.’*  *‘My thought at doing this was to know so that if something comes down the pipe, that I could take that could circumvent it or prevent it, that I would be the first in line. That was my premise.’*  *‘Anything I can do to prolong it [MCI] or make it easier, that's what I'm trying to do right.’*  *‘If there's medication which can slow it down, delay it or whatever and I could have that medication, I would be silly to not have it.’*  *‘Well, if there is a way to prevent it, sign me up.’*  *You don't want people to lose their independence. You know, I mean, my whole goal in life, and being in health care as long as I have, is to be independent, to be fully independent and able to take care of themselves.’*  *‘Being able to function on the computer … I mean, that’s my source of information, you know, I’d want to be able to keep that.’*  *‘I don't mind if I can't do a cryptic crossword, I would really mind if I couldn't jump in the car with my dog, drive him somewhere and go for a long, long walk in the morning.’*  *‘We don't have to lose our mind or ability to do things. We don't have to cease from our activities and the trees that are on each side have done just the opposite. In that sense, I'm thinking of brain health.’*  *‘Without our brain, we are nothing.’*  *‘But certainly 30% [chance of getting dementia], I would probably volunteer for any sort of new trial drug, and perhaps even less than 30%. It's just because the only thing that I feel defines me is my mind, my thinking process. And that is almost, when that starts to go, or to fade or to get worse … then I would do a lot to prevent that happening.’*  *‘I hope to God that I don't get it. If you know you got it, it must feel dreadful.’*  *‘If I could take a drug that meant I was less of a burden on [my family] for longer, I would absolutely sign up for that.’*  *‘There are ways to avoid becoming boke or to fight it and if you do not, that is embarrassing because you were too lazy to have avoided it.’*  *‘I don’t think it’s right that money is taken away from children and the like … to treat the elderly.’*  *‘Actually,* *I have this point of view. I absolutely hate sports and such matters. So I will not do it. I'll probably live for a few less years: so what.’*  *‘I just think general health rather than worrying about staving off a particular disease.’*  *‘I am worried about ageing, and especially about being dependent. I see my completely dependent mother and I am projecting myself.’*  *‘But I saw that [a family member with AD] and I'm, I'm determined that I never want me or any of my loved ones to be like that.’*  *‘I am concerned that I might develop the dementia that my mother had.’*  *‘Well, I remember grandmother was, I guess she would repeat herself, so after a while they would kind of just quit listening to her and talk, talk about her, and she'd be sitting there, but they would be talking about her like she wasn't even there. I wouldn't want to be treated like that. So, it's important to me to try to remember things and not repeat things.’*  *‘We see our future through our parents. I do not want to spend my old age like my parent, who is having such a miserable life and putting so many burdens onto her children. I'm worried.’*  ‘The purpose of and motivation toward improved lifestyle did not seem to be preventing CVD as such, but to improve both current and future health and quality of life.’  ‘The participants thought that the FMWD were spending old age without purpose and were unhappy. These participants hoped their old age would not become miserable, like that of the FMWD, and that they would not develop dementia. Sometimes, they said, they dreaded their old age and wondered whether they would experience the same fate as their FMWD.’  ‘After looking at dementia’s progression in family members, they realised the importance and necessity of prevention and saw an opportunity to change their life by correcting their lifestyle habits.’  ‘Others valued the possibility of reducing risk.’  ‘For the participants who considered dementia to be normal aging, it does not make sense to them to search for a cure.’  ‘This was linked to a reduced sense of value for older citizens in society and dulled expectations.’  ‘Reducing the risk of dementia and trying to change one's lifestyle from what it used to be in the past comprised part of the essential structure of the phenomenon of fear of losing self-identity.’  ‘Any chance of improvement or delay in deterioration seemed to merit participation.’  ‘Reactions to results were mitigated by daily events that take precedence over concern about the possibility of developing a disease much later in life.’  ‘Enjoying life recognised as an important aspect of everyday life and therefore an important measure of the effectiveness of treatment.’  ‘Outcomes that matter lie in five key domains in relation to what matters in everyday life: everyday functioning; relationships and social connections; enjoying life; sense of identity; alleviating symptoms.’  ‘Maintaining everyday function important to healthy volunteer participants.’  ‘For some, dementia and particularly loss of memory was seen as a problem worth trying to prevent, even if the chances of developing these were less than 30%.’  ‘Throughout most of the interviews, there was a tone of positivity, even hope, for finding treatment for AD.’  ‘Avoidance of burdening others by becoming boke is a central theme.’  ‘Previous family experiences of caring for someone with dementia and experiencing this as burdensome, also influenced perceptions of dementia and the significance of preventing it.’ |
| The plausibility and perceived effectiveness of dementia risk reduction | *‘It’s called senile, but it’s a natural process’*  *‘If you take care of yourself, you are going to be okay, and if you don’t, you won’t.’*  *‘I think if you’ve got it in your genes, there is nothin’ you can do.’*  *‘It has already affected my mother, but we may be all right if we change our lifestyle.’*  ‘The participants had resigned themselves to their inevitable fate that no one could prevent because of a genetic factor, despite anything they did: playing cards, performing brain activities and mental stimulation and taking medications to improve brain circulation.’  ‘The importance of lifestyle factors and healthy choices for promoting brain health were frequently discussed.’  *‘They say all of these things help, but I really don’t believe that.’*  *‘But at the same time everybody has got to grow old and you're bound to lose your faculties as you get older - you can't help it really.’*  *‘I don't think so [in response to question about whether would contact health professionals for memory concerns], not really, I don't think there is much they can do about it ... I don't have any faith in what they could do.’*  *‘I think [dementia] is a natural process of ageing for a lot of people and it isn't necessarily linked to a particular malfunction.’*  *‘I think I need to accept it as fate.’*  *‘We're old, right? In old age, there will be changes according to our age. Like we can't carry heavy things and our memory has lessened, see?’*  *‘For example, people say that the memory of older people is lessening, disappearing, and won't be developing like the teenage years.’*  *‘And so that's part of the, just, that's part of life. And, and you just go along with it’*  *‘What are we going to change? Do we eat better? But we already do that, or we should. Do we exercise more? We should do it. What are we going to change? ... You will not change anything; I think, honestly. It is one thing to say it.’*  *‘There's not a fix for this.’*  *‘No, there ain't no treatment; nothing you can do.’*  *‘I don’t believe that diet matters.’*  *‘I'm not sure that physical exercise is related that much to your brain.’*  *‘I only gave up trying to prevent it due to my family history.’*  *‘So I'd like to think that I wouldn't necessarily be so susceptible. But if it's genetic ... I don't know if there's too much prevention that you can do for it, other than having a healthy lifestyle, exercising, staying active.’*  *‘It might be too late for prevention.’*  *‘While I have to accept it if it comes to me ... I hope a good therapeutic drug will be developed.’*  *‘All of the above are recommended by various sources but I have no idea which actually work.’*  *‘I don't know whether it [diet] is going to help my brain or not.’*  *‘Everyone is a good candidate for brain health.’*  *‘I am not sure I will escape dependence, AD and other pathologies. But if I can do my best to stay active as long as possible, then I should do it.’*  *‘It gives me hope that I can compensate something.’*  *‘Prevention being the key.’*  *‘Being productive is so important to healthy aging, still being productive in later years.’*  *‘Because I think that I have a large chance [of developing AD]. My grandmother had it and my mother also ... What we are doing, it is as if I had already been told that I have Alzheimer's, so I am already in the field.’*  *‘I was very interested and enthusiastic and really quite excited about starting [the DRR intervention] because my mother suffered with dementia, so I am very aware of what the future might hold for me.’*  *‘So you could stay active and fight the disease as much as possible.’*  *‘You've got to keep on doing things ... Once you stop I'm quite sure that's when it gets much worse.’*  *‘Don't deprive the brain from any opportunities to make progress. Then it can recover slowly.’*  *‘[A friend] inherited a great memory from his mother. But he has also cultivated it, and that has been very important, by reading.’*  *‘New brain cells can still grow at this stage. New brain cells. As long as you keep stimulating them, they can still grow.’*  *‘The data of which I am aware indicates that other factors (diet, exercise, low stress, etc.,) are currently believed to be the primary drivers [of dementia], in addition to genetics.’*  *‘I am a little more careful because I have a family history. My mother has it so we may have it… I think it can be a problem for me to see how many of my mother's brothers are affected by dementia. Although the likelihood can't be 100% for us, I*  *believe that three out of seven sisters of mine, including me, could be affected by dementia just like my mother was.’*  *‘There’s a lot of things you can do to help yourself and there’s a lot of people that are [APOE allele] 4/4 who never come down with AD. I’m living in a manner that allows me to err in favour of health.’*  *‘It's certainly a case of use it or lose it. If you don't use it you're going to lose it’*  *‘Yeah, not having much [interest] into like, different things like books or ... I think that plays a big role into keeping your mind going, like books and crossword puzzles, anything newspaper, yeah.’*  *‘I thought it's probably something good. So why shouldn't I do it? At the time, I was some years younger, and it seemed like a good thing to do. There's no harm in trying.’*  *‘I think activity ... I'm certain of keeping doing the things you're able to do, love to do, clearly would help with all that.’*  *‘The socialising is clearly part of the picture it seems to me, yeah.’*  *‘I'd like to think that I have something to do with how it manifests. It's sort of like diabetes, which I have a very strong family history for. And knowing that there's certain things in terms of diet or exercise that research has shown may avoid triggering that genetic potential helps. You have the genetic potential, no question. Whether or not it shows up has a lot to do with what you do, your environment.’*  *‘And I hope, through physical and mental well-being, that we can find ways to protect ourselves and stay in good health as long as possible'.*  *‘[DRR is] intuitive.’*  *‘I believe that dementia can be caused by the complications resulting from diabetes. I saw patients who used to see a doctor for diabetes admitted to the nursing home for dementia. Therefore, I am concerned about what I can do if diabetes causes dementia.’*  ‘Some participants expressed doubt that dementia prevention is possible.’  ‘Participants frequently discussed an awareness of life course influences on brain health and the importance of taking care of oneself and how activity in younger years is important for aging well.’  ‘Residents often viewed AD as something potentially preventable.’  ‘Staying active: the value placed on this by participants and care partners in the context of growing older was striking. Included staying mentally active and staying physically active.’  ‘A few White men were skeptical about the relationship between PA and cognitive health. White women were less skeptical of the relationship between PA and cognitive health.’  ‘AD is considered like [a] disease one may contract regardless of what one does to prevent.’  ‘African Americans voiced concern that efforts to reduce risk might be ineffective.’  ‘All participants presented memory decline as expected and characteristic of dementia.’  ‘Participants who discussed being concerned about their memory felt there was little point contacting health professionals, such as their GP, as they perceived there was little that could be done, especially when dementia was perceived to be inevitable and there was no cure.’  ‘Some maintained beliefs that dementia is a normal part of ageing.’  ‘There were frequent statements that cognitive decline is to be expected with ageing.’  ‘Several participants expressed motivation to exercise to stave off the threat of physical and cognitive decline.’  ‘Across groups, participants frequently drew a connection between nature and brain health ... healthy aging is possible and by staying active in one's later years one could continue to keep their mind healthy and be productive.’  ‘Lifestyle changes were generally perceived as useful for the prevention of CVD.’  ‘Chinese Americans were the only group to voice hope for recovering cognitive function after its decline.’  ‘The importance placed on eating well and staying active, both physically and mentally, was apparent throughout interviews and highlights efforts to promote healthy lifestyles.’  ‘Other participants were motivated by the 'use it or lose it' idea, i.e., that it is necessary to keep the brain active in order to prevent cognitive decline.’  ‘Time spent with people is recognised as crucial for brain health.’ |
| The value contribution of other benefits | *‘It’s really important for us in the world to have a safe, healthy community and that’s what really helps to come to my exercise class’*  *‘I think [the program] is unique and interesting, as well as personally redeeming in terms of the health aspects.’*  *‘It made me feel like someone cared about me.’*  ‘For some participants, potential social benefits superseded improvements related to physical or cognitive health.’  *‘To improve my personal fitness.’*  *‘I lost 12 pounds and was pleased with a better body profile.’*  *‘I quickly experienced some rewarding physical improvements.’*  *‘During the sessions I actually felt better mentally and physically.’*  *‘I think just general health rather than worrying about staving off a particular disease.’*  *‘I wanted to get into a regular exercise routine and this seemed like a good opportunity to do so’*  *‘People ... want to be active, people ... want to exercise.’*  *‘I have other reasons, you know, like weight loss and heart healthiness ... I'm going to exercise for them.’*  *‘My attitude has changed towards my health problems and I see how a healthier diet can help my sleep.’*  *‘I'm two stone lighter and I feel much better.’*  *‘I've lost weight, and I've gained strength and mobility in my legs.’*  *‘Additional content is always good. I often use links.’*  *‘I’m just looking forward to the challenges, doing something new and different.’*  *‘When I first got involved, I didn’t understand the concept. We’re going to do what? ...You got the tablet out and you’d start looking at these maps. And you’d go, hey, this is interesting … it’s an interesting concept, in terms of the way it’s organized … you weren’t always walking along the same street in the same area.’*  *‘I’m looking forward to [using the application] partly because I really would like to know how [a tablet] works.’*  *‘I can't say that [difficult exercises] would frustrate me. I am just interested in solving them. If I don't succeed, I will try again for many hours, even at night.’*  *‘I'm a competitive person. I try to beat myself.’*  *‘[Reason for starting is to] pass time in the morning.’*  *‘Good transition to my retirement status at that time.’*  *‘Dancing is fun.’*  *‘I've always eaten this way therefore not seeing many changes in myself, however, the groups were enjoyable and informative [as reason for adherence].’*  *‘I would only consider the thing [brain-exercise games] as an entertainment thing, you know?’*  *‘I enjoyed the challenge. Even though I got frustrated with it [BFP], I did enjoy the challenge and it was interesting to find out what my failures are.’*  *‘Anybody can get out and walk. But why are you walking? And what do you have when you get home is something different ... more importantly, have something to document as a result of that walk.’*  *‘It was something different ... to blend the Afrocentric and historical fact-driven, and of a community that's almost lost. That is the pull. I mean, it really is.’*  *‘Normally, when you don't have any complaints, you don't visit the doctor, right? It feels unnecessary. So I'm glad we have this now, it makes me feel very safe.’*  ‘Motivators: regular check-ups offered a feeling of safety, control or being looked after.’  ‘Nonetheless, participants, including self-described “luddites” and “antitechnology” saw the pending transition to the application as an opportunity rather than a burden.’  ‘Participants frequently noted that the technology’s potential to integrate individual and community benefit was a principal driver to participation.’  ‘Exercises targeting concentration, memory and imagination skills; incorporating tasks and challenges related to daily life, and representing topics of interest were motivating.’  ‘Majority agreed that using an internet platform was a fun and/or effective way to improve health.’  ‘Adherence tied to an interest in contributing to our understanding of AD, the opportunity to join an exercise program, perceived exercise benefits and social support.’  ‘Challenge was the highest ranked motivational factor (25% of all recorded comments). Comments indicated that a 'good' challenge provides a means to experiencing a sense of achievement.’  ‘Participants spoke passionately about community benefits technology could facilitate … Potential community benefit carried greater motivational weight than potential individual benefit.’  ‘Getting a distraction during a stressful life period mentioned as reason for participation.’  ‘The program did not seem to cause fear among participants but offered reassurance that things remained well and they were being cared for.’  ‘Participants emphasised healthy daily health behaviours, including staying active and eating well, related to both general and cognitive health.’  ‘However, engagement with land-based pursuits is seen to have special benefits, including providing challenges and time to think things through.’  ‘17% of comments related to the games perceived practical benefits/potential for such.’  ‘9% of comments related to the importance that the games provide some form of basic entertainment.’  ‘7% of comments related to relaxation.’  ‘6% of comments related to a feel-good factor arising from a sense of accomplishment or achievement, either in terms of reaching milestones within the games or a sense that they had learned something.’  ‘7% of comments seemed to place a value on the potential of the games to pass time.’  ‘Health promotion: policy and practice initiatives. There was a general consensus that health promotion should not focus specifically on dementia, but prioritise a healthy lifestyle.’  ‘In addition to goal achievement, some participants reported other or unexpected benefits from taking part in the trial beyond those specified in the individual goals.’  ‘Expected benefits associated with participating in an exercise program a commonly described reason for adherence (27 out of 35 participants included in top 3 responses).’  ‘Participants suggested that social groups in the community or at seniors' centres could help mobilize people to maintain cognitive health. White and Chinese participants believed that promoting these groups’ activities as fun would motivate people to get involved.’  ‘The majority of participants explained that a dance class could provide an important opportunity for social interaction and that this would be an integral facilitator as well as a valued benefit.’  ‘For most participants, whether active or otherwise, physical activity was interpreted as a means to maintain health and therefore independence.’ |
| Weighing costs of dementia risk reduction | *‘If you give up smoking and booze and everything else and you're miserable, what's the point.’*  *‘A great percentage of us wake up every morning and just try to survive … To maintain brain health is far from our daily thought. Our truth is survival, not based on how important brain heath is to survive.’*  *‘I had such a lot going on in my life – my husband became very ill and I am now his full-time carer.’*  ‘Younger participants who are currently working and have children living with them stated that having responsibilities of having to take care of family members … were barriers.’  *‘I certainly would be quite keen to consider taking part in any trials, but if information came out about some of the side-effects, I might have second thoughts.’*  *‘A lot of it would depend on whether you could treat the side-effects. So if headache was a side-effect, a known side-effect of a drug that I was gonna be put on, or I was choosing to go onto, I'd say, well and is there any way of treating the symptoms. So if I've got something, that should the headache come on, I take something, and that solves that, I'd be reasonably okay. And I think, you know, tiredness, fine, you can go to bed, dizziness that might be more difficult to live with, unless you can find a cure for it. Stomach problems, how disruptive to your daily life will it be.’*  *‘If there's an 80% chance of the drug being effective and a 20% chance of getting dizziness, actually, you know, that sounds like reasonably worth throwing the dice for.’*  *‘If I didn't have high BP, I'm not sure it [antihypertensive medication for DRR] would be helpful. I wouldn't want my BP to be too low.’*  *‘I think I would probably have to withdraw my original statement about not hesitating [to take preventative drugs]. I wouldn’t hesitate to think about it, but obviously these risks and benefits would have to come into the decision process. So I probably would hesitate a little bit.’*  *‘All tablets are risk taking aren’t they? I mean the doctor can look in her little book and say, “This is what I am giving you.” They don’t suit everyone do they?’*  *‘The way I look at it is as a waste of time. To me it’s a matter of allotting your time. There are so many things to do.’*  *‘Well, I am not sure how much more I can reduce [my dementia risk] because of certain circumstances get in the way, for example, caring bits.’*  *‘When this job came – it threw everything out.’*  *‘What would motivate me to drop my bad habits ... I think being able to have opportunities to work part-time and what they call transition to retirement ... that would make me do more gentle exercises and probably encourage me to go and swim ... having the opportunities in time to do something little more gentle, yes, I would like to be able to ... if the workforce was more accepting of [transition].’*  *‘I'm afraid there's been too much in my life to devote to it that much.’*  *‘We were just too busy ... it was hopeless".*  *‘But [things] I really enjoy … like food … that would be much harder.’*  *‘It’s your personal choice if you are willing to sacrifice taste for health.’*  ‘*Yep, the doctor tells me if I quit smokin' I won't have it; I'd rather smoke and take a pill instead.’*  *‘If people have arthritic fingers that gets very tiring and wearing, particularly to try and do an hour at a time.’*  *‘So once old people heard they needed to pay, they quit considering to participate in those activities.’*  *‘Reduce the cost of healthy food.’*  *‘Some old folks think newspapers are too expensive. It's too much money for them.’*  ‘The side-effects that people would be prepared to tolerate varied, and revolved around the severity of various symptoms, whether or not symptoms could be alleviated and how much disruption they would cause to daily life.’  ‘Some participants reported cost as a barrier to healthy behaviours.’  ‘The financial cost of consuming a Mediterranean type diet was reported by participants as a factor influencing their dietary change.’  ‘Giving up enjoyment such as drinking was also reported as barriers.’  ‘Some did not initially hesitate to consider taking drugs to prevent dementia when asked early in the discussion about taking them. However, they sounded a more cautious note later in the discussion once they had considered the various trade-offs between benefits and harmful side-effects of drugs in more depth.  ‘Men identified taste preference, fast-food convenience and lack of self-control as barriers to healthy eating.’  ‘6% of comments related to a perception of the games as overly time consuming.’  ‘The impact of health issues on the ability to work on goals was noted. Changes in personal circumstances also negatively influenced goal attainment.’  ‘The demands of the farm were a priority over personal health needs.’ |
| ***Buying-In to DRR*** |  |
| Trusting sources | *‘I think brain science is evolving and so it is important to touch base with healthcare providers and also stay on top of credible research as available to public.’*  *‘The premise is that it [the information] is reliable ... I have learned that information and knowledge generated at the university is reliable.’*  *‘Discussion groups are realistic, which makes the idea realistic. People put it into practice right away. Those discussion groups are better than TV, newspapers, and magazines.’*  ‘They were prepared to follow the advice of their health professional, even if they did not always fully understand the rationale.’  ‘Internet in general was sometimes considered an unreliable source of information.’  *‘It was just very clear and it was via my GP. Then I think, well that is probably very serious. That is not … someone can’t do anything crazy with that or whatever. Reliable, that is what I mean.’*  *‘But they don’t listen to us. If doctor gave the form [information on diet and exercise], he would do it.’*  *‘I need to trust the doctor’s recommendations as a precautionary measure.’*  *‘Some doctors sometimes offer public seminars in hotels. I think those are very good. I have participated in one.’*  *‘I would like to know which physician to talk to about advances in preventing or reducing the risks of developing AD and dementia.’*  *‘In order to make progress, you must do what the doctor says.’*  *‘I accept it from her [healthcare professional] … if it is medically safe and they advise it, then I’ll take it. When the wise people say ‘it’s better for you’, well, they know better than me, so I just take the pills.’*  *‘You can only gain trust face-to-face.’*  *‘You're going to spread information, right? How do you let a person know that fish is good for the brain? A lot of people don't know. You have to introduce it to them so it's easy to digest. Automatically, they'll spread it among each other.’*  *‘I think promotions on television are the most convenient and beneficial.’*  *‘Seminars reach a limited audience and I would like to reach a broader audience.’*  *‘Sometimes a newspaper or television broadcasting company provides information about dementia prevention, including good foods or exercise. Then I have an interest in the information.’*  *‘They just need to put it [ways to prevent it] on the radio and on TV; people here listen to one or the other.’*    *‘I don't know if there's anything that they [media] know that is healthy for you.’*  *‘TV ain’t going to teach you nothing. It's not gonna help you in any way.’*    ‘The [study] platform was considered trustworthy as it was offered by the university.’  ‘As a confidence building measure, the platform should be run by an institution considered as trustworthy by older adults, such as the church, government agencies or medical service providers.’  ‘A personalised invitation letter, when signed by GP, conveyed a sense of reliability and trustworthiness.’  ‘A long-standing relationship was necessary to gain trust and, ultimately, to make changes regarding their health.’  ‘The use of oral tradition and learning through conversations and stories from other seniors.’  ‘Respondents described the importance of communicating with healthcare professionals, particularly PCPs, as well as family members, such as their partner/spouse, about ADRD … [but] shared their confusion about the most appropriate healthcare provider to disseminate information about keeping one's brain health.’  ‘All agreed that the educational material should be made personally available rather than through GPs.’  ‘Comments of encouragement were not considered sincere, due to the unfamiliar sound of the accent.’  ‘Little interest in accessing information via the web.’  ‘Regarding approaches to reduce the risk for cognitive decline, women said they obtained medical information from books, health fairs and the Alzheimer's Association. Men received medical information from senior centre, family and the Dept of Veterans Affairs. Women often took the lead role in providing healthcare information for their families. One man conceded that he relied on his wife to provide this information, whereas another said he consulted his daughter on healthcare topics.’  ‘Some participants believed that peer education, particularly within seniors’ social networks, was the best way to reach people with brain health messages.’  ‘Suggested that medical specialists be invited to conduct sessions at existing group meeting. Other than as guest speakers at group meetings, physicians were rarely mentioned as preferred sources for brain health information, and mainly by Chinese and Vietnamese participants.’  ‘Participants suggested TV as a communication source for promoting brain health due to its convenience and popularity, but also considered TV as a major barrier to engaging in healthy behaviours, radio was recommended as an important outlet for communicating brain health messages.’  ‘Newspaper and magazine articles were mentioned as important information sources in all groups. In addition, media outlets in native languages were mentioned as key information sources.’  ‘All participants discussed three channels: word of mouth, print message, and television.’  ‘Most proceeded on their own, carrying out intensive online searches for information.’  ‘Education both through the mass media and within social groups was also mentioned.’  ‘Church mentioned as an appropriate setting for peer education about brain health.’ ‘Some participants believed that peer education, particularly within seniors' social networks, was the best way to reach people with brain health messages.’  ‘Suggested targeting preexisting social groups, youth development programs through extension services, alumni associations, and seniors' centres, to teach and train established groups to discuss brain health.’  ‘Educating others could continue when the new learners pass the information on.’  ‘Participants also have shown the distrust in experts, which discourages them from following experts' recommendations to make behavioural and lifestyle changes.’  ‘Spokespersons more commonly recommended included doctors, celebrities whom people have faith in and active, healthy older adults.’  ‘Messages could come from credible sources such as the American Assoc of Retired Persons, the AMA, the Mayo Clinic, Surgeon General and Universities.’  ‘Participants felt that discussing concerns about memory problems would be easier with a GP with whom they had an established relationship.’ |
| Seeking certainty | *‘If you can clearly see [the cause and effects] you immediately change your behaviours. But you can’t.’*  *‘You have to prove to us that something works first. It's like [name omitted] was saying, we know about diets and stuff, but we don't know of anything ... I don't there there's anybody here that believes you can show us solid proof.’*  ‘The link between knowledge [of risk status] and action was seen as problematic given the unclear predictive value of risk information and the lack of proven options for risk reduction.’  *‘If I knew if I did something definitely that would, you know, prevent it, I’d do it probably, you know.’*  *‘If someone says to me if you don’t change your lifestyle you are going to be dead in five years [with dementia] I would say okay I would change it.’*  *‘You know I check and double check to make sure everything is written down, so if I*  *can just maintain where I am. That would be great too if I get better, you know … but as long as I know it’s not going to be worse than what I am getting now.’*  *‘There is no guarantee all the exercise in the world is going to do anything.’*  *‘If the probability was uncertain, I would also want to know it, but surely I would be less interested because I do not know if I can use this much to make decisions.’*  *‘The trouble is with these diets, again, you get it in the papers, ‘don’t drink tea … don’t drink coffee because it’s got that.’ And then a few months down the line, ‘oh tea’s good for you, coffee is good for you.’ What do you believe?’*  *‘I mean, you really can’t prove that if you’re like say – do more exercise, change your diet and all that, will it stop you getting that?’*  *‘Experts do not agree on what we should do to prevent the onset of dementia. That's why people like you are still doing the research ... I would like to be more aware of more definite things about dementia. It's all so vague.’*  *‘Well there's some [foods] that can be [good for brain health], but it's just like everything else. They're experimenting with them. They don't know for sure, so they say, this year, we'll eat fish. Next year, they may change their mind and say fish is bad for you. Like they do with cholesterol and everything else. They are not sure as they go through.’*  *‘With Alzheimer's, we haven't even figured it out yet. What causes it, how to prevent and treat it, and we are not clear about this.’*  *‘If this medication [antihypertensive] was shown to prevent the onset of dementia wouldn't there be sufficient evidence that it would reduce my risk.’*  *‘It would be nice to know if exercise actually will help prevent this memory loss.’*    *‘I was hoping that if I got this [genetic risk] information I would have this great transformation … it didn’t happen like that … it was nebulous … if it had been real concrete – you know, then I’d get things planned … but nope.’*  *'You know, 'cause a lot of people come down with it and they, you know, be in good shape and do what they supposed to do and eat what they supposed to eat. But still, they come down with Alzheimer's.’*  *‘I find it interesting that some people with a wonderful brain and who have done amazing work and they still develop dementia. They tell you if you keep using your brain, you are less likely to get dementia ... they are obviously still using their brain and they get it.’*  *‘I've seen people who are, who are real active but get Alzheimer's. Real active.’*  *‘Well, they're always suggesting that you do this or you do that to keep you and you work crossword puzzles and do this and you work, do these things to keep you mind sharp and you belong to all these different things to keep your mind sharp and you exercise. But, that doesn't necessarily mean that it's gonna help because the people, some people you know and have seen, why they still get it.’*  *‘My mother did that though. She watched her diet and she was very careful and doggone it, it didn't keep it from happening.'*    ‘Both women and men said there was a need for proof that preventive strategies were effective for promoting cognitive health before they were willing to make changes.’  ‘Willingness [to learn genetic information] shaped by perceived certainty of the information.’  ‘Respondents perceived that evidence was either insufficient or not convincing to warrant taking a daily medication to prevent dementia.’  ‘Willingness to learn biomarker results shaped by perceived certainty of the information and the ability of participants to act on it.’  ‘There was a tendency for many participants to see proactive approaches as providing more definitive information than is the case. The uncertainties around proactive approaches were challenging for many participants and the importance of help with interpreting information about risk and how to act on it were stressed.’  ‘Main reactions to being told 'scientific research suggests being physically active may help people keep their brains healthy and prevent or delay AD and dementia': … there is no guarantee that PA will prevent cognitive decline.’  ‘A theme identified in responses of African Americans, American Indians, and Whites, was confusion caused by conflicting and changing media messages about both general and brain health.’  ‘The link between knowledge and action was seen as problematic given the unclear predictive value of risk information and the lack of proven options for risk reduction.’  ‘Participants also pointed to research findings that do not provide clear relationships between causes and dementia as another barrier.’  ‘Concerns were expressed about frequently changing advice on lifestyle since this created uncertainty over how to act on such information and potentially undermined the potential value of both health promotion and providing risk information.’ |
| Avoidance | *‘There’s a lot of pensioners that don’t want to admit they’re getting old and they don’t like being lumped in with old people.’*  *‘Fear could stifle all the levels of growth.’*  *‘If you’ve got memory problems, you don’t want to be reminded of it all the time.’*  *‘Why scare them … you’re trying to get them to move.’*  *‘I don’t think I’d want to know [risk of dementia] because I think it would affect how I encompass my life. I’d rather carry on in blissful ignorance.’*  ‘Participants felt that proactive approaches while asymptomatic could create considerable anxiety. Perceptions of dementia were thought to influence the psychological consequences.’  *‘Don’t want to deal with fear.’*  *‘I don’t want to know that I might be going to get it. I don’t mind being told that ‘if you do this, this, and this you’re less likely to have it’ just as with heart disease or anything like that.’*  *‘Having taught in a further education college and taught health and social care subjects, 16 to 19 year-olds think they're going to live forever. When you try to talk to them about smoking and drinking, it's just over the top of their head.’*  *‘We haven’t been to our so-called Autumn Center, ever, because that’s for old people. . . . We’ve never been there. Because we’ve always thought, “Oh, that’s for old people.” So we’ve never gone.’*  *‘Prefer messages that take a positive approach.’*  ‘There were mixed comments about including a fear component. Across all race and gender groups, most responded that they 'don't want to deal with fear' and would prefer messages that take a 'positive approach’.’  Some White women said fear should be incorporated into the message; mentioning nursing homes and loss of independence; describing life with AD; and mentioning potential warning signs of dementia such as forgetfulness.  ‘Some suggested it was wrong to exclude factual information, even if it meant including information that may scare some who receive the message.’  ‘In the context of no cure for dementia, knowing your risk of developing dementia was seen as unhelpful by some healthy volunteers.’ |
| ***Turning Beliefs into Action*** |  |
| The impact of intervention characteristics | *‘It's like a prescription for each person depending on their abilities, physical and mental.’*  *‘I said ‘yes’ because, with internet, it is not constraining. I can access the website anytime, even at night.’*  ‘Across all groups, several participants mentioned it would be helpful to include multiple activities in communications to provide choice.’  ‘Adaptive level of task difficulty seemed an important prerequisite for all participants in order to ensure long-term commitment to the regime.’  ‘Good accessibility facilitated participation.’  ‘All participants reported they would like to receive feedback and appreciated opportunity for practice as often as they wished. Performance feedback should not be presented during the exercises.’  *‘It's an individual choice - it might suit some people but not others.’*  *‘[The amount] depends on the individual.’*  *‘Everyone has his own unique way ... activities will differ from person to person ... depending on the physical condition.’*  *‘Have a group leader. We were lucky that we had a leader that stepped up because it just kept things tied together. And all I knew was, on each of those three days I was going to get a text to meet on this corner at this time. And that worked so well.’*  *‘When [the application] worked, [it] was great.’*  *‘I was distracted by the tablet. So, I wasn't fully engaged with the conversation that was taking place. And I wasn't engaged with ... the visual surroundings ... I was more focused on the tablet and where I was walking, to make sure I wasn't going to fall flat on my face.’*  *‘I've got mine [computer] set up so that letters are a lot larger, so it's easier to read. Like even if I want to do a book or a magazine or paper or anything.’*  *‘Keep it as simple as possible for the seniors.’*  *‘Pictures for senior citizens are real important.’*  *‘I read pictures more than I do the words.’*  *‘I quite like having a bit of a challenge. Something to aim for that’s quite important.’*  *‘I enjoyed the math ones [games] better than matching shapes [games] … I'm an accountant and numbers are my life.’*  *‘Is there a time limit? Do you have to complete the task in a certain amount of time?’*  *‘I read pictures more than I do words.’*  *‘I am an aesthetic person, not a matchstick man or something like that.’*  *‘I like something fairly concise.’*  *‘The internet would be a good back up, but not everyone with memory problems would look at the internet.’*  *‘Leaflet, you have more time to study it, I think so.’*  *‘Wall charts, that you could place things on that you could say you've done it, step-by-step process, something that is interactive. That would be important.’*  *‘We have limited space for a wall chart so maybe a leaflet’*  *‘I think it would make it more individual, more personal if it was posted out.’*  *‘Would have preferred food recommendations to include traditional foods.’*  *‘[Persons beginning a PA program should] start very low and then build up.’*  *‘If you could make some kind of table or some kind of listing of alternative things that are equivalent. A person rides a bike or walks a mile vs a person lifting weights for 20min or jump rope ... that would be a tremendous help to see this listed.’*  *‘[Print messages should have] big, bold writing.’*  *‘[Messages should be] short, sweet and to the point.’*  *‘Things popping up, but things more widespread across the community so that people will come across them more frequently. In what shape or form, I suppose that’s debatable.’*  *‘The activities could have been broken up a little differently. You knew every time that you went to the computer you would be doing ‘high and low’.‘*  *‘The program treats you like an idiot and keeps repeating the instructions that have been repeated on every previous day.’*  *‘Oh, I just didn't like how they said some of sounds. It wasn't until I saw the sound written down as letters that I realised what the sound was.’*  *‘If you listened to all the narratives and also to the little cartoony things it would take that much longer again and I must say I only listened to about four or five of the cartoons but I just didn't think they were appropriate to our culture.’*  *‘A training schedule would be good, something to train me to keep my memory.’*  *‘Several packs sent out over a period of time would be good.’*  *‘But if we had to fool with this device…’*  *‘The [application] worked. But when it didn’t … we were lucky to have the book to fall back on.’*  *‘Keep in mind you’re dealing with people who are not technology savvy because technology came in after us.’*  *‘The pack was a good reminder to improve my diet.’*  *‘I find them [brain training exercises] quite challenging. When I finish I think: 'see if I can better that score'.‘*  *‘I think having that pressure of time, can increase anxiety.’*  *‘Give specific examples of gentle exercises for the less physically-abled such as sitting exercises or exercises that could be done in the home.’*  *‘List out types of exercises for the less physically abled.’*    *‘It was taking place not far from where I was working.’*  *‘I liked the facilities.’*  *‘I just wondered how many visits there would be, and if I have time to attend all of them, but ... There weren't a lot of visits. I didn't really think about it very much ... It was an automatic decision.’*  *‘If the information is on a CD or DVD, then they [patients] can watch it at home. If you broadcast it, they can only listen to it. But, if you put it on a DVD, then they can watch it on their own time.’*  *‘Pick out places that they could go and exercise, and just, you know, eat.’*  *‘The park management should organise activities like Tai Chi or aerobic classes... this can attract more people to go out to the park to exercise.’*  *‘Organise more activities and invite others to join free. It could be a field trip or physical exercise.’*  *‘[Walking is] pretty much free [and] simple ... anybody could [walk].’*  *‘[The] portability would be motivating. The fact that I could sit down at a chair that I thought was comfortable and do it.’*  *‘[Feedback is important] to see where I stand, what I am able to do, what is required from me. I believe it is very important to have access to this information.’*  *‘I think I would have felt pressurized if I’d had phone calls.’*  *‘It [mentoring] gives you an incentive being spurred on … I wouldn’t have stuck to the diet and walking without it.’*  *‘It [the goal setting process] was quite easy. It was obvious what I needed to do.’*  *‘It's easy to set them[goals]. It's hard to keep them.’*  *‘[Goal setting was] Pretty easy, I could come up with quite a few.’*  *‘I'm too independent, really. I'm quite capable of setting goals for myself, you see.’*  *‘Having the goals probably was something to aim for, that somebody was going to ask me about in a year's time.’*  *‘Need constant reminders.’*  *‘Remind them every day. Each day, remind them.’*  *‘Just because I didn't do them [goals] doesn't mean I haven't done other things. Except, as I say, maybe choose different things to do to suit me better.’*    *‘I would much rather work on positive changes to my lifestyle and nutrition than to take medication [antihypertensive for DRR] for anything.’*  *‘I am not a fan of medications as an initial choice of treatment.’*  *‘I would want my medical team to take a conservative approach, as I believe medication is over-prescribed in the US generally.’*  *‘On this page we had to list everything that you ate that day, how much. And what we were watching was the sodium. And when I did that, I realised that my portions were too large.’*  ‘32% of comments related to boredom within the games.’  ‘A small number said that they were motivated to set their own goals and did not need a formal goal-setting process.’  ‘Suggested approaches for motivating people to engage in preventive behaviours and activities to reduce the risk of cognitive decline. Participants suggested both one-on-one and group approaches.’  ‘Several participants preferred a conservative approach to medicine, favoring prevention alternatives such as lifestyle and behaviour changes. Specifically, they emphasised their preference to maintain healthy nutrition, reduce sugar intake, bolster fruit and vegetable consumption before resorting to pharmaceuticals.’  ‘Numerous barriers described that impeded older people from accessing the natural and cultural spaces and places that they closely link to identity and health. For example, structural pressures are restricting access to the land and natural resources through mechanisms such as government regulations around hunting and fishing rights.’  ‘Mainstream healthcare was unnatural and insensitive and thus to be avoided.’  ‘The pharmacologic and institutional treatments prescribed by the mainstream health care system conflicted with the older women’s values and beliefs for natural sources.’  ‘Design, time allocation and repetitiveness of the exercises were suggested by some participants as troublesome and decreased their interest in the program.’  ‘Only Chinese participants suggested organising activities to help promote brain health.’  ‘Participants endorsed the BFP for the following features: program flexibility, as it can be used at any time that suits the individual; training can be done at home so the requirement for travel is eliminated.’  ‘Most people found the goal-setting process straightforward.’  ‘The process of goal-setting enhanced motivation and the commitment to work on the goals encouraged individual effort.’  ‘The mentoring was positively received and motivated people to work on the goals.’  ‘Majority felt something to refer back to would be helpful, like a booklet or leaflet. Some, though not all, liked the idea of a wall chart.’  ‘14% of comments related to the need for feedback regarding progress.’  ‘7% of comments related to an appreciation of positive affirmation from the games.’  ‘Additional factors recognised by several participants as being important to increase PA levels included self-motivation and having a schedule or 'routine' for PA.’  ‘The convenience of having continuous access to an internet intervention were mentioned by some. Interviewees with more computer experience, in particular, thought that internet could be a useful tool to improve lifestyle.’  ‘Small number of visits, occupying free time, getting a distraction during a stressful life period and getting new information about CVD and memory disorders were mentioned as reasons for participation.’  ‘Participants preferred designating a leader already familiar with smartphone technology and/or who showed interest in learning and handling it.’  ‘For the most technology-reticent participants, flexibility allowed participants to perceive technology as useful, develop a positive attitude about it, and derive potential benefit from it without developing an intent to use it themselves.  ‘Participants felt introducing technology to older adults should be gradual, with in-person assistance in initial phases and an uncomplicated interface free of unnecessary layers.’  ‘The first two triads appreciated starting with the familiar (paper-copy routes) and transitioning to the unfamiliar (smartphone application) once revisions were made because it allowed practice with a fail-safe replica, keeping the priority on developing the health behavior rather than on the technology.’  ‘Not surprisingly, participants emphasized that paper copy routes were imperative.’  ‘Frustrations were largely due to malfunction, not to application content or concept, and belief in the application’s potential to sustain motivation to walk remained high.’  ‘Technology failure contributed to distracted walking and undermined confidence.’  ‘Challenges identified included need for flexible timing for people who are working.’  ‘Regarding message content, participants suggested using slogans and testimonials to inform the public about maintaining brain health.’  ‘All participants expressed a strong interest in using an internet-based platform for cognitive training.’  ‘All six participants with MCI claimed they would be willing to use such a platform on a regular basis.’  ‘Prefer regular compared to sporadic training sessions.’  ‘Training adherence should be strictly voluntary.’  ‘Participants preferred challenging exercises.’  ‘Should be rich in variety and implemented in a playful way.’  ‘Design of user-interface was important - preference by some for 'state-of-the-art.’ ‘Noise/bright colours/animations were potential distractions.’  ‘For obvious reasons, the access to communication features should be both straightforward and senior-friendly.’  ‘All seniors with cognitive impairment emphasised the benefits of audio-video communication. In contrast, the participants without MCI reported reluctance towards using AV-communication for making initial contact with strangers. These participants favored a more anonymous approach via personal messages of emails.’  ‘Other seniors reported they were not willing to disclose any information about themselves and could not see any additional benefit in revealing their date of birth or address, etc.’  ‘Some participants indicated that the extent to which the sessions changed their behaviour was limited by the degree of accord between lifestyle changes suggested and their current practices.’  ‘A participant with MCI spoke of an intention to use the manuals as a resource when planning diet.’  ‘MCI patients preferred educational material to be concise, eye-catching and in written format, with personal delivery of information, e.g., dos and don'ts list, recipes, meal plans.’  ‘Include pictures/cartoons within the text and plenty of colour. Few had computer skills or access to a computer.’  ‘13% of comments related to the importance of familiarity in terms of the content of games.’  ‘Usability issues was the highest ranked de-motivational factor (27.6% of comments). Often related to physical problems interacting with the technology due to the device or as a result of ageing.’  ‘Participants across all groups often recommended messages emphasising walking because of cost and simplicity.’  ‘Participants also recommended listing activities that persons with physical limitations could do, such as chair exercises.’  ‘12% of comments related to timed games being too fast.’  ‘7% of comments related to a perception of games as being overly difficult.’  ‘The most common suggestion for introducing proactive approaches was to embed them in routine health check-ups, for example, the [UK] annual health review of older adults. Routine risk assessments for younger adults, especially those at high risk, were also suggested. This was seen as preferable to introducing a new approach focusing exclusively on dementia and thought by participants to 'normalise' the inclusion of potentially anxiety-provoking questions. A few participants suggested that alternative venues such as community centres or health buses would offer more relaxed environments.’  ‘Reasons for interest waning over time included repetitive nature of exercises, the large time commitment the program required and high levels of frustration due to the American presentation style.’  ‘Meaningfulness of specific BFP exercises affected motivation levels of participants.’  ‘Participants preferred to be made aware of training weaknesses to focus sessions on improving these.’  ‘Summary of individual results after completing an exercise clearly preferred.’  ‘Participants with MCI expressed a considerably stronger preference for direct personal feedback.’  ‘Participants were open to learning new technology, not only because it was relevant to carry out their 'jobs' in the program, but to mitigate other modifiable risk factors for cognitive decline. Trying new technology was framed as one of many changes to which seniors must adapt.’  ‘Participants identified barriers to healthy eating. Women said they opted for quick and convenient meals when cooking only for themselves.’  ‘The nonpharmacologic nature of the intervention attracted some participants.’  ‘Half of the participants stated that it must be guaranteed that all personal data revealed on the platform is secure and will be handled confidentially.’ |
| The importance of personal will | *‘You cannot motivate others, they’ve got to want to do it themselves.’*  *‘I think that I’m pretty strong physically and a mentally active person and I’m going to keep on that path because I’m very motivated and driven.’*  ‘A sense of personal responsibility and self-initiative came out through discussions.’  ‘Some participants expressed concerns about being able to motivate others, saying nothing works for influencing people to take care of their health.’  ‘A number of interviewees expressed the importance of their autonomy being respected. Some were not prepared to follow any advice on specific domains at all (PA, eating breakfast, smoking, drinking alcohol). Continued efforts to change these behaviours by nurses who were unaware of the underlying views or convictions caused resistance.’  *‘I think I'm fairly well self-motivated, but I fine-tuned some of them’*  *‘If I want to do it, I will do it on my own.’*  *‘You have to be a self-motivator – whatever that might be.’*  *‘No, I don't think it would have made a difference because I am determined. If I want to do it, I will do it on my own.’*  *‘Sometimes it's motivation or whatever and it's stickin' to it. And it's easier not to bother.’*  *‘I have a couple of sisters … I mean, there are too many things out there to do. Get outside and walk. They won’t – you just have to ask them, that’s all – that’s the only thing you can do.’*  *‘’You have to’… always ‘you have to.’ I detest it.’*  *‘A lot of them say I don’t want to go. I’m not gonna change my eating habits.’*  *‘You can take the horse to water, but you cannot make it drink.’*  *‘Because there are a lot of people out here that just have no interest in all these things until something happens to them. It's really a hard thing to do.’*    *‘To live healthily, you do it or you don't for yourself, that's up to you.’*  *‘People should not be forced into doing things in Germany.’*  *‘It’s an individual choice.’*  *‘I don’t think we necessarily have to enforce people to make, to go a certain route, but to have the information that you’ve got a predisposition towards something, well OK, then I may choose – or I may not – but I may choose to change my diet, to take more exercise, things that will actually potentially help the situation, may not, but that’s my choice.’*  *‘So to speak I would ‘knock on the door’ first with a message: I found you and I read that you are interested in this and that. Would you care to swap ideas about it? Because I am a polite person, I would never start a conversation immediately over the telephone.’*  ‘Participants reported negative attitude and stubbornness as barriers to motivating others.’  ‘Suggestions as to what would be helpful in order to change diet were related to the background desire to change diet.’  ‘All participants agreed that if an individual wasn't interested in improving their own cognitive health they would not persevere with the BFP.’  ‘Others were concerned about the potential loss of individual freedom.’  ‘For lifestyle issues, however, participants all felt this was, and should be, entirely their own decision.’  ‘Suggestions as to what would be helpful in order to change diet were … related to the background desire to change diet.’  ‘Participants who succeeded in making changes reported that it had been primarily their own decision to change behaviour.’  ‘They mainly asked questions and discussed issues in an open conversation, but stayed away from giving directions or general advice. In this way, participants felt heard and respected, and were more likely to stay engaged in the consultations.’ |
| Reciprocity between self-efficacy and behaviour. | *‘Nothing would make me increase my PA. No- too many health problems.’*  *‘[Participation was] difficult, because it involved stepping out of my comfort zone.’*  *‘I think I will have the strength now to do it without anybody prodding me.’*  ‘[Participation facilitated] increase in perceived control over health status.’  *‘Depends on current diet – difficult for elderly to change.’*  *‘Not feeling comfortable or confident with this piece of machinery in my hand ... so my curiosity was 'is it working? Is it working? Is it doing what it's supposed to do?’.. that level of anxiety was always there.’*  *‘It wouldn’t encourage me to increase my activity as I am not physically capable – too many health problems.’*  *‘Felt uncertain about what to do.’*  *‘I can’t do anything because of this arthritis so I’m no good doing these goals.’*  *‘It wouldn't encourage me to increase my activity as I am not physically capable’*  *‘I'm not capable of much PA except walking.’*  *‘Not physically capable.’*  *‘I used to play snake on the mobile but as you get older it gets harder, firstly, from an eyesight point of view and secondly because they become fiddly.’*  *‘You can see the challenges with that [having a disability] in a place like this, and even organised activity, when you can no longer go on your own to the, to the fishing and the hunting, you know, or just outdoors.’*  *‘All my computer skills are very limited but that was no problem at all.’*  *‘I feel like I’m taking charge of myself.’*  *‘I'm able to get this thin [iPod touch] now. I couldn't get it on in the beginning. I have learned that today. If you gave me it yesterday, I couldn't do that.’*  *‘This program has started the wheels turning with respect to ideas for lifestyle additions that I can see myself making gradually.’*  *‘A feeling of better self-esteem because the things I’d let slip I picked up again and succeeded.’*  *‘[The program helped] "My attitude to what is still possible despite my medical condition.’*  *‘I did find myself having – getting some confidence back.’*  *‘After 15 minutes couldn't concentrate.’*  *‘Totally de-motivating because of [the] result.’*  *‘It kept my interest more at the beginning, more so than at the end because I think I was feeling I wasn't improving very much.’*  *‘No one should get discouraged.’*  ‘Goal attainment was associated with a sense of accomplishment.’  ‘Narratives at first and second follow-up suggested that the groups had increased their understanding of diet as an active choice.’  ‘The process was empowering and people reported taking more charge of their lifestyle.’  ‘Facilitators of change appeared to be … increased self-efficacy.’  ‘Some participants and care partners described maintaining control over MCI, while others reported feeling that their life course was out of their control.’  ‘Lack of confidence in computer skills was a barrier.’  ‘[Perceived program benefits]: increased understanding of diet as an active choice.’ |
| ***The impact of social factors*** |  |
| Social expectations | *‘I try to be very dependable. If I say I am going to do something, I will do my best to see it through.’*  *‘I'm excited to see everybody and hear what they have to say and share maybe a little bit of me too.’*  *‘Dancing for the seniors isn't dancing to get sort of super fit or anything like that. It's a social thing.’*  *‘Social pressure can cause you to change your lifestyle like in your working environment. It became unacceptable to smoke in your office.’*  ‘Participants also discussed the importance of exercise buddies who can keep them ‘accountable and motivated’.’  *‘I enjoyed the workouts and socialising with the other members.’*  *‘To spend some time with my sister [who is also in the program].’*  *‘We [the group] had a competitive edge was the constant draw. Yes, competition.’*  *‘If it was brought into a social thing [situation] where a family could play ... plug it in ... from the iPod to the TV where you have a larger screen.’*  *‘You give little bags where they can put their dancing shoes in, with the logo on it. Then they'll remember to bring their dance shoes with the logo on, like the dance club, you know, and, and that gives them a little bit of a feeling of belonging then, rather than just walking up with a pair of old shoes on ... You started something that they actually belong to.’*  *‘Nice meeting new people who undergo similar things. Some of us are still in contact.’*  *‘[Social interaction is a] good way to lighten up your own burden ... [and it provides opportunities to] learn new things, engage with active people and stay sharp.’*  *‘Bringing the seniors together socially is good, that's a good thing, because they're able to interact with each other and reminisce and I find one of the things that we do, there, monthly is the last Saturday of each month we have a music and friends' night and it's a time to reminisce and sing some of the old stuff that they used to and they seem to really enjoy that, right? They talk the old days as well, so it's a new venture for the community. The [study centre] only opened a year or so ago.’*  *‘Well, I guess it was dutifulness.’*  *‘And I think it would be an insult to those who have treated me if I didn’t do that. A piece of gratitude also. And I think that is a sort of obligation that I feel.’*  *‘There are ways to avoid becoming boke [senile] or to fight it and if you do not, that is embarrassing because you were too lazy to have avoided it.’*  *‘Having determined I was going to do something and somebody else knew about it … my self-esteem wouldn’t let me not do it. It does make you stick at something.’*  *‘It reminded me that someone was checking on me so I had to keep it up’*  *‘I think it's good that you keep on top of us otherwise you'd go back to the bad ways.’*  *‘It [mentoring] gives you an incentive being spurred on … I wouldn’t have stuck to the diet and walking without it.’*  *‘My wife made me do it.’*  *‘Not everybody is going to get that education, not everybody is going to listen to that education. And there's parental influence on it, social influence on it, economic influence on it. So I think you've got sort of - I believe very strongly in personal choice, but I think there's a point where you've got to look at it more on a sort of society level.’*  *‘With healthy eating, at least now they're going to put this sugar tax on, but I would put tax on a load of other things like that.’*  ‘Some Dutch interviewees felt it was an obligation to themselves and/or their physicians to pursue a healthier lifestyle.’  ‘The importance of social interaction and communication, even in an internet-based trial, was emphasised.’  ‘Facilitators of change appeared to be … positive social experiences in the group.’  ‘Participants suggested that social groups in the community or at seniors' centres could help mobilize people to maintain cognitive health. White and Chinese participants believed that promoting these group activities as fun would motivate people to get involved.’  ‘Taking certain foods or enforcing limits on the food industry were most frequently suggested as ways of enforcing a healthier lifestyle. The former was welcomed by some.’  ‘[Participants] discussed health promotion and social integration with respect to brain health and memory as important.’  ‘Group outings were thought to facilitate a healthier diet, physical activity, entertainment, and mental stimulation.’  ‘Positive social effects of group [were an enabler].’  ‘Some participants reported enjoying or benefiting from the opportunity to meet together to share ideas, and the social aspect of the groups. This continued even after the 2-month follow-up, in which some participants decided to meet informally.’  ‘7% of comments related to a perception of the games as socially isolating.’  ‘11% of comments related to the need for the games to have some element of social interaction.’  ‘Participants also mentioned they would like more group exercise classes where there is opportunity for social interaction, such as walking clubs, water aerobics courses and dance classes.’  ‘Dance seen as a means of promoting social interaction.’  ‘The majority of participants explained that a dance class could provide an important opportunity for social interaction and that this would be an integral facilitator as well as a valued benefit.’  ‘Other motivators for behavioural and lifestyle changes were identified from having worked in the past, including social pressure.’  ‘A small number of participants suggested that efforts could be directed toward formally developing a group identity for the proposed session. There may be tensions regarding the type of group identity constructed.’  ‘Views [were] different over whether health promotion campaigns should target children and the role for schools in promoting a healthy lifestyle. Support at a government level was clearly relevant. Concerns over the lack of reach of these types of initiatives led some participants to suggest that policy changes might be more effective. Taking certain foods or enforcing limits on the food industry were most frequently suggested as ways of enforcing a healthier lifestyle. The former was welcomed by some. Others were concerned about the potential loss of individual freedom ***[Ed note: use this in describing the contrast between this theme and that of the importance of personal will]***.  ‘Two participants missed the real-world human interaction.’  ‘The possibility of increasing stigma towards people with dementia, was particularly evident during some task groups in which participants spoke pejoratively about individuals who were perceived not to respond 'appropriately' to risk information.’ |
| Delivery of interventions | *‘She never tells us what to do, not at all. She likes it when I tell her, that’s why she’s so good. We live healthier now.’*  *‘I had hoped for more personal questions, more attention and personal contact. They should be much more people-oriented if they want to keep people involved.’*  *‘Lifestyle advice, I think that's meddlesome. But that's unkind to say".*  ‘The aversion to lifestyle advice seemed to be caused by its directive and moral nature. Participants felt judged, rather than encouraged to change their behaviour.’    *‘[The nurses] did their job very well [...] Not hurried but still goal oriented and you can also talk about other things and get them off your mind. I can ask them anything. And I also get a decent answer. It's excellent.’*  *‘Well, the person [delivering the intervention] should be doing the same thing as me on the platform and we should share the same interests.’*  *‘So to speak I would ‘knock on the door’ first with a message: I found you and I read that you are interested in this and that. Would you care to swap ideas about it?’ ‘Because I am a polite person, I would never start a conversation immediately over the telephone.’*  *‘In this way [by frequent visits to the same nurse] you get an open relationship. For example, I can tell her everything. With others, you keep more distance. But with her, I know exactly where I stand as she does with me. She tries to do the right thing for me, that's how I see it.’*  *‘We did talk about certain things, but it was not advice but, um, more like a conversation. What you can do to maintain your weight, those of things.’*  *‘We did it [goal setting] together really, didn’t we, so, yes, it was fine.’*    *‘They don’t ask you what you want to do about it yourself. I think it’s a bit offensive for elderly people. They should use another method actually, I think … Ask more questions about your constitution, how you are doing and what you can do as an older person in your home etc.’*  ‘Barriers were lack of relationship or coaching approach by nurse and frequent changes in nursing staff. Impersonal, one-way communication and lack of detailed questioning.’  ‘All participants stated that training sessions should not take place in a classroom atmosphere.’  ‘Focus groups most often suggested interpersonal communication as a strategy for motivating others to keep their brains healthy.’  ‘General preventive advice was considered unnecessary or patronizing, but practical support was appreciated.’  ‘Personal relationship with nurse and nurses using a coaching approach were facilitators; the practice nurse was viewed as a dedicated person with up to date medical knowledge and a sincere interest in their personal circumstances.’  ‘The term lifestyle advice was generally regarded as patronising and included things that were already known or that were not relevant to the participant.’  ‘Positive effects of coaching attitude: asking questions/listening allowed gaining trust; recognising and reinforcing efforts that had already been made; able to discuss tangible, tailor made plans with participants.’  ‘Successful nurses operated in a sharply defined area: they mainly asked questions and discussed issues in an open conversation, but stayed away from giving directions or general advice. In this way, participants felt heard and respected, and were more likely to stay engaged in the consultations.’  ‘The interviewer's facilitation of the goal-setting process was important in helping to identify individual goals. Guidance and prompts by the interviewer aided this process.’  ‘Suggestions as to what would be helpful in order to change diet were [either related to the background desire to change diet or] related to the delivery of the EM.’  ‘15% of comments related to poor communication, usually poor instruction.’  ‘Participants highlighted the difficulties in getting appointments with GPs and the limited time available within appointments.’ |
| The importance of peer support and examples | *‘The group was an excellent source of encouragement.’*  *‘We can do it, and they can follow.’*  *‘They see clearly, 'Ahh ... this man is 90 years old and is still like that, how is that possible'?*  ‘A form of motivation discussed was use of social comparison. Primarily white and African American participants recommended that other older adults could be motivated to engage in healthy behaviours by comparing them to others of their same age.’  *‘Bring the friend. You encourage each other.’*  *‘I do a lot of talking and my friends ... they just stay home in the house. You should try to encourage them to come out, get motivated. If you don't do nothing but just come out three times a week, that will be plenty.’*  *‘By going to ask someone, they'll get used to dancing and interacting with everybody. And slowly, if people are turning up every week, or every other week, they will get used to chatting and talking and will want to go again.’*  *‘I’m looking forward to [using the application] partly because I really would like to know how [a tablet] works. And I would feel more comfortable in a group of people that were doing this thing than just trying to figure it all out and do it all by myself.’*  *‘I think if you watch the Olympics and this other guy's skiing with one leg ... that would motivate anybody if he can do that.’*  *‘I think people can follow your lead and be exposed to the opportunities that exist ... I think the worst thing is to sit at home, shut in ... take them with you if you go dancing ... take them for singing. If they like it, they join.’*  ‘Others discussed the importance of witnessing individuals with physical impairments becoming physically active.’  ‘Participants also suggested motivating others by encouraging them to engage in healthy behaviours and to get out and socialise.’  ‘Workplace and family support were identified as motivators to change health behaviours and lifestyle.’  ‘Participants stressed the importance of providing appropriate support services, particularly for people with no close family and limited social networks.’  ‘Motivating others by example was suggested by all groups, except for American Indians.’  ‘Adherence tied to … social support.’  ‘Learning new technology as a group curbed hesitation and bolstered confidence to engage curiosity.’  ‘Chinese and Vietnamese participants mentioned ways to encourage healthy behaviours at support groups. In particular, support groups for people living with various health conditions could be helpful for motivating participants to engage in activities that promote brain health.’  ‘Comparison with others only viewed as useful if there was a possibility to compete.’  ‘Another method, recommended by African Americans, was pairing two older adults together who could motivate each other to engage in healthy behaviours.’  ‘Participants in all groups often reported needing support from others, such as receiving encouraging words from a friend, obtaining professional exercise instruction or playing with grandchildren. White men discussed the importance of encouragement from their wives.’  ‘They felt that verbal encouragement, reassurance and feedback from a human would have resulted in a more positive influence on them and assisted them to keep motivated.’  ‘Using older adults' testimonials about health as a communication strategy.’  ‘Three quarters of the interview partners also stated that additional communication options would be very helpful and useful. According to them, communication could service as an ideal way of increasing motivation and providing support.’ |
| ***Personal Experience*** | *‘I know too many people that did literally everything 'right' and still ended up with dementia.’*  ‘After looking at dementia’s progression in family members, they realised the importance and necessity of prevention and saw an opportunity to change their life by correcting their lifestyle habits.’  *‘I believe that dementia can be caused by the complications resulting from diabetes. I saw patients who used to see a doctor for diabetes admitted to the nursing home for dementia. Therefore, I am concerned about what I can do if diabetes causes dementia.’*  *‘My husband ... was a man who loved math. He made math out of everything. He figured out how many seconds he had to live if he lived so long, how many seconds. On the 4th May was his birthday. He was 89. We had a party for him and he died on the 20th. But that mathematical brain - he never, he didn't show any sign of forgetting.’*  *‘Make an effort. Find newspapers. Read books, Interact socially. That's why, in all of my experiences, I've seen active saintly and temple people. All of them are socially active and are all 70 to 80 years old. Why? They have a sense of faith and they're active in the community.’*  *‘I am worried about ageing, and especially about being dependent. I see my completely dependent mother and I am projecting myself.’*  *‘The premise is that it [the information] is reliable ... I have learned that information and knowledge generated at the university is reliable.’*  *‘I was very interested and enthusiastic and really quite excited about starting [the DRR intervention] because my mother suffered with dementia, so I am very aware of what the future might hold for me.’*  *‘Because I think that I have a large chance [of developing AD]. My grandmother had it and my mother also ... What we are doing, it is as if I had already been told that I have Alzheimer's, so I am already in the field.’*  *‘Well, I read that Ronald Regan had it, didn’t he, and Iris Murdoch – very well educated people – and I think you find that. Fred Fletcher from the Rotary Club, now he had it and he was a very bright chap.’*  *‘And feed your brain. I think you know, I've discovered that I don't have enough fish oil in my life or haven't been, so I started taking my fish oil again. And instead of taking what they prescribe, I find myself taking [more]. I need lots of fish and the more fish I have the better I feel and, the brighter my head is. So, and when I don't take it and there are times when I'll not take it for a week, I'm kind of, like a clock, you know, all of sudden it wounds down, so I get back on my fish oil and okay.’*  *'You know, 'cause a lot of people come down with it and they, you know, be in good shape and do what they supposed to do and eat what they supposed to eat. But still, they come down with Alzheimer's.’*  *‘Three meals a day and plenty of fruit and vegetables, he would be a veg man, lots of salads; it's definitely helped him out.’*  *‘1,000mg Vitamin E, I take one of them every day and my mind is developed considerably since I started taking them.’*  *‘I find it interesting that some people with a wonderful brain and who have done amazing work and they still develop dementia. They tell you if you keep using your brain, you are less likely to get dementia ... they are obviously still using their brain and they get it.’*  *‘We see our future through our parents. I do not want to spend my old age like my parent, who is having such a miserable life and putting so many burdens onto her children. I'm worried.’’*  *‘My mother in law took the ginsa thing. She got it anyhow. But lots of people roun’ here took yellowroot growin up ... It's supposed to be good for a lot of stuff.’*  *‘I've seen people who are, who are real active but get Alzheimer's. Real active.’*  *‘Well, they're always suggesting that you do this or you do that to keep you and you work crossword puzzles and do this and you work, do these things to keep you mind sharp and you belong to all these different things to keep your mind sharp and you exercise. But, that doesn't necessarily mean that it's gonna help because the people, some people you know and have seen, why they still get it.’*  *‘My mother is 92 years old and she's just now going into a nursing home. We just found a room for her. And she has been active, and I truly believe that eating lots of fruits and vegetables helps.’*  *‘My daddy lived to be 93 and it was because of his good work ethic. When he retired until he had his stroke he would plant a garden, slaughter hogs, cure country hams, make sausages, can. He would make us pick green beans, tomatoes and stuff. I'm convinced that's why he lived.’*  *‘I think less strain, better food and more recreation keep you from losing your mind (cultural definition of cognitive decline) and not having a lot of stress. We would go to a polka fest for a weekend deal. They were relaxing. You could forget about your problems when you were gone, even though they were still there when you got back.’*  *‘My mother did that though. She watched her diet and she was very careful and doggone it, it didn't keep it from happening.'*  *‘I am concerned that I might develop the dementia that my mother had.’*  *‘On this page we had to list everything that you ate that day, how much. And what we were watching was the sodium. And when I did that, I realised that my portions were too large.’*  *‘We’ve used spring water all these years we've been here and I'm just fine. When you moved around years ago, you got [water from] different well from different places but the town use chlorine in the water. I'm sure there's something in the food too.’*  *‘You have to concentrate on it [games]. My brother-in-law's mother was in a rest home. She was like 96. Every day they went up and played dominos with her. I mean, she would beat the socks off of them - up until they knew when she was getting ready to die, because her mind started going. And it wasn't within two weeks, she was gone. So, you know, if you just keep active and make yourself concentrate on things. I think that's it.’*  *‘But I saw that [a family member with AD] and I'm, I'm determined that I never want me or any of my loved ones to be like that.’*  *‘Normally, when you don't have any complaints, you don't visit the doctor, right? It feels unnecessary. So I'm glad we have this now, it makes me feel very safe.’*  *‘Well, I remember grandmother was, I guess she would repeat herself, so after a while they would kind of just quit listening to her and talk, talk about her, and she'd be sitting there, but they would be talking about her like she wasn't even there. I wouldn't want to be treated like that. So, it's important to me to try to remember things and not repeat things.’*  *‘I'm sure there are things that we can do to improve our health as we grow older, you know, but I think for the previous generation, the generation before us, I think they did what they could to remain healthy, they worked hard, they were caring people and they worked harder physically than our generation did, you know, and today's generation as well, I mean, they were slaves, really. It was a hard life, you know, but they accepted it and I think it contributed to their well-being.’*  *‘A feeling of better self-esteem because the things I'd let slip I picked up again and succeeded.’*  *‘I have a couple of sisters … I mean, there are too many things out there to do. Get outside and walk. They won’t – you just have to ask them, that’s all – that’s the only thing you can do.’*  *‘My attitude has changed towards my health problems and I see how a healthier diet can help my sleep.’*  *‘I did find myself, having - getting some confidence back.’*  *‘Totally de-motivating because of [the] result.’*  *‘As long as you exercise, you’ll be ok. My father doesn’t exercise. Now, he can’t even recognise his son.’*  *‘Using one's hands is the key to avoiding becoming boke. Because I have always used my hands because of my work as a tailor, I have avoided becoming boke.’*  *‘And, I just watch my mum here and I know she takes a lot of over-the-counter vitamins and things that helps her and I tell you she, she's sharp. I have to say she's sharp.’*  *‘I was hoping that if I got this [genetic risk] information I would have this great transformation … it didn’t happen like that … it was nebulous … if it had been real concrete – you know, then I’d get things planned … but nope.’*  *‘It kept my interest more at the beginning, more so than at the end because I think I was feeling I wasn't improving very much.’*  *‘Interviewees with more computer experience, in particular, thought that internet could be a useful tool to improve lifestyle.’*  *‘My mother always made us drink wildroot tea, and I don’t have a touch of it.*  *‘I think you have to keep busy – busier. I see when I go to the hospital, you know, people are sitting there and doing nothing.’*  *‘In China, we have a proverb called '3 Self's - self-content, self-belief, and self-enjoyment.’.. then there is also the 'three active: mentally active, this includes playing cards, memorizing things, being exposed to new things... a little bit of everything. Physically active - just like this Madame, who often does Tai Chi and sword exercise, and dancing... and socially active, one must socialise.’ However, a Chinese proverb says too much of anything is not good. Exercise is good, but over-exercising is not good either. So I always emphasise balance... balance.’*  *‘I quickly experienced some rewarding physical improvements.’*  *‘Maybe it [eating right] helped me; I'm still goin' strong.’*  *‘I find that if I have a piece of work to do and I'm getting too bogged down in it, if I do something that's puzzle-based then I can come back and my mind is a bit fresher.’*  *‘It has already affected my mother, but we may be all right if we change our lifestyle.*  *‘Because there are a lot of people out here that just have no interest in all these things until something happens to them. It's really a hard thing to do.’*  ‘When individuals believed that they had symptoms, they tried to take more action for disease management to prevent dementia.’  ‘The lives of the older women were informed and shaped by the values and behaviours inherent in the farming community. It is through this ‘lens’ that the older women developed perceptions about health and the implications of illness.’  ‘Some expressed uncertainty because of conflicting experiences.’  ‘In addition to goal achievement, some participants reported other or unexpected benefits from taking part in the trial beyond those specified in the individual goals. This process was empowering.’  ‘Previous family experiences of caring for someone with dementia and experiencing this as burdensome also influenced perceptions of dementia and the significance of preventing it.’  ‘Distracted walking was inevitable, creating anxiety that impeded full immersion in the social and physical experience.’  ‘[Participants] shared beliefs about home-made and home-grown food contributing to longevity in prior generations within their families.’  ‘All participants found the American accent, vocabulary and pronunciation confusing or distracting, which generally limited their engagement with the BFP.’  ‘All participants commonly skipped animations as they were considered time consuming or inappropriate to the Australian culture.’  ‘Having experienced the goal-setting process as part of the trial, there were signs that participants would continue to use this approach.’  ‘Good health in ageing is understood to be a direct outcome of life lived on the land in a time when and place where survival was reliant on the physically demanding work of subsistence and constant engagement with the geophysical world.’  ‘When a health problem occurred, they visited healthcare professionals immediately.’  ‘Several of the participants reported that the groups increased their awareness of the perceived benefits of the targeted behaviour changes. This was most frequently discussed in relation to dietary change.’  ‘The program did not seem to cause fear among participants but offered reassurance that things remained well and they were being cared for.’  ‘For all participants, the constant repetition of instructions for each exercise increased frustration and instructions would commonly be skipped.’ |
